# Molecular mechanism of cytoplasmic dynein tension sensing

**DOI:** 10.1038/s41467-019-11231-8

**Published:** 2019-07-26

**Authors:** Lu Rao, Florian Berger, Matthew P. Nicholas, Arne Gennerich

**Affiliations:** 1 Department of Anatomy and Structural Biology and Gruss Lipper Biophotonics Center, Albert Einstein College of Medicine, Bronx, NY 10461 USA; 2 Laboratory of Sensory Neuroscience, Rockefeller University, New York, NY 10065 USA; 3 Medical Scientist Training Program, Albert Einstein College of Medicine, Bronx, NY 10461 USA; 4 Present Address: Flaum Eye Institute, University of Rochester Medical Center, 210 Crittenden Blvd, Rochester, NY 14642 USA

**Keywords:** Single-molecule biophysics, Proteins

## Abstract

Cytoplasmic dynein is the most complex cytoskeletal motor protein and is responsible for numerous biological functions. Essential to dynein’s function is its capacity to respond anisotropically to tension, so that its microtubule-binding domains bind microtubules more strongly when under backward load than forward load. The structural mechanisms by which dynein senses directional tension, however, are unknown. Using a combination of optical tweezers, mutagenesis, and chemical cross-linking, we show that three structural elements protruding from the motor domain—the linker, buttress, and stalk—together regulate directional tension-sensing. We demonstrate that dynein’s anisotropic response to directional tension is mediated by sliding of the coiled-coils of the stalk, and that coordinated conformational changes of dynein’s linker and buttress control this process. We also demonstrate that the stalk coiled-coils assume a previously undescribed registry during dynein’s stepping cycle. We propose a revised model of dynein’s mechanochemical cycle which accounts for our findings.

## Abstract (web summary)

The cytoplasmic motor protein dynein senses directional tension; its microtubule-binding domains bind microtubules more strongly when under backward load. Here the authors use optical tweezers to show that the linker, buttress, and stalk domains together regulate directional tension-sensing.

## Introduction

The microtubule (MT) transport system regulates essential eukaryotic activities, including cell division, cell migration and the transport of subcellular cargoes. Cytoplasmic dynein (referred to here as dynein), is a key mediator of these activities, serving as the major generator of MT minus-end-directed motility. Dynein’s cargoes include nuclei and other organelles, proteins, mRNAs, and even viruses^1–3^. Not surprisingly, its dysfunction is implicated in a growing number of human diseases^4–10^ termed dyneinopathies^11^.

**Fig. 1:**
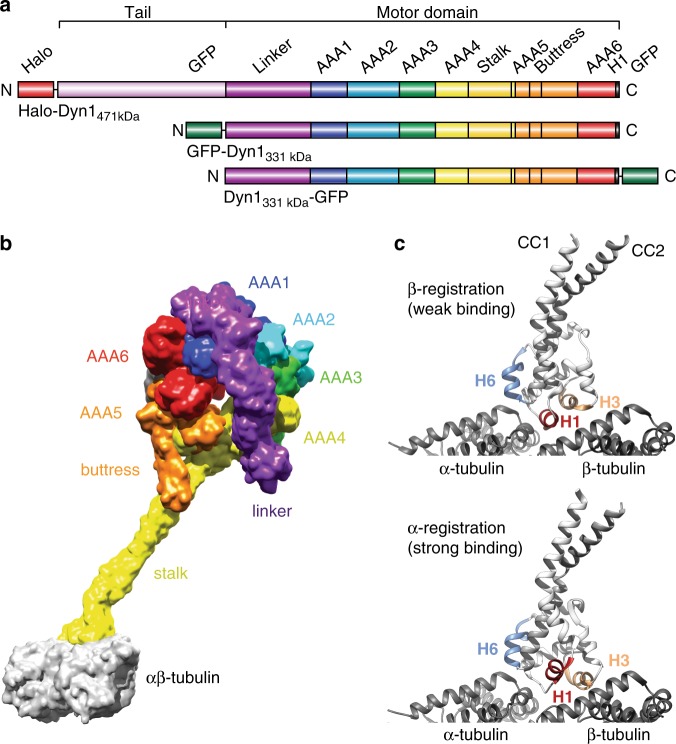
Domain organization of cytoplasmic dynein and low- and high-affinity MT-bound states of the dynein MTBD. **a** Organization of the full-length cytoplasmic dynein heavy chain (HC) with an N-terminal Halo-Tag, Halo-Dyn1_471kDa_ (a.a. 1–4092) and the tail-truncated monomeric constructs, GFP-Dyn1_331kDa_ and Dyn1_331kDa_-GFP (a.a. 1219–4092). **b** Dynein MD bound to α/β-tubulin in the strong binding state (merged from PDB entries 3VKG and 3J1T; see Supplementary Note 1). **c** MT-bound MTBD in the weak MT-binding β-registration of the stalk helices (top, PDB entry 3J1U) and in the strong MT-binding α-registration (bottom, PDB entry 3J1T)

Dynein is a AAA+ ATPase (AAA: ATPase associated with various cellular activities^12^) and is the largest and most complex cytoskeletal motor protein. It is a ~1.4 MDa protein complex composed of two identical heavy chains (HCs) and several other associated chains and accessory proteins that regulate dynein and bind it to cargo^1^. Each HC contains a C-terminal ring-shaped motor domain (MD) composed of six tandem-linked AAA modules (AAA1-6), the first four of which (AAA1–4) can hydrolyze and/or bind nucleotide^13,14^ (Fig. 1a, b). By contrast, the other cytoskeletal motors, kinesin^15–17^ and myosin^18,19^, have one ATPase per HC. While their ATPase cores directly interact with their cytoskeletal tracks, in dynein, a ~15-nm coiled-coil (CC) stalk emerges from AAA4^13^ and separates dynein’s MT-binding domain (MTBD) from the AAA ring domain^20^ (Fig. 1a, b). A second antiparallel coiled-coil called the buttress^20^ (or strut^21^) protrudes from AAA5 and contacts the stalk (Fig. 1b). Finally, a ~10-nm linker connects the N-terminal dimerizing tail domain to the AAA ring. The linker undergoes cyclic conformational changes that generate unidirectional motion and force^22,23^ (Fig. 1b).

The stalk acts as a bidirectional communication pathway between the AAA ring and MTBD: nucleotide state affects MT binding and vice versa^24,25^. The registrations of the stalk coiled-coil (CC) helices (CC1 and CC2) influence both dynein’s MT-binding affinity and the ATPase activities of the MD^26,27^. In solution, the stalk helices predominately assume a low-affinity registration called the β+ registration^27,28^ (referred to as β hereafter), and upon MT binding, transition to a high-affinity (α) registration^29,30^ (Fig. 1c). Communication along the stalk requires stalk-buttress interactions, as deletion of the buttress uncouples ATPase activity from MT binding^14,31^. The linker also mediates communication between the AAA ring and MTBD, as preventing interactions between the linker and AAA5 prevents ATP-induced MT release^13^. However, how the stalk, buttress and linker work together to regulate dynein’s MT interactions remains unknown.

**Fig. 2:**
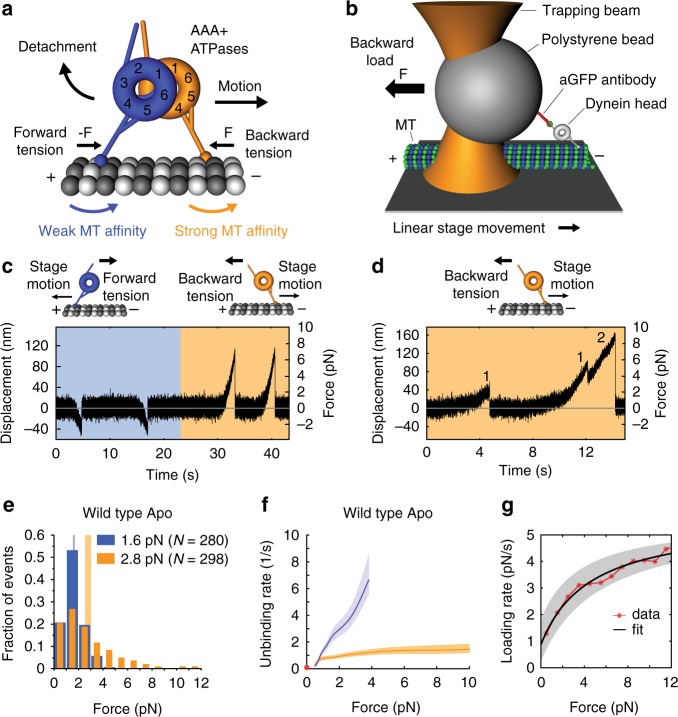
Dynein-MT bond anisotropy. **a** Model for tension-based regulation of dynein stepping. Splaying of the dynein heads generates intramolecular tension. Under backward tension (front head) MT-binding strength is greater, and under forward tension (rear head) it decreases. **b** A polystyrene bead bearing a dynein MD is held in an optical trap as the microscope stage sweeps back and forth parallel to a MT (not to scale). **c** Force (position) vs. time for WT dynein (GFP-Dyn1_331kDa_) in the apo state (see “Methods” section) acquired under non-reducing conditions. Orange and blue areas are periods of applied backward and forward tension, respectively. **d** Primary and secondary unbinding events. Event 1 is a primary event, beginning from zero force (*F*_start_ = 0). Secondary events (2) occur when the MD rebinds the MT before returning to the trap center, and thus *F*_start_ > 0. **e** Normalized histograms of primary forward (blue) and backward (orange) unbinding forces, with mean values noted (we plot the absolute force values to facilitate comparison of the unbinding-force distributions in both loading directions). Vertical bands are 95% CIs of the mean (forward: [1.5, 1.7] pN, backward: [2.5, 3.0] pN) estimated by bootstrapping 4,000 samples (source data are provided as a Source Data file). **f** Unbinding rate vs. force derived from the data in (**e**). The shaded areas are 95% CIs for the mean rates, estimated by bootstrapping. The depicted zero-load unbinding rate of 0.086 ± 0.002 s^−1^ (±SE, red circle) represents the inverse of the time constant obtained from the analysis of the experimental CDF of the MT-bound lifetimes measured via TIRF microscopy (Supplementary Fig. 2a) (the 95% CIs of the measured unbinding rate (lower limit, 0.084/s; upper limit, 0.11/s) are not shown as they are shorter than the height of the symbol). **g** Loading rate vs. force obtained from line fits to the 200-ms data segment before detachment of each measured unbinding-force event (see Supplementary Fig. 3 for underlying data)

We are only beginning to understand how dynein moves processively (the ability to take multiple steps before dissociating) against opposing forces. To move processively, one MD (head) must bind the MT tightly while the other head detaches and advances (Fig. 2a). Dynein accomplishes this in part by employing a tension-direction-dependent MT-binding strength^32,33^. In nucleotide-free conditions, when force is applied to a MD in the forward direction (when the MD is pulled toward the MT minus-end), it forms a slip bond with MTs, exhibiting faster unbinding with increasing tension. However, when the MD is subjected to a hindering or backward load (pulled toward the MT plus end), it forms a slip-ideal bond with MTs, exhibiting faster unbinding for backward forces up to ~2 pN and constant, force-independent unbinding rates for greater forces^32^. This anisotropic MT-binding behavior explains why the likelihood for trailing head detachment (and subsequent forward movement) increases with the distance between the two heads^34,35^. Thus, interhead tension, which increases as the heads spread apart, helps keep the dynein heads out-of-phase^15^. These results imply that both external load and intramolecular tension contribute to the control of dynein motion along MTs.

By what structural mechanisms does tension alter dynein function? What roles do the stalk, buttress and linker play in dynein’s response to load? Here we combine structure-function, mutagenesis and chemical cross-linking with single-molecule optical tweezers studies to answer these questions. We demonstrate that the anisotropic effect of tension on the MT-binding strength arises from force-induced sliding of the helices in the stalk, and that coordinated conformational changes of dynein’s linker and buttress control this process. In addition, we show that dynein assumes a previously undescribed stalk registration under forward load in the nucleotide-free state which we term the γ-registration. Our results illustrate how, in response to tension, a complex interplay between the linker, stalk, and buttress control conformational changes in the MTBD, facilitating the coordinated movements of dynein’s MDs.

## Results

### Tension regulates MT-binding via stalk helix sliding

We previously demonstrated that a single dynein head responds anisotropically to tension^32^. Consistent with other reports^33,36^, a dynein head binds tighter to MTs when pulled toward the MT plus end (Fig. 2b–f). We reasoned that this behavior might result from tension-induced reconfiguration of the coiled-coil stalk and/or force-induced changes in the MTBD or MT lattice^32^. Since the stalk configuration is known to influence MT affinity in the absence of load^26,27^, we first tested whether dynein’s anisotropic MT-binding strength is caused by a tension-direction-dependent sliding of dynein’s stalk helices.

To prevent tension-induced stalk helix sliding, we introduced cysteine residues in the outgoing (CC1) and return (CC2) α-helices of the stalk of Dyn1_331kDa_, a minimal *S. cerevisiae* MD that contains the linker and the AAA ring, and the stalk and MTBD. Dyn1_331kDa_ retains its motor activities^32,33,37^ and is equivalent to the *Dictyostelium discoideum* MD used in key biochemical studies^22,26,38–41^ (see Supplementary Fig. 1a for sequence alignment). The addition of the cysteine residues enabled reversible disulfide cross-linking. Under non-reducing conditions (without DTT) following oxidation^26^, the stalk helices were cross-linked with an efficiency of >95% (Supplementary Note 2). Since our previous optical tweezers-based unbinding-force experiments were performed under reducing conditions^32^, we repeated the experiments with WT dynein and found that WT motors showed anisotropic responses under both reducing^32^ and non-reducing conditions (Fig. 2c–f). Thus, we can compare the anisotropic behavior of WT dynein with the tension response of the cross-linked constructs under non-reducing conditions.

**Fig. 3:**
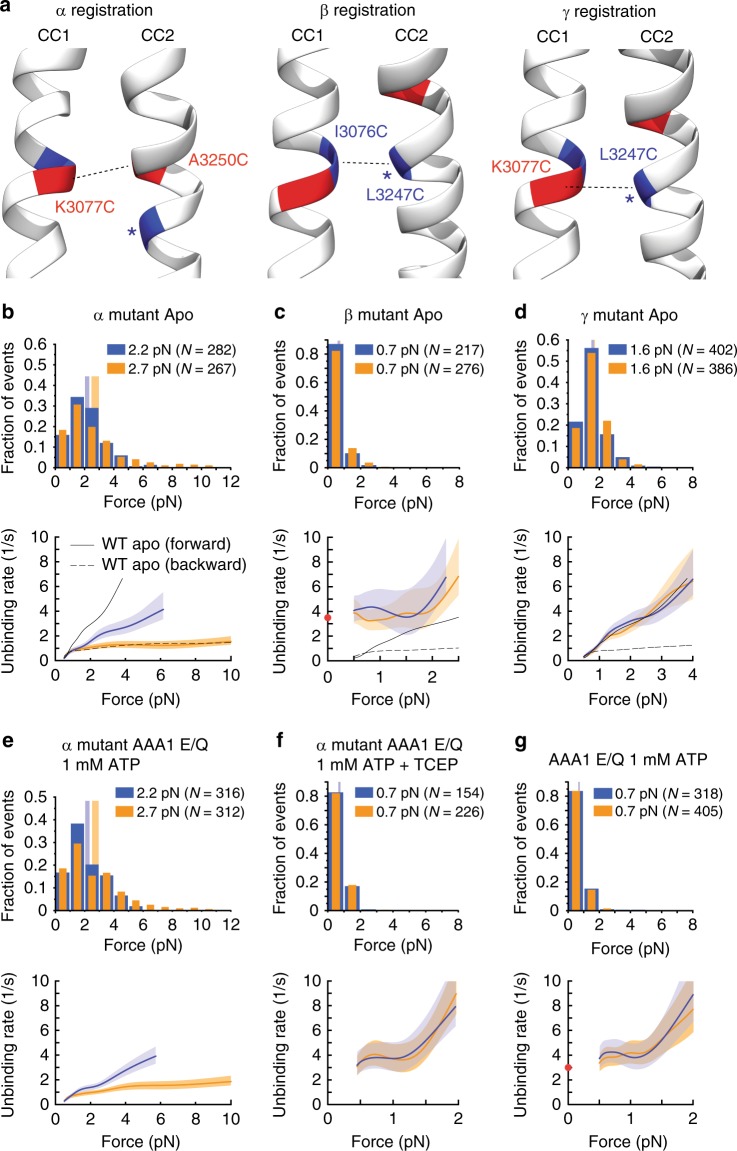
Tension direction alters dynein MT-binding strength via sliding of the stalk helices. **a** High-affinity (α), low-affinity (β) and intermediate-affinity (γ) helix registrations of the dynein stalk generated from the morphing structure described in the Supplementary Note 1. **b**, top: Normalized histograms of primary forward and backward unbinding forces for the Dyn1_331kDa_-α CL mutant, with mean values noted (95% CIs [2.0, 2.3] and [2.5, 3.0] pN, estimated by bootstrapping 4000 samples). **b**, bottom: Unbinding rate vs. force derived from the data above. The shaded areas are 95% CIs for the mean rates, estimated by bootstrapping. **c** As in (**b**), but for the Dyn1_331kDa_-β CL mutant (95% CIs [0.6, 0.7] and [0.7, 0.8] pN). The depicted zero-load unbinding rate of 3.5 ± 0.1 s^−1^ (characteristic rate ± SEM) (red circle) represents the inverse of the time constant obtained from the CDF analysis of the MT-bound lifetimes measured via TIRF microscopy (Supplementary Fig. 2c) (the 95% CIs of the measured unbinding rate (lower limit, 3.3/s; upper limit, 3.6/s) are not shown as they are shorter than the height of the symbol). **d** As in **b**, but for the Dyn1_331kDa_-γ CL mutant (95% CIs [1.5, 1.7] and [1.6, 1.7] pN). **e** As in (**b**), but for the AAA1 E/Q Dyn1_331kDa_-α CL mutant and 1 mM ATP (95% CIs [2.0, 2.3] and [2.5, 3.0] pN). **f** As in (**e**), but with 2 mM TCEP to cleave the disulfide bonds of the stalk helices (95% CIs [0.6, 0.8] and [0.7, 0.8] pN). **g** As in (**b**) but for the AAA1 E/Q Dyn1_331kDa_ mutant and 1 mM ATP (95% CIs [0.7, 0.7] and [0.7, 0.7] pN). The depicted zero-load unbinding rate of 3.0 ± 0.1 s^−1^ (red circle) was derived from Supplementary Fig. 2d as described for the zero-load unbinding rate in **c** (the 95% CIs of the measured unbinding rate (lower limit, 2.8/s; upper limit, 3.1/s) are not shown as they are shorter than the height of the symbol). For comparison, the force-dependent unbinding rates of the WT motor in the apo state are shown in (**b**–**d**) (dashed and solid black lines). Source data are provided as a Source Data file

We then cross-linked the stalk helices in the high-affinity α-registry (K3077C and A3250C, Supplementary Fig. 1b) (Fig. 3a, b) and the low-affinity β-registry^27^ (I3076C and L3247C, Supplementary Fig. 1b) and measured the unbinding behaviors of the cross-linked (CL) constructs (Fig. 3a, c). Cross-linking the stalk helices in the α-registration significantly reduces dynein’s anisotropic response to directional tension in the absence of nucleotides (*p*_m_ < 10^–10^ [Dyn1_331kDa_ apo forward vs. Dyn1_331kDa_ apo backward], and *p*_m_ = 0.03 [Dyn1_331kDa_-α CL apo forward vs. Dyn1_331kDa_-α CL apo backward] (Figs. 2e, f and 3a, b), with *p*_m_ being an estimate for the *p-*value for the difference of the means, see “Methods” section). When locked in the α-registration, dynein’s slip bond with the MT under forward load became significantly stronger (Fig. 2e, f) (Dyn1_331kDa_ apo forward 1.6 [1.5, 1.7] pN vs. Dyn1_331kDa_-α CL apo forward 2.2 [2.0, 2.3] pN, Fig. 3b). Under backward load, Dyn1_331kDa_-α CL exhibited slip-ideal bonding statistically indistinguishable from WT Dyn1_331kDa_ (*p*_ks_ **=** 0.83 [Dyn1_331kDa_-α CL apo backward vs. Dyn1_331kDa_ apo backward], with *p*_ks_ being the *p-*value resulting from a two-sample Kolmogorov–Smirnov (KS) test of the cumulative distribution functions (CDFs) calculated from the measured histograms of primary unbinding forces, see “Methods” section). In contrast, cross-linking the stalk helices in the β-registration caused dynein to respond isotropically to tension in nucleotide-free conditions (Fig. 3a, c). The β-registration resulted in weak MT-binding in both directions (forward 0.7 [0.7, 0.8] pN vs. backward 0.7 [0.6, 0.7] pN, *p*_ks_ = 0.59, Fig. 3c).

### The β-registration results in an ideal-slip bond with MTs

The force-dependent unbinding rates of the dynein β-mutant remain relatively constant in both directions for forces up to ~1.5 pN (ideal bonding) and then increase with increasing load (slip bonding) (Fig. 3c, bottom). To determine whether the β-mutant also exhibits ideal-bond behavior even down to zero load (as suggested by the unbinding rates for ~0.5–1.5 pN), we used a total internal reflection fluorescence (TIRF) assay to determine the unbinding rate of the β-mutant at zero load. Using surface-immobilized MTs, we imaged the binding and dissociation of our β-mutant heads (labeled with Cy3 via an inserted N-terminal SNAP-tag) at 100 ms/frame and determined an average MT-bound time of ~290 ms, corresponding to an unbinding rate of 3.5 ± 0.1 s^−1^ (mean ± SEM; Supplementary Fig. 2b and Fig. 3c, red circle). The unbinding rate falls within the CIs of the unbinding rates in the ~0.5–1 pN force range and is therefore consistent with an ideal-bond behavior down to zero load. Of note, recent studies by the Howard and Ostap labs have shown that the vertical force component in single-bead assays can increase the detachment rate of kinesin motors^42,43^. As a dynein head could also be sensitive to vertical forces, it is possible that the zero-force unbinding rate projected based on the apparent ideal-bond behavior in the ~0.5–1.5 pN force range overestimates the zero-force detachment rate. In conclusion, the dominant unbinding behavior of the β-mutant is largely insensitive to forces below ~1.5 pN and increases rapidly with force above this value, a behavior we term ideal-slip bonding.

### ATP-bound dynein assumes the β-registration

It is known that ATP binding to AAA1 induces a weak MT-binding state. We hypothesized that this is mediated by the stalk sliding into the β-registration. To test this, we compared the bond behaviors of the non-cross-linked WT motor bearing an E/Q mutation in the AAA1 Walker B motif (which allows ATP binding but prevents ATP hydrolysis) with the behavior of the β-mutant. In the presence of ATP, AAA1 E/Q Dyn1_331kDa_ shows markedly weaker unbinding forces (mean < 1 pN) in both directions compared to nucleotide-free conditions (Figs. 2e and 3g)^32^. Similar to the cross-linked β-mutant, the non-cross-linked AAA1 E/Q Dyn1_331kDa_ mutant in the presence of ATP exhibits slip bond behaviors in both directions for forces above ~1 pN and a similar zero-load unbinding rate (3.0 ± 0.l s^−1^, Supplementary Fig. 2c and Fig. 3c, g). In contrast, the cross-linked AAA1 E/Q α-mutant shows ATP-insensitive slip bonding under forward load and slip-ideal bonding under backward load (Fig. 3e), with statistically indistinguishable unbinding-force behavior from the Dyn1_331kDa_-α CL motor in the apo state (*p*_ks_ = 0.88 [AAA1 E/Q Dyn1_331kDa_-α CL ATP forward vs. Dyn1_331kDa_-α CL apo forward] and *p*_ks_ **=** 0.94 [AAA1 E/Q Dyn1_331kDa_-α CL ATP backward vs. Dyn1_331kDa_-α CL apo backward], Fig. 3b, e). MT-binding sensitivity to ATP is restored to the AAA1 E/Q α-mutant when 2 mM TCEP (a reducing agent that breaks disulfide bonds) is added to reverse the cross-linking of the stalk helices. With TCEP present, ATP induces markedly weaker unbinding forces (mean < 1 pN) in both directions (Fig. 3f). These studies demonstrate that ATP binding to AAA1 induces the β-registration.

### Forward load induces an intermediate stalk registry

Notably, under forward load (when pulled toward the MT minus-end) in the absence of ATP, the mean unbinding force of Dyn1_331kDa_ (1.6 pN) (Fig. 2e) is significantly larger than that of Dyn1_331 kDa_-β CL (0.7 pN) (Fig. 3c) (*p*_m_ < 10^–10^). This suggests that the apo-state WT motor assumes a stalk configuration under forward load that is distinct from the β and α-registrations. We hypothesized that this stalk helix registration is a registration in between the weak (β) and strong (α) MT-binding registries, which we name the γ-registration (Fig. 3a). To test our hypothesis, we cross-linked the stalk helices a half registry in between the β- and α-registrations, so that K3077 of CC1 aligns with L3247 of CC2 (Fig. 3a, right, and Supplementary Fig. 1b). Cross-linking in the γ-registry results not only in a direction-independent slip bond with the MT (as observed for the apo-state WT MD under forward load; Fig. 2f) (*p*_ks_ = 0.2 [Dyn1_331 kDa_-γ CL forward vs. Dyn1_331 kDa_-γ CL backward], Fig. 3d), but also in MT-binding strengths statistically indistinguishable from the unbinding-force distribution of the apo-state WT motor under forward load (*p*_ks_ = 0.43 [Dyn1_331 kDa_-γ CL forward vs. Dyn1_331kDa_ apo forward], Figs. 2e and 3d). Thus, taken together, our results suggest that backward load induces the α-registration, while forward load induces a previously undescribed γ-registry in the absence of nucleotides, and upon ATP binding to AAA1, the stalk assumes the β-registry.

### MT-binding asymmetry involves stalk sliding and the AAA ring

**Fig. 4:**
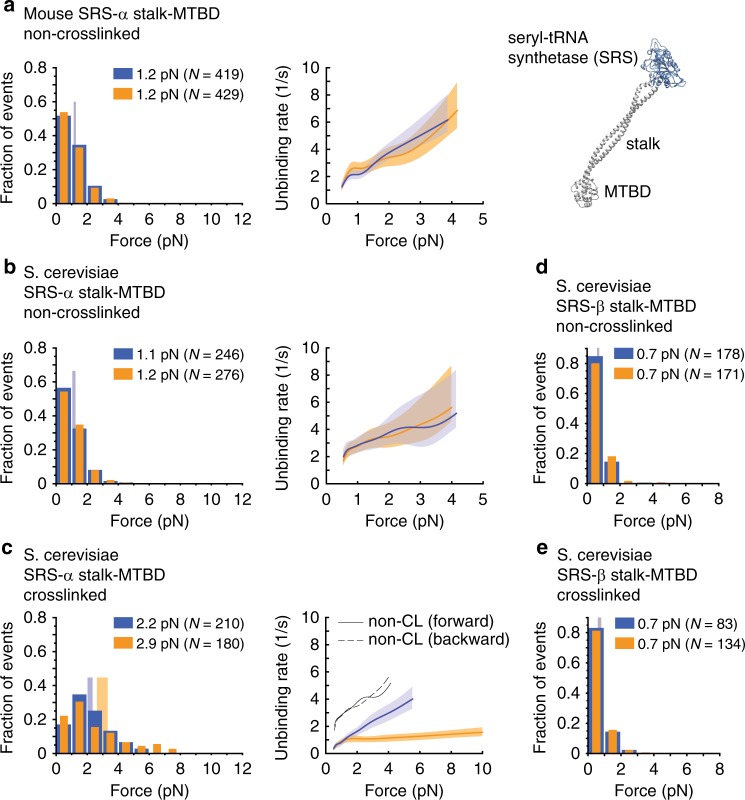
Unbinding-force behaviors of the SRS-stalk-MTBD constructs. **a**, left: Normalized histograms of primary forward and backward unbinding forces for the SRS construct with the mouse stalk helices (a.a. 3019–3309) fused in the non-cross-linked α-registry (SRS 85:82), with mean values noted (95% CIs [1.1, 1.3] and [1.1, 1.3] pN, estimated by bootstrapping 4,000 samples). **a**, middle: Unbinding rate vs. force derived from the data on the left. The shaded areas are 95% CIs for the mean rates, estimated by bootstrapping. **a**, right: Model of monomeric *T. thermophilus* seryl-tRNA synthetase (SRS) (based on PDB entry 3ERR) fused to the near-full-length stalk and MTBD of dynein in the β-registry of the stalk helices (generated by aligning PDB entry 3WUQ and 4RH7, see Supplementary Note 1). **b** As in (**a**) but for the SRS construct with the *S. cerevisiae* stalk helices (a.a. 3019–3309) fused in the non-cross-linked α-registry (95% CIs [1.0, 1.2] and [1.1, 1.2] pN). **c** As in (**b**) but with the stalk helices cross-linked (K3077C, A3250C) in the α-registration (95% CIs [2.0, 2.4] and [2.6, 3.3] pN). **d** As in (**b**) but for SRS fused to the *S. cerevisiae* stalk and MTBD in the weak MT-binding β-registry (SRS 89:82) (a.a. 3015–3309) (95% CIs [0.6, 0.7] and [0.6, 0.7] pN). **e** As in (**d**) but with the stalk helices cross-linked (I3076C, L3247C) in the β-registration (95% CIs [0.6, 0.7] and [0.7, 0.8] pN). Source data are provided as a Source Data file

We previously demonstrated that ATP binding to AAA1 (Fig. 3g) and ADP binding to AAA3 both reduce bond-strength anisotropy, while ADP binding to AAA1 strengthens it^32^, suggesting the AAA ring regulates bond strength anisotropy. However, Cleary and coworkers reported results suggesting the AAA ring was not needed for dynein’s anisotropic response to directional tension. When the mouse stalk and MTBD was fused to the coiled-coil base of *T. thermophilus* seryl-tRNA synthetase (SRS) in the α-registry (SRS-α stalk-MTBD), significant bond-strength anisotropy was observed^33^ (these chimeric fusion constructs were used by Gibbons et al. to establish the α- and β-registries^27^; Fig. 4a, right). This result suggested that bond-strength anisotropy is intrinsic to the stalk and MTBD, and not dependent on other structural elements, such as the AAA ring, linker, or buttress. In contrast, we did not observe significant bond-strength anisotropies for the SRS-α stalk-MTBD fusion construct with the mouse stalk-MTBD sequence (*p*_ks_ = 0.38 [forward vs. backward]; Fig. 4a) or the yeast stalk-MTBD sequence (*p*_ks_ = 0.62 [forward vs. backward]; Fig. 4b) in the absence of stalk cross-linking. To determine the reason for this discrepancy, we repeated the experiments with the mouse SRS-α stalk-MTBD construct on axonemes, the substrate used by Cleary and coworkers^33^. As in the case of MTs, we observed no anisotropic MT-binding strength (*p*_ks_ = 0.62 [forward vs. backward]; Supplementary Fig. 4a). The cause for the different outcomes must therefore lie in the different unbinding-force assays used. Indeed, when we applied the oscillatory assay (Supplementary Note 3) used by Cleary et al. (in contrast to the constant-pulling assay used herein, Methods), we were able to reproduce the anisotropic unbinding behavior reported by Cleary and coworkers^33^ (Supplementary Figs. 5–7).

Notably, the unbinding rates obtained from the two assays differed only under forward load (Supplementary Fig. 7). We wondered what might account for the significant increase in unbinding rates under forward load in the oscillatory assay compared to the constant-pulling assay. In the oscillatory assay, the trap moves very rapidly between two positions (±250 nm) at an initial speed of ~1 mm/s (see Supplementary Note 4), whereas in the constant-pulling assay used herein, the coverslip-attached MT moves at a constant velocity of only ~100 nm/s past the stationary trapped bead. In the oscillatory assay, the binding of the SRS-stalk-MTBD construct to the MT most likely occurs during the extended dwelling of the bead in between the switching events^44^. It is therefore possible that the extremely high loading rates at the beginning of the trap displacement modify the unbinding pathway of the MTBD-MT bond when the load is applied in the forward direction and reduce the bond lifetime (rates of up to ~25,000 pN/s can be produced, see Supplementary Note 4 and Supplementary Fig. 8).

Further comparison of the SRS-α stalk-MTBD construct using the constant-pulling assay reveals that an anisotropic response to directional tension is only observed when the stalk helices are cross-linked in the α-registration (Fig. 4c). Notably, the cross-linked SRS-α stalk-MTBD construct shows statistically indistinguishable behavior from the dynein MD with the cross-linked α-registration, Dyn1_331 kDa_-α CL (Fig. 3b and Fig. 4c). In contrast, the frequently used non-cross-linked SRS-α stalk-MTBD construct shows significantly different unbinding behavior from the cross-linked SRS-α stalk-MTBD construct (*p*_ks_ < 10^−21^ [SRS-α stalk-MTBD forward vs. SRS-α stalk-MTBD CL forward], and *p*_ks_ < 10^−19^ [SRS-α stalk-MTBD backward vs. SRS-α stalk-MTBD CL backward], Fig. 4b, c), suggesting that the non-cross-linked SRS-α stalk-MTBD construct does actually not assume the α-registration. However, the SRS-β stalk-MTBD construct shows the same unbinding behaviors whether the stalk helices are cross-linked in the β-registration or not (*p*_ks_ = 0.76 [SRS-β stalk-MTBD forward vs. SRS-β stalk-MTBD CL forward] and *p*_ks_ = 0.42 [SRS-β stalk-MTBD backward vs. SRS-β stalk-MTBD CL backward], Fig. 4d, e). This suggests that the SRS-β stalk-MTBD construct assumes the β-registration with or without stalk cross-linking. We conclude that the constant-pulling assay used herein provides accurate data also when analyzing the short SRS fusion constructs.

Together, our data indicate that either tension-induced helix sliding is somehow prevented in the non-cross-linked SRS constructs when bound to the MT, or that other elements, such as the AAA ring, buttress, and linker, are required for registration changes of the stalk helices in response to directional tension (for a detailed discussion on why the stalk helices of the SRS-α stalk-MTBD construct can be cross-linked in different registrations in solution while directional tension alone is incapable of inducing registration changes, see Supplementary Note 5). We sought to further understand how tension induces helix sliding and to elucidate the roles of the AAA ring and its appendages in this process.

### Tension-induced stalk sliding requires the buttress

**Fig. 5:**
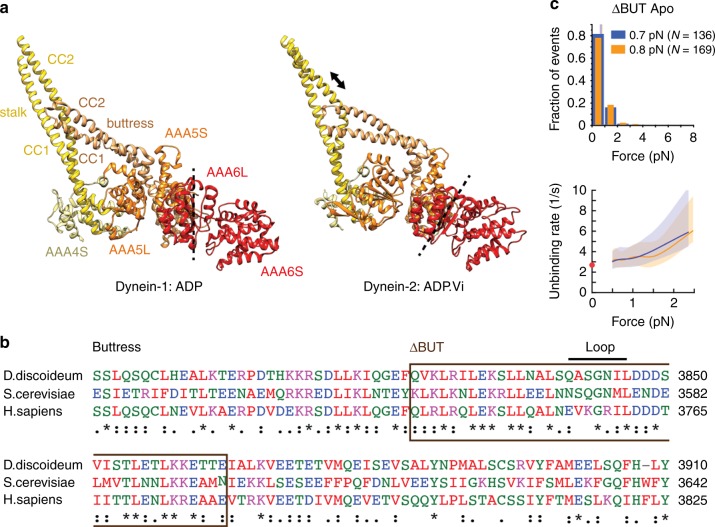
Tension-induced registry changes of the stalk helices require stalk-buttress interactions. **a** A comparison of the crystal structures of dynein-ADP (PDB entry 3VKG^14^) and dynein-ADP.Vi (PDB: 4RH7^47^) suggests that ATP binding to AAA1 causes a rotation of AAA6L/AAA5S (the dashed line indicates the orientation of AAA6L), resulting in displacement of the buttress (orange) and sliding of CC2 relative to CC1 (arrow), which in turn induces the low-affinity MT-bound state^45^. **b** Alignment of buttress amino acid sequences. Deletion is highlighted. **c**, top: Normalized histograms of primary forward and backward unbinding forces for the ΔBUT-Dyn1_331kDa_ mutant (top), with mean values noted (95% CIs [0.6, 0.8] and [0.7, 0.8] pN, estimated by bootstrapping 4,000 samples) (source data are provided as a Source Data file). **c**, bottom: Unbinding rate vs. force derived from the data above. The shaded areas are 95% CIs for the mean rates, estimated by bootstrapping. The depicted zero-load unbinding rate of 2.67 ± 0.05 (characteristic rate ± SEM) (red circle) represents the inverse of the time constant obtained from the analysis of the experimental CDF of the MT-bound lifetimes measured via TIRF microscopy (Supplementary Fig. 2e) (the 95% CIs of the measured unbinding rate (lower limit, 2.58/s; upper limit, 2.77/s) are not shown as they are shorter than the height of the symbol)

In the absence of load, nucleotide state and MT binding affect one another^24,25,27,38^ via helix sliding^26^, and helix sliding is facilitated by the coiled-coil buttress^14^ (Fig. 5a). ATP binding to AAA1 causes closure of the AAA ring, forcing subdomains of AAA6 and AAA5 to rotate^45^ (Fig. 5a). This rotation displaces the buttress, which in turn appears to induce sliding of the stalk helices and transition from a strong to a weak MT-binding state (Figs. 1c and 5a). We hypothesized that the buttress also facilitates tension-induced helix sliding. To test this, we prevented stalk-buttress interactions by replacing the distal end of the buttress with a flexible linker (GSGS), similar to a construct used previously to uncouple ATPase activities from MT binding^14^ (ΔBUT, Fig. 5b). As expected, this mutant shows weak and symmetric unbinding forces in the apo state (*p*_ks_ = 0.53, forward vs. backward) and mean unbinding forces statistically indistinguishable from Dyn1_331kDa_-β CL (*p*_m_ = 0.31 (forward) and *p*_m_ = 0.36 (backward), Figs. 3c and 5c). Thus, the tension-induced anisotropy of the dynein-MT bond depends on tension-induced conformational changes within the AAA ring that are transmitted to the stalk via the buttress.

### AAA5-linker interactions control stalk helix sliding

**Fig. 6:**
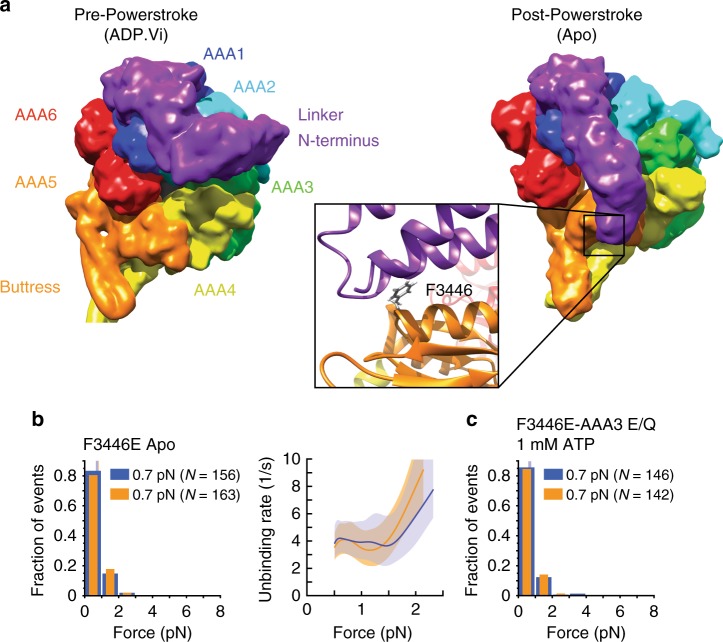
Docking/undocking of the linker to and from AAA5 controls tension-induced reconfiguration of the stalk. **a** Structures of the dynein MD in the pre-powerstroke (ADP.Vi, *Homo sapiens* cytoplasmic dynein-2; PDB entry 4RH7^47^) and post-powerstroke (Apo, *S. cerevisiae* dynein; PDB entry 4W8F^13^) states. In the pre-powerstroke state, the linker is bent and close to AAA2 (left), while in the post-powerstroke state, the linker is straight and docked on AAA5 (right). (inset) Interactions between hydrophobic residues of the linker and the highly conserved F3446 of AAA5 facilitate the docking of the linker N-terminus on AAA5^13^ (PDB entry 4W8F^23^). **b**, left: Normalized histograms of primary forward and backward unbinding forces for the F3446E-Dyn1_331 kDa_ mutant, with mean values noted (95% CIs [0.7, 0.8] and [0.7, 0.8] pN, estimated by bootstrapping 4000 samples). **b**, right: Unbinding rate vs. force derived from the data on the left. The shaded areas are 95% CIs for the mean rates, estimated by bootstrapping. **c** As in **b**, but for the F3446E-AAA3 E/Q Dyn1_331 kDa_ CT-GFP mutant and 1 mM ATP (95% CIs [0.6, 0.8] and [0.6, 0,7] pN). Source data are provided as a Source Data file

In the nucleotide-free, post-powerstroke state, the linker contacts conserved residues in AAA5^13^ (Fig. 6a, b). As disruption of the linker-docking site in AAA5 severely reduces MT-activated ATPase activity, ATP-induced release from MTs, and dynein motility^13^, we sought to determine whether the conformation of the linker affects the buttress-mediated sliding of the stalk helices.

Comparison of the apo-state unbinding-force histograms acquired under forward load of Dyn1_331 kDa_ (Fig. 2e) and Dyn1_331kDa_-β CL (Fig. 3c) shows that forward tension alone is incapable of changing the γ-registration of the stalk helices (Fig. 3d) into the β-registration (Fig. 3c). However, ATP binding to AAA1 results in mean unbinding forces statistically indistinguishable from the β-mutant (*p*_m_ = 0.31 (forward) and *p*_m_ = 0.79 (backward) [AAA1 E/Q Dyn1_331 kDa_ ATP vs. Dyn1_331 kDa_-β CL], Fig. 3c, g). Since ATP binding to AAA1 causes the transition from the post- to the pre-powerstroke conformational state of the linker^22^, we hypothesized that linker docking to AAA5 in the post-powerstroke linker conformation blocks transition into the β-registration. To test this idea, we mutated the highly conserved residue F3446 in the AAA5 linker-docking site (Fig. 6a, inset, and Fig. 6b) into glutamic acid, which impairs MT-activated ATPase activity^13^. Indeed, this mutant, F3446E-Dyn1_331 kDa,_ shows β-mutant-like mean unbinding forces (*p*_m_ = 0.17 (forward) and *p*_m_ = 0.35 (backward) [F3446E-Dyn1_331 kDa_ ATP vs. Dyn1_331 kDa_-β CL], Figs. 3c and 6b).

The interactions between the linker and AAA5 could sterically or allosterically affect the base of the buttress and thereby affect the buttress’s interactions with the stalk helices. Alternatively, tension applied to the linker-AAA5 interface could induce a buttress conformation that prevents transition into the β-registration. To distinguish between these possibilities, we determined the unbinding-force behavior of the AAA3 E/Q mutant in the presence of 1 mM ATP while using a C-terminal GFP for the coupling to trapping beads in order to prevent tension from acting directly on the linker (AAA3 E/Q Dyn1_331kDa_ CT-GFP). We have previously shown that this mutant shows a strong and anisotropic unbinding behavior in the presence of ATP similar to the WT MD in the apo state^32^. In addition, Bhabha et al. showed that blocking nucleotide hydrolysis at AAA3 prevents the linker from moving from the post-powerstroke to the pre-powerstroke conformation^23^. Thus, we hypothesized that AAA5-linker interactions, as a result of the post-powerstroke linker conformation, prevent the AAA3 E/Q mutant from showing the weak MT-binding seen in the WT MD under C-terminally applied tension in the presence of ATP^32^. To test this, we induced a F3446E mutation in AAA5 in the AAA3 E/Q Dyn1_331 kDa_ CT-GFP mutant to prevent functional stalk-AAA5 interactions. As hypothesized, this mutant binds MTs weakly in both directions (*p*_ks_ = 0.82, forward vs. backward, Fig. 6c) and shows similar mean unbinding forces as the Dyn1_331kDa_ motor construct with the equivalent F3446E point mutation (*p*_m_ = 0.25 (forward) and *p*_m_ = 0.1 (backward) [F3446E-AAA3 E/Q CT-GFP ATP vs. F3446E-Dyn1_331kDa_ apo]; Fig. 6b–c). In conclusion, linker-AAA5 interactions in the absence of linker-applied tension are sufficient to block the tension-induced transition into the β-registration.

### Dynein motility requires buttress-stalk interactions

**Fig. 7:**
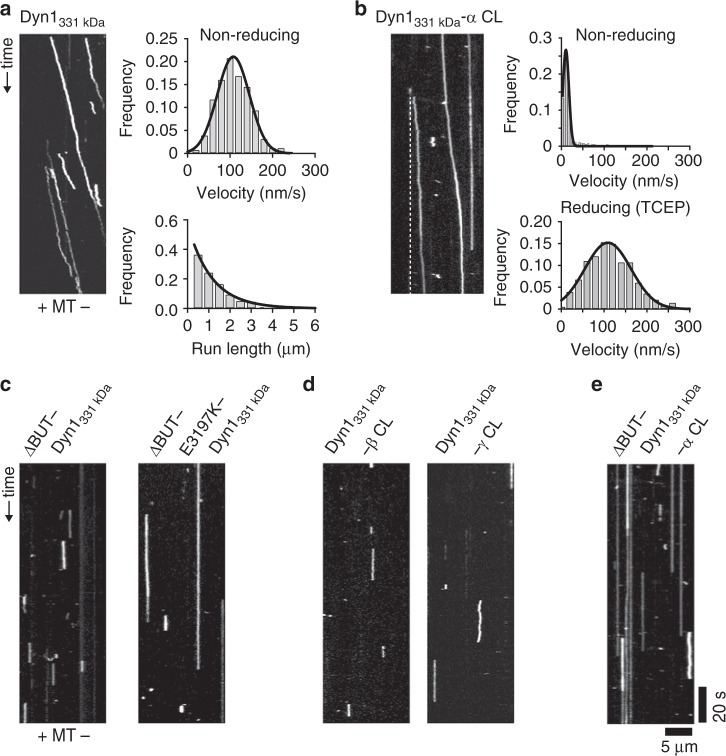
Stalk-buttress interactions are essential for dynein motion. **a** Antibody-based dimerization of WT Dyn1_331kDa_ results in processive runs under non-reducing conditions in the single-molecule TIRF assay at 1 mM ATP (left). Diagonal lines in the kymograph represent dimerized dynein molecules that are moving over time. Fitting the histogram of measured velocities with a Gaussian (black line, top right), results in a mean velocity of 108 ± 2 nm/s (±SEM; *N* = 255). A histogram of measured run lengths fit with an exponential function (black line, bottom right) yields a characteristic run length of 1.1 ± 0.1 μm (±SEM; *N* = 255) (source data are provided as a Source Data file). **b** In contrast to the motors with the cross-linked β and γ stalk registrations (**d**), Dyn1_331 kDa_-α CL shows highly processive motion (left) (the dashed white line serves as a visual guide to help recognize the slow moving Dyn1_331kDa_-α CL mutant) under non-reducing conditions. Fitting the histogram of measured velocities with a Gaussian (black line, top right), yields a mean velocity of 10.6 ± 0.4 nm/s (±SEM; *N* = 336) (top right). Performing the experiments in the presence of 2 mM TCEP increases the velocity to 109 ± 3 nm/s (±SEM; *N* = 281) (bottom right), demonstrating the reversibility of the cross-linking of the stalk helices (source data are provided as a Source Data file). **c** Kymograph analysis of the antibody-dimerized motors with the buttress truncations reveals that neither ΔBUT1-Dyn1_331 kDa_ (left) nor ΔBUT-E3197K-Dyn1_331 kDa_ (right) move along MTs. Instead, both motors show non-motile MT interactions (increasing the ionic interaction strength between MTs and the MTBD via the E3197K mutation increases the lifetime of the non-motile binding events, see Supplementary Fig. 9b). **d** Kymograph analysis of the antibody-dimerized WT Dyn1_331 kDa_-β CL and WT Dyn1_331kDa_-γ CL motors reveals only non-motile MT interactions. **e** Preventing stalk-buttress interactions in the ΔBUT-Dyn1_331 kDa_-α CL motor results in non-motile interactions, demonstrating the importance of stalk-buttress interactions. The depicted scale bars are the same for all kymographs shown in this figure

Buttress truncation uncouples ATPase activity and MT binding in the absence of load^14^ and also prevents tension-induced transition from weak to strong MT binding (Figs. 2f and 5c), suggesting that the buttress is essential for motility. To test this prediction, we developed an in vitro approach to homodimerize (or heterodimerize) our single-headed dyneins using an antibody against the N-terminal GFP of the motors (see Methods). While antibody-dimerized WT Dyn1_331kDa_ is highly processive and as fast as GST-dimerized Dyn1_331kDa_^37^ (Fig. 7a), antibody-dimerized ΔBUT-Dyn1_331 kDa_ (i.e., with the distal part of the buttress deleted (ΔBUT, Fig. 5b)) only exhibits brief, non-motile MT interactions (as does ΔBUT-Dyn1_471 kDa_, a homodimeric full-length dynein bearing the same buttress truncation, see Supplementary Fig. 9a) as revealed by kymograph analysis (Fig. 7c, left).

In a recent study, Cleary et al. demonstrated that an active dynein head (capable of performing an ATP-dependent linker swing) fused to an inactive MT tether moves processively along MTs if the MT affinity of the tether is sufficiently high^33^. A dynein head fused to a MT tether consisting of the SRS-stalk-MTBD construct in the low-affinity β-registration was incapable of processive motion, while the high-affinity α-registration exhibited a two-fold higher processivity than the dimeric WT motor, albeit at a threefold lower speed than the WT motor^33^ (note that while this high affinity α-registration construct has a higher MT-binding strength than the SRS-β stalk-MTBD construct (Fig. 4b, d), the stalk registration of this construct is distinct from the true α-registration, which is only assumed when the stalk helices are cross-linked in the α-registration). Thus, a strongly bound leading head is capable of dragging an inactive, strongly MT-bound MTBD forward (at the cost of speed) by changing the linker conformation. As the previous work on *Dictyostelium* dynein has shown that a buttress truncation does not completely abolish ATP-dependent movements of the linker^14^, we wanted to test whether the weak MT-binding strength of the buttress-truncated dynein head (Fig. 5c) prevents antibody-dimerized ΔBUT-Dyn1_331kDa_ from moving processively. To test this, we increased the ionic interaction between the MTBD and MT by mutating the highly conserved glutamic acid E3197 in H6 of the MTBD to a basic amino acid (K) (Fig. 1c). This mutation significantly increases the processivity of *S. cerevisiae* dynein^30^, suggesting stronger MT binding by the MTBD^33^. However, while the point mutation increased the time the antibody-dimerized ΔBUT-E3197K-Dyn1_331kDa_ motors interacted with MTs (Fig. 7c and Supplementary Fig. 9b), the motors were still non-processive.

It is possible that the buttress deletion not only prevents registration changes of the stalk helices but that the absence of stalk-buttress interactions also causes conformational changes at the base of the buttress and possibly within AAA5 or the entire AAA ring that negatively impact dynein’s motion-generating linker conformational change. To test this possibility, we first determined whether dynein is capable of moving processively along MTs when its stalk helices are fixed in the weak, intermediate or strong MT-bound helix registrations with a buttress capable of interacting with the stalk. Intriguingly, while both the antibody-dimerized Dyn1_331 kDa_-β CL and Dyn1_331 kDa_-γ CL mutants show only brief, non-motile MT interactions (Fig. 7d), Dyn1_331kDa_-α CL exhibits highly processive motion albeit at a significantly lower speed than the antibody-dimerized Dyn1_331kDa_ motor (Fig. 7a, b). In contrast, in the presence of TCEP, all three mutant motors are as processive and fast as antibody-dimerized WT Dyn1_331kDa_ (Fig. 7b, bottom right and Supplementary Fig. 10a, b), demonstrating not only the reversibility of the cross-linking of the stalk helices but also that all three motors are as enzymatically active under non-cross-linked conditions as WT Dyn1_331kDa_. Antibody-dimerized WT Dyn1_331kDa_ on the other hand, shows the same motility behavior under cross-linking (Fig. 7a) or non-cross-linking conditions (Supplementary Fig. 10b). Together, these results suggest that the Dyn1_331kDa_-α CL motor is capable of generating a motility-driving linker conformational change even when its stalk helices are cross-linked via the inserted paired cysteines. In contrast, antibody-dimerized ΔBUT-Dyn1_331kDa_-α CL motors without functional buttress-stalk interactions only show brief, non-motile MT interactions (Fig. 7e). Our data suggest that the disruption of stalk-buttress interactions results in conformational changes within AAA5 that impair motion-generating conformational changes of the linker, possibly by preventing functional interactions between the linker and AAA5 during dynein’s post-powerstroke state.

## Discussion

While previous studies demonstrated that dynein’s MT binding strength responds anisotropically to tension^32–35^, the underlying mechanism was unclear. Here, by combining mutagenesis, structure-function and chemical cross-linking with single-molecule optical tweezers, we demonstrate that dynein’s anisotropic response to tension is not intrinsic to the MTBD alone, but is controlled by dynein’s AAA ring and its three appendages, the stalk, buttress, and linker. These elements work together to control the MT-binding strength of the MTBD in a tension direction-dependent manner.

In the absence of stalk-buttress interactions, MT binding is weak in both directions and indistinguishable from the MT-binding strength of a motor with the stalk helices cross-linked in the β-registration. This suggests that the stalk helices assume a β-registration in the relaxed state of the stalk coiled-coil, in agreement with biochemical and structural studies^14,27,28^. In contrast, backward load induces strong MT binding of the WT motor in the absence of nucleotides, similar to the MT-binding strength of a motor with the stalk helices cross-linked in the α-registration. Thus, backward tension applied to the MD induces the α-registration of the stalk.

Our data also reveal that forward tension alone is not sufficient to induce the β-registration in the WT MD in the absence of nucleotides and instead induces a stalk helix registration that is distinct from the known β- and α-registrations. This registration results in an intermediate MT-binding strength under forward load (Fig. 2e). As we showed previously^32^, the transition into dynein’s weak MT-binding state under linker-applied forward load occurs only if AAA1 and AAA3 are bound to ATP (when tension is applied via dynein’s C-terminus, AAA3 needs to be in the post-hydrolysis state^32,46^). This suggests that tension-induced transition into the β stalk registration requires nucleotide-induced structural changes in the AAA ring.

What stalk helix registration does dynein assume under forward load in the apo state if not the β-registration? The crystal structures solved in dynein’s post-powerstroke ADP state (*Dictyostelium* dynein 1)^14^ and pre-powerstroke ADP.Vi state (human dynein 2)^47^ suggest that dynein’s stalk helices transition between the α- and β-registrations. Locking the stalk helix registration in between the α- and β-registrations indeed results in MT-binding strengths under forward and backward load that are statistically indistinguishable from the MT-binding strength of the WT motor under forward load in the apo state (Figs. 2e and 3d). This intermediate stalk helix registration, which we term the γ-registration, is likely near the stalk registration assumed in dynein’s rear head when ADP is still bound to AAA1 as well as after ADP release: the mean unbinding forces under forward load are the same for WT dynein in the apo state and for the dynein AAA3 K/A mutant with ADP bound to AAA1 and AAA4, no nucleotide bound to AAA3, and ATP firmly bound to AAA2^13,32^. This finding suggests that dynein assumes at least three different stalk registrations during the mechanochemical stepping cycle and underlines the intricate nature of the pathway used to communicate bidirectionally between dynein’s AAA ring and its MTBD.

Our buttress truncation experiment demonstrates that functional stalk-buttress interactions are necessary to induce the α-registration of the stalk helices under backward load. Whether tension applied to the linker results in a pulling or pushing of the buttress on the stalk helices remains to be shown. However, since the absence of functional buttress-stalk interactions results in the weak MT binding β-registration independent of the direction of tension, it is clear that the tension-induced transition into the α-registration requires a buttress-induced change from the β- to the α-registration. While dynamic changes in the registry of the stalk helices of dynein could occur so that different registries are dynamically sampled, with one registry favored over the others, our data suggest that the buttress together with applied tension highly stabilizes distinct registries depending on the direction of applied tension and nucleotide state.

The dynein mutant F3446E-Dyn1_331 kDa_ shows MT-binding strengths statistically indistinguishable from the motor with the stalk helices cross-linked in the β-registration. Thus, linker-AAA5 docking is required for the tension-induced α-registration under backward load and the γ-registration under forward load, while the undocking of the linker from AAA5 is required for the transition into the β-registration. Since the buttress truncation mutant and the cross-linked β-registration mutant have statistically indistinguishable MT-binding strengths, linker docking appears to control the conformational changes of the buttress. A tension-induced, strong MT-binding α-registration with the linker docked to AAA5 in its post-powerstroke conformation is consistent with the load-bearing requirements of the front head in the post-powerstroke state. In contrast, ATP-induced undocking of the linker from AAA5 would facilitate the detachment of dynein’s rear head via a transition into the weak-MT binding β-registration under forward-directed (intermolecular) tension. Thus, ATP binding to AAA1 accelerates MT detachment via a transition into the β-registration initiated by the undocking of the linker.

We note that the requirement of AAA5-linker interactions for the tension-induced α-registration appears to conflict with the α-registration observed in the crystal structure of *Dictyostelium* dynein solved in the presence of ADP (i.e., in the presumed post-powerstroke state). In the crystal structure, the linker lies close to AAA4 rather than AAA5^14^. It is possible that the linker and/or stalk conformation is different when dynein is bound to MTs in the presence of ADP or that the linker and/or stalk helices assume different conformations in the crystal structure versus in solution. Indeed, we previously showed that ADP binding to AAA3 induces a weak MT-binding state of dynein under load^32^, which is inconsistent with the α-registration in the dynein crystal soaked with ADP (i.e., with an ADP molecule bound to AAA3). Furthermore, a recent cryoEM study has demonstrated that with ADP bound to AAA3, dynein assumes the low-affinity stalk registration in its auto-inhibited conformation known as the phi-particle^48^. Thus, it is possible that the interactions between the MDs and/or the linkers as seen in the phi-particle have similar effects on the stalk helix registration as linker-applied tension. Solving the crystal structure of dynein bearing an AAA3 K/A mutation in the presence of ADP could help to resolve these apparent discrepancies.

Previous work has shown that the prevention of buttress-stalk interactions uncouples dynein’s ATPase activity from MT binding and results in weak MT-binding^14,31^. However, how this behavior affects the motion of two-headed dynein was undetermined. Here, we show that the two-headed motor with a buttress truncation is immotile, even if the MT-binding strength is increased with a point mutation in the MTBD. Since a single dynein head capable of a power stroke is able to move processively when linked to a strong MT-binding tether^33^, we would have expected that our two-headed dynein motor with the buttress truncation and the increased MT-binding strength would be capable of processive motion—as long as the MD could perform a power stroke. Previous FRET studies on *Dictyostelium* dynein demonstrated that linker movements still occur in the absence of stalk-buttress interactions^14^, suggesting that dynein may still be capable of a power stroke without the buttress. However, our observations suggest that dynein is unable to generate a linker conformational change capable of powering unidirectional motion in the absence of buttress-stalk interactions. Our observation that an antibody-dimerized dynein with the stalk helices cross-linked in the α-registration is capable of generating processive motion (owing to its residual anisotropic MT-binding strength, Fig. 3b) only if the buttress can interact with the stalk further supports the conclusion that functional buttress-stalk interactions are not only required for the communication between the AAA ring and MTBD but also for an ATP-induced, motion-generating power stroke of the linker. As our data reveal that linker interactions with AAA5 control the tension-induced and buttress-dependent registry changes of the stalk helices, it is possible that a lack of stalk-buttress interactions alters the base of the buttress and possibly the conformation of AAA5, thereby preventing functional interactions between the linker and AAA5. This could result in the weak MT-binding β-registration in dynein’s post-powerstroke state and possible impairment of the post-powerstroke conformation of the linker.

Our findings support a revision of the current consensus model for dynein motion. In the current model, ATP binding to AAA1 of dynein’s rear head causes head dissociation from the MT, followed by the priming stroke of the linker in the detached head^3,45,49^. Our data suggest that rear head detachment occurs after the ATP-induced undocking of the linker from AAA5, an event required for the transition into the weak MT-binding β-registration. Thus, the priming stroke likely already starts while the rear head is still bound to the MT, rather than after MT dissociation. In addition, our data reveal that linker docking to AAA5 is required for the transition into the α-registration in dynein’s front head. Thus, in contrast to models that postulate that dynein’s front head assumes a strong MT-binding state before the power stroke occurs^49^, our data suggest that the power stroke induces the strong MT-binding state of the leading head.

**Fig. 8:**
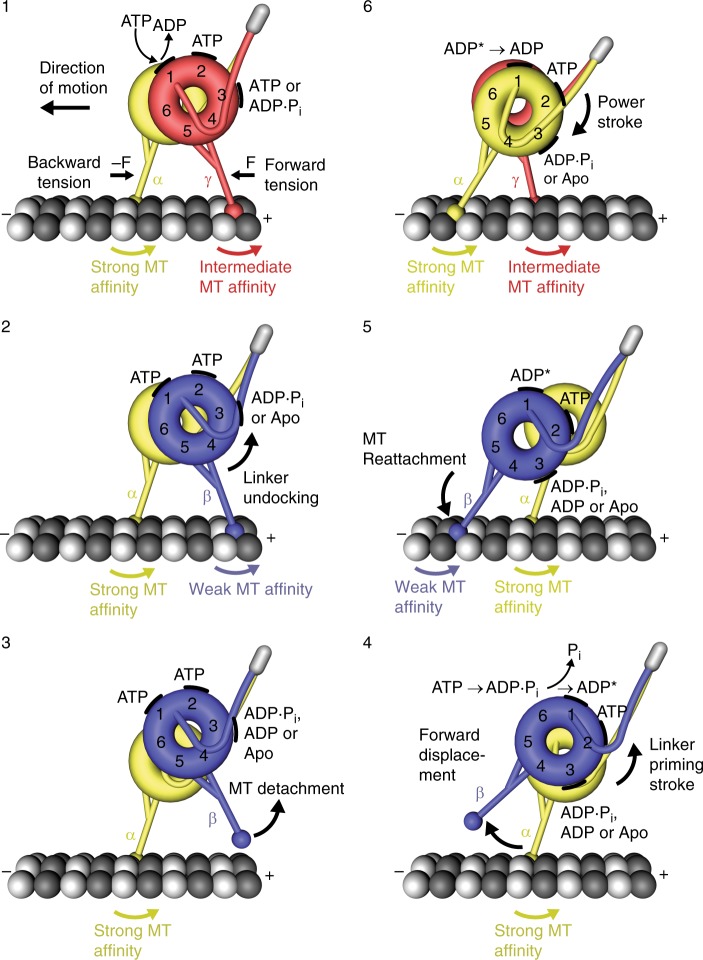
Model for the mechanochemical cycle of dynein. Following ADP release from AAA1, ATP binding (step 1) causes the undocking of the linker from AAA5 and the subsequent transition from the γ-registry of the stalk helices with intermediate weak MT-affinity to the weakly MT-binding β-registry (step 2). After the detachment of the rear head (step 3), which occurs when AAA3 is in the ADP∙Pi transition state or bound to ADP, the ‘recocking’ of the linker (priming stroke) displaces the detached head forward to a new front MT-binding site while the MT-attached head bears the load (step 4). Following ATP hydrolysis and Pi release from AAA1 (step 4), rebinding to the MT in the weakly MT-binding β-registry (step 5) causes the transition from the ‘high-energy ADP* state’ to the ‘low-energy ADP state’, which generates a linker swing (powerstroke), resulting in the docking of the linker to AAA5 and the transition into the strong MT-binding α-registry of the stalk helices (step 6). A prerequisite for the strong binding state is that AAA3 is not bound to ADP, suggesting that AAA3 is still in the ADP∙Pi state or nucleotide free. The MT minus-end-directed linker swing generates the forward movement of dynein’s center of mass and the attached load

On the basis of our findings here and in previous work^32^, we propose the following model for dynein’s mechanochemical cycle, taking into account the nucleotide states of AAA1 and AAA3 (Fig. 8). Because AAA2 likely remains bound to ATP throughout the mechanochemical cycle^13,14^, it is unlikely to play an active regulatory role, and while AAA4 likely has a regulatory function^50,51^, the significance of its nucleotide state in the overall mechanochemical cycle is undetermined. We start with ATP binding to AAA1 of dynein’s trailing head (Fig. 8, step 1), which causes linker undocking from AAA5 and the subsequent transition into the weak MT-binding β-registration (Fig. 8, step 2). After detachment from the MT (Fig. 8, step 3), the free head undergoes a forward movement driven by the priming stroke of its linker (Fig. 8, step 4). The priming stroke occurs while ATP is bound to AAA1 and with AAA3 likely in the ADP.Pi transition state or bound to ADP, since ATP binding to AAA1 induces MT release only if AAA3 is in the post-hydrolysis state^32,46^ (if the linker is under tension, ATP binding to AAA3 is enough to open the gate^32^).

Following ATP hydrolysis, the tethered head then binds to a new binding site on the MT (Fig. 8, step 5). It is under debate whether phosphate releases before MT binding or whether MT binding stimulates phosphate release^14,24^. On the basis of our data, initial binding is likely via a weak interaction. MT binding then causes the transition from the ‘high-energy ADP state’ to the ‘low-energy ADP state’^24,39,52^, which is accompanied by the power stroke^22^ (Fig. 8, step 6). Binding of the linker N-terminus to AAA5 in the post-powerstroke state then allows the tension-induced transition into the strong MT-binding α-registration, a state capable of bearing load. We note that the crystal structure solved in the presence of ADP suggests that the force-bearing head in its post-powerstroke state assumes the α-registration^14^. However, our previous data show that ADP binding to AAA3 induces a weak MT-binding state under backward load^32^, suggesting that AAA3 may not contain ADP following the powerstroke. Recent work by Dewitt et al. suggests that AAA3 hydrolyzes ATP an order of magnitude slower than AAA1^46^ under unloaded conditions, suggesting that AAA3 activity is not synchronized with the activity of AAA1. In addition, we previously showed that MT-binding strength is stronger under backward load when ADP is bound to AAA1^32^. We therefore suggest that in the post-powerstroke state of the force-bearing leading head, AAA1 is bound to ADP (‘low-energy ADP state’) and AAA3 is in the ADP.Pi transition state. However, it remains to be shown whether a dynein head with ADP bound to AAA1 and AAA3 in the ADP.Pi transition state binds MTs strongly enough under backward load to remain attached. In addition, it is possible that AAA3 activity is synchronized with the activity of AAA1 under load, in which case AAA3 may assume a nucleotide-free state, which would result in strong MT binding under backward load when AAA1 is bound to ADP^32^. Thus, AAA3 may be ADP bound only at appropriate points in the cycle, such as when the head is detached from the MT or when ATP binds to the rear head AAA1, thereby assisting in MT release^32^.

It is possible that the power stroke of the MT-bound leading head increases intramolecular tension if the MT-bound rear head still has ADP bound to AAA1, or if ADP is released so that AAA1 assumes the apo state. This transition could induce the γ-registration, leading to an intermediate MT-binding strength, or directly contribute to forward movement if ATP binds AAA1 in time to induce rear head detachment. The latter event, which only occurs when ATP is bound to AAA3 (if the linker is under tension^32^) or when AAA3 is in the post-hydrolysis state (ADP.Pi or ADP)^46^, allows the rear head to transition into the weak MT-binding state by assuming the β-registration. With the ATP-induced rear head detachment, a new mechanochemical cycle begins.

In summary, our findings demonstrate the complex nature of dynein’s force- and motion-generating mechanism, in which dynein’s AAA+ MD and its three appendages, the stalk, buttress, and linker, work together to regulate the cyclic weak and strong MT interactions of the MTBDs. This regulation ensures that one MD holds tightly to the MT while the other detaches and advances. In the case of mammalian dynein, this mechanism may ensure processive dynein stepping following its activation by its largest cofactor, dynactin, which, together with a coiled-coil containing cargo adaptor (such as BICD2), converts mammalian dynein from a diffusive^53^/weakly processive^54^ motor to an ultraprocessive motor^55,56^, possibly by reorienting dynein’s MDs^48,57,58^. Future studies will be needed to determine which orientations and stalk registrations dynein’s MDs assume when complexed with dynactin and how teams of dynein motors are coordinated when recruited to dynactin via the cargo adaptors BICDR1 and HOOK3, which have been reported to predominately bind two dynein motors^59,60^. Finally, insights are needed into whether the activities of AAA1 and AAA3 are coordinated under load.

## Methods

### Generation of yeast strains

Mutant yeast strains (listed in Supplementary Table 1) were generated using two-step selection methods (SC/URA- and 5-FOA)^61^. PCR primers were designed using the PrimerQuest tool from Integrated DNA Technologies, and the PCR protocols for KOD Hot Start DNA polymerase (EMD Millipore) were followed (see Supplementary Table 2 for the list of primers used) to generate DNA fragments. Yeast transformation was performed using either the standard yeast transformation method (based on the LiAc/ss carrier DNA/PEG protocol^62^) or the Frozen-EZ Yeast Transformation II kit from Zymo Research, with SC/URA- (synthetic media with uracil-dropout amino acid mix) and SC/5-FOA (5-fluorouracil) as selective agents. All newly engineered and mutated yeast strains were confirmed by PCR and sequencing.

### Generation of plasmids for SRS-stalk-MTBD constructs

Standard molecular cloning methods were used to create chimeric constructs of monomeric *Thermus thermophilus* seryl-tRNA synthetase (SRS) fused to the stalk and MTBD of yeast dynein using SRS85:82, a construct with the near full-length mouse stalk and MTBD fused to the coiled-coil base of SRS in the α-registry^27,33^ as a template. Genomic yeast DNA was amplified to generate DNA fragments of the dynein stalk and MTBD. PCR products were then stitched with partial SRS sequences to enable insertion between the restriction enzyme sites, SalI and PstI, of the original vector (see Supplementary Table 2 for the list of primers used). Single point mutations in the SRS chimeras were generated using the Q5 site-directed mutagenesis kit from NEB. High efficiency 5-α competent *E. coli* cells (NEB) were used for transformation and plasmid amplification. Single colonies were inoculated in 3 mL of terrific broth (TB) with 30 μg/mL of kanamycin and grown overnight shaking at 37 °C. Plasmids (listed in Supplementary Table 1) were purified using the PureYield plasmid miniprep kit from Promega and verified by standard sequencing.

### Yeast growth and yeast dynein expression

Single-headed dynein constructs (based on tail-truncated dynein, Dyn1_331kDa_) were expressed behind the inducible galactose promoter (GAL1-GAL10), while two-headed full-length dynein constructs (based on Dyn1_471kDa_) were expressed behind the native promoter^61^. Yeast growth was done at 30 °C with shaking. For single-headed dynein, a single yeast colony was inoculated in 5 mL of 2 × YPD (20 g/L yeast extract, 40 g/L peptone, 4% (w/v) dextrose) overnight. Pre-cultures were then inoculated in 50 mL of YPR (2% (w/v) raffinose) for 8 h. Expression of dynein was induced by transferring the YPR culture into 2 L of 2 × YPG (4% (w/v) galactose). Cultures were then grown to a final OD_600_ between 1.5 and 2.5 (~16 h). To express full-length dynein, yeast cells were grown similarly to the growth for the expression of single-headed dynein, except that only 2 × YPD was used throughout the growth, and cells were grown to a final OD_600_ of ~0.8. Cells were then harvested via centrifugation at 1000 × g for 3 min, and washed once with ddH_2_O. Finally, pellets were resuspended in 0.2 volumes ddH_2_O and flash-frozen in liquid nitrogen as small droplets, and stored in 50-mL conical tubes at −80 °C until further use.

### Expression of SRS-stalk-MTBD constructs

For the expression of the SRS chimeras, plasmids encoding the SRS chimeric constructs were transformed into Rosetta (DE3) pLysS competent *E. coli* cells (Novagen) for protein expression. *E. coli* growth was done in the presence of 15 μg/mL kanamycin and 17 μg/mL chloramphenicol at 37 °C with shaking unless specified otherwise. For each construct, a single colony was inoculated in 3 mL terrific broth overnight and then diluted into 100 mL terrific broth at a starting OD_595_ of ~0.1 to create a pre-culture. Following a ~2-hour growth until OD_595_ reached ~0.6–1, the pre-culture was added to 900 mL terrific broth. The full culture was grown at 37 °C until OD_595_ reached ~0.8 and then cooled on ice below 18 °C. Protein expression was induced by addition of 0.1 mM IPTG (isopropyl β-D-1-thiogalactopyranoside), followed by incubation at 18 °C with shaking for 16 h. The cells were then pelleted at 3000 × *g* for 5 min, the supernatant was discarded and the residual liquid was used to resuspend the cells. The cell slurry was then transferred to a 50-mL conical tube and stored at −80 °C until further use.

### Yeast dynein purification

All dynein constructs have an N-terminal ZZ-tag (synthetic two-domain IgG-binding sites based on staphylococcal protein A^63^) for binding to beads coated with IgG (immunoglobulin G) during affinity purification, followed by an enhanced TEV (tobacco etch virus) protease cleavage sequence, a GFP (yEGFP3, a yeast-codon optimized enhanced green fluorescence protein, GFPmut3^64^), and a hemagglutinin (HA) tag (not employed in this work). The construct for full-length dynein, Dyn1_471kDa_, has an additional Halo-Tag between the GFP and HA tags. The SNAP-tagged single-head dyneins based on Dyn1_331kDa_ have an additional SNAPf-tag between the GFP and HA tags. Protein purification was done at 4 °C unless specified otherwise. For each purification, the stored frozen cell droplets were pulverized using a kitchen coffee grinder pre-chilled using liquid nitrogen, followed by addition of 0.25 volumes of 4× lysis buffer (1× lysis buffer: 30 mM HEPES, 50 mM KAc, 2 mM Mg(Ac)_2_, 1 mM EGTA, 10% glycerol, 1 mM DTT, 0.1 mM Mg-ATP, 0.5 mM Pefabloc, 10 ng/mL Leupeptin, 10 ng/mL Pepstatin A, 0.2% v/v Triton X-100, pH 7.2) to reach a final concentration of 1 × lysis buffer. The cell lysate was then cleared via ultracentrifugation at 290,000 × *g* for 30 min. Next, 250 μL of IgG sepharose 6 fast flow beads (GE Healthcare) was added to the supernatant. The solution was then incubated for 1 hr while rotating. The dynein-bound IgG beads were then washed with 20 mL of 1× lysis buffer. To fluorescently label native dynein (or single-head dynein) with TMR (tetramethylrhodamine), TMR-conjugated Halo-tag ligand (Promega) (or SNAP-Cell® TMR-star ligand (NEB)) was added to the beads to a final concentration of 10 μM, and incubated with the beads at room temperature for 10 min. Afterwards, the beads were washed with 10 mL of 1× TEV protease cleavage buffer (30 mM HEPES, 150 mM KAc, 2 mM Mg(Ac)_2_, 1 mM EGTA, 10% glycerol, 0.1 mM Mg-ATP, 0.5 mM Pefabloc, 0.1% v/v Triton X-100, pH 7.2). The beads were then resuspended in an equal volume of the cleavage buffer and 40 units of AcTEV protease (ThermoFisher Scientific) was added. The mixture was nutated for 2 h, resulting in cleavage of dynein from IgG beads. Beads were then sedimented by centrifugation, and the dynein-containing supernatant was flash-frozen in 50 μL aliquots in liquid nitrogen (TEV-released dynein). The aliquots were finally stored at −80 °C until further usage.

### Microtubule binding and release purification of dynein

To further purify dynein and to remove dynein aggregates, we performed a MT binding and release assay for each dynein construct that was responsive to ATP. To 50 μL of TEV-released dynein, 10 μL of 5 mg/mL paclitaxel-stabilized MTs were added in the presence of 20 μM paclitaxel (Sigma). The solution was then layered onto a 100 μL sucrose cushion (30 mM HEPES, 200 mM KCl, 2 mM MgCl_2_, 10% glycerol, 25% w/v sucrose, 20 μM paclitaxel, pH 7.4) and centrifuged at 25 °C for 10 min at 60,000 × *g*. After the supernatant and cushion were gently removed without disturbing the pellet, the MT pellet was gently rinsed with 100 μL wash buffer (30 mM HEPES, 150 mM KCl, 2 mM MgCl_2_, 10% glycerol, pH 7.2; EGTA was not added due to its interference with oxidization by copper phenanthroline) and resuspended in 52 μL of wash buffer with 6 mM Mg-ATP. The solution was then centrifuged again for 5 min at 60,000 × *g*. Finally, the dynein-containing supernatant was aliquoted into 2 μL volumes, flash frozen in liquid nitrogen and stored at −80 °C until further use. For constructs that are insensitive to ATP, 2 μL of 5 mg/mL paclitaxel-stabilized MTs were added to 50 μL of TEV-released dynein in the presence of 20 μM paclitaxel and 6 mM Mg-ATP, and the sample was centrifuged for 5 min at 60,000 × *g* at room temperature to remove dynein aggregates.

### Purification of SRS constructs

An *E. coli* cell pellet was thawed at room temperature and an equal amount (1 mL per 1 g of cell wet weight) of 2 × lysis buffer (1 × lysis buffer: 50 mM Tris, 200 mM NaCl, 1 mM EGTA, 10% glycerol, 1 mM PMSF, 1 mM β-ME (β-mercaptoethanol), pH 7.6; β-ME was not added for the SRS mutants with the paired cysteines in the stalk helices) was added. After resuspension of the pellet, the cells were lysed by sonication at 4 °C. The lysate was then cleared by centrifugation at 400,000 × *g* for 10 min. 200 μL Ni-NTA resin (Qiagen) was added to the cleared lysate and imidazole (2 M, pH 8) was added to a final concentration of 10 mM. The solution was nutated for 1 h at 4 °C. The Ni-NTA resin was then washed with 10 mL of wash buffer (the same as 1× lysis buffer but with 20 mM imidazole), and the protein was eluted from the Ni-NTA resin with elution buffer (the same as 1 × lysis buffer but with 250 mM imidazole and without PMSF). The protein solution was aliquoted in 50 μL volumes and flash-frozen in liquid nitrogen. To remove imidazole, one aliquot of the eluted protein was exchanged into storage buffer (the same as elution buffer but without imidazole) using a Zeba™ Spin Desalting Column (ThermoFisher Scientific). The flow-through was aliquoted into 2 μL volumes. Finally, the aliquots were stored at −80 °C until further use.

### Preparation of coverslips for MT immobilization

Using forceps, coverslips (18 × 18 × 0.170 mm, Zeiss) were placed into a porcelain coverslip rack, submerged in HNO_3_ (25% v/v) for 10 min, rinsed with ddH_2_O, and then submerged in NaOH (2 M) for 2 min, followed by extensive rinsing with ddH_2_O. The coverslip rack was placed onto a heating block set at 90 °C to air-dry the cleaned coverslips for 10–20 min and then stored in a vacuum desiccator. The slide chamber was assembled as described previously^65^. For MT immobilization, 10 μL of 5 mg/ml biotinylated α-casein was flown into the slide chamber and incubated for 10 min. Then 3 × 20 μL of blocking buffer (80 mM PIPES, 2 mM MgCl_2_, 1 mM EGTA, 1% Pluronic F-127, 1 mg/ml α-casein, 20 μM Taxol) was used to wash the chamber, and incubated for 1 h to fully block the glass surface. Following the blocking step, the solution inside the chamber was completely removed, and 12 μL of 1 mg/ml streptavidin was flown into the chamber and incubated for 10 min. The chamber was then washed with 3 × 20 μL blocking buffer.

### Polymerization of polarity-marked MTs

The direction of unbinding forces was confirmed using polarity-marked MTs with bright, densely fluorescently labeled minus ends. To prepare polarity-marked MTs, 0.5 μL of 2 mg/mL TMR-labeled tubulin and 0.5 μL of 1 mg/mL biotin-labeled tubulin (Cytoskeleton) with 1 mM Mg-GTP were combined and incubated at 37 °C for 5 min to generate bright MT seeds. Next, 5 μL of 1 mg/mL unlabeled tubulin, 0.5 μL of 0.1 mg/mL TMR-labeled tubulin, and 0.5 μL of 0.1 mg/mL biotin-labeled tubulin with 1 mM Mg-GTP was added to the seeds. The mixture was incubated at 37 °C for 15 min. 0.7 μL of 0.2 mM paclitaxel in DMSO was then added to the mixture, and free tubulin was removed by centrifuging through a 60 μL glycerol cushion (80 mM PIPES, 2 mM MgCl_2_, 1 mM EGTA, 60% glycerol, 20 μM paclitaxel, pH 6.8) at 250,000 × *g* for 10 min. The cushion was then removed, and the pellet was washed with 2 × 20 μL wash buffer (80 mM PIPES, 2 mM MgCl_2_, 1 mM EGTA, 10% glycerol, 20 μM paclitaxel, pH 6.8), and resuspended in 20 μL wash buffer. The polymerized MTs were stored in the dark at room temperature.

### Constant-pulling assay

Unbinding force measurements were performed as previously described^32^ with the modifications indicated here. Briefly, the optical trap was calibrated via power spectrum analysis^66^ and the trap stiffness (*k*) was adjusted between ~0.025 and 0.06 pN/nm, depending on whether a weak MT-binding or strong MT-binding construct was studied. The speed of the nano-positioning stage, which varied between ~90 and ~225 nm/s depending on which trap stiffness was used, was adjusted to produce a theoretical loading rate of 5.6 pN/s once the motor bound to the MT (the actual loading rate that the MTBD-MT bond is exposed to is somewhat smaller than this value and force dependent due to the compliance of the motor construct, see Fig. 2g). Trapping buffer (TB: 30 mM HEPES, 2 mM MgAc_2_, 1 mM EGTA, 20 μM paclitaxel, 20 mM glucose, 2 mM Trolox, pH 7.2) was used for all experiments unless specified otherwise. 20 μL of 0.01 mg/mL polymerized MTs was flown into the prepared flow chamber described above and flushed immediately with 2 × 20 μL TB. The oxidization reagent, copper phenanthroline (Cu(PT)_3_), was prepared fresh before each experiment by mixing equal volumes of 60 mM CuSO_4_ and 180 mM phenanthroline. Dynein (or SRS-stalk-MTBD) constructs were diluted stepwise in TB containing 0.75 mg/mL α-casein. Next, a 4 μL solution containing anti-GFP antibody Fab fragment-coated, ~1-μm diameter beads (870 nm, carboxyl-modified polystyrene microspheres, Bangs Laboratories) (prepared as described previously^66^, except that α-casein was used in place of BSA, and anti-GFP antibody Fab fragments were used instead of anti-GFP antibodies; the Fab fragment was prepared following the vendor’s protocol) was incubated with appropriate concentrations of diluted dynein (or SRS-stalk-MTBD) to produce MT binding by <50% of the probed beads in the final assay to ensure trapping experiments were performed at the single-molecule level^32^ (the required dilution was in all cases (t100). The motor-bead solution was then incubated on ice for 10 min. To remove free unbound motors, beads were centrifuged for 2 min at 3000 rcf at 4 °C, followed by the removal of the supernatant. To crosslink the paired cysteines in the stalk, 20 μL oxidization buffer (30 mM HEPES with 300 μM Cu(PT)_3_, pH 7.2) was used to resuspend the beads, followed by an incubation of the solution on ice for 10 min. A concentration of 20 μL TB with 0.75 mg/mL α-casein was then added to the solution and the centrifugation was repeated. After the supernatant was removed, 40 μL TB with 0.75 mg/ml α-casein and Gloxy (a glucose oxidase and catalase-based oxygen scavenging system^67^) was used to resuspend the beads. The mixture was then introduced into the flow chamber, and the flow chamber was sealed with vacuum grease. As all dynein constructs are pre-diluted at least 100 times, followed by a resuspension of the motor-bound beads with 40 μL buffer twice, the nucleotide-free apo experiments reported herein were performed in the presence of a residual ATP concentration of 37.5 nM (the initial ATP concentration is 6 mM after the MT-binding and -release assay described above). For the experiments with TCEP (tris(2-carboxyethyl)phosphine) (reducing conditions), TCEP was added to a final concentration of 1 mM and incubated with the dynein (or SRS-stalk-MTBD) construct on ice for 10 min before the construct was diluted. TCEP was added to the final resuspension solution to 0.2 mM.

### Oscillatory trap assay

The measurement of the force-dependent unbinding rates of the mouse SRS-α stalk-MTBD construct using the oscillatory assay (Supplementary Figs. 5–7) was done as previously described by Cleary and coworkers^33^. For detailed protocols, see Supplementary Note 3.

### Single-molecule fluorescence motility assay

TB with 75 mM KAc (TBK75) was used for all single-molecule motility experiments. 20 μL of 0.01 mg/ml Cy5-labeled MTs^65^ was flown into the slide chamber as described above, and flushed immediately with 2 × 20 μL TBK75. To image the antibody-dimerized motors in the TIRF assay, 100 μg anti-GFP antibody was labeled with 20 μM Cy3-NHS at room temperature for 1 h, followed by the removal of the free dyes using an Amicon® Centrifugal Filter Unit. To oxidize the paired cysteines in the stalk, dynein constructs were diluted appropriately in a dilution buffer (30 mM HEPES, 2 mM MgCl_2_, 150 mM KCl, pH 7.2), followed by the addition of an equal volume of 600 μM Cu(PT)_3_ in dilution buffer. The solution was then incubated on ice for 10 min, after which EDTA was added to final concentration of 5 mM and incubated on ice for 5 min to quench Cu(PT)_3_. To reduce the disulfide bond that cross-linked the stalk helices, dynein was diluted appropriately in dilution buffer and an equal volume of 2 mM TCEP in dilution buffer was added to the motor. The solution was then incubated on ice for 10 min. Once the motor was either oxidized or reduced, Cy3-labeled anti-GFP antibody was added to the motor solution to a final concentration of 50 μg/mL, and incubated on ice for 10 min. A final TBK75 solution containing 1 mM ATP, 1 mg/mL α-casein, 2 mM Trolox, Gloxy, 1 mM TCEP (in case of reducing experiments) and 0.5 μL of the motor-antibody mixture was flown into the chamber, which was then sealed with vacuum grease. For the full-length homodimeric WT dynein, Dyn1_471 KDa_, the final TBK75 buffer containing the motor was directly flown into the chamber after the MTs were immobilized on the glass surface. Experiments were performed with a custom-built total TIRF microscope equipped with an Andor iXon Ultra EMCCD. The acquisition time was set to 500 ms/frame (if not specified otherwise), and a total 600 images was acquired for each movie. The kymographs were generated using Fiji.

### Single-molecule dwelling time measurements

a concentration of 20 μL of 0.01 mg/ml Cy5-labeled MTs^65^ was flown into the slide chamber as described above, and flushed immediately with 2 × 20 μL TB. To oxidize the paired cysteines in the stalk, dynein constructs were diluted appropriately in a dilution buffer (30 mM HEPES, 2 mM MgCl_2_, pH 7.2), followed by the addition of an equal volume of 600 μM Cu(PT)_3_ in dilution buffer. The solution was then incubated on ice for 10 min, after which EDTA was added to final concentration of 5 mM and incubated on ice for 5 min to quench Cu(PT)_3_. To reduce the disulfide bond that cross-linked the stalk helices, dynein was diluted appropriately in dilution buffer and an equal volume of 2 mM TCEP in dilution buffer was added to the motor. The solution was then incubated on ice for 10 min. Once the motor was either oxidized or reduced, a final TB solution containing 1 mg/mL α-casein, 2 U/ml apyrase (apo state) or 1 mM ATP (ATP state), 2 mM Trolox, Gloxy, 1 mM TCEP (in case of reducing conditions) and 0.5 μL of the motor was flown into the chamber, which was then sealed with vacuum grease. Experiments were performed with the custom-built TIRF microscope described above. The acquisition time was set to 100 ms per frame, and a total of 1000 images was acquired for each movie. Image sequences were analyzed using a custom-written MATLAB kymograph program described previously^68^. GraphPad Prism was then used to fit the experimental CDFs of the measured dwell times to a theoretical CDF derived from an exponential decay function, yielding the unbinding rate *k* (Supplementary Fig. 2).

### Analysis of data generated by the constant-pulling assay

As we showed previously^32^, the largest forces in our unbinding experiments using the constant-pulling assay (Fig. 2b) usually occur when the bead rebinds the MT before returning to the trap center (Fig. 2d). We call these secondary binding/unbinding events. For primary events, zero force is applied to the MD immediately after MT binding (F_start_ = 0), while for secondary events, F_start_ > 0. As the history of force applied to the bond depends on F_start_, we focus only on primary events (Fig. 2d). Unbinding forces (example traces for all constructs studied are shown in Supplementary Figs. 11–27) were then analyzed using a semi-automated detection program written in MATLAB as previously described^32^. Measurements from multiple beads and experiments under the same conditions were pooled together and used to generate unbinding force histograms with 1-pN bins (as our force detection limit is ~0.3 pN, we only plot the force-dependent unbinding rates for forces above 0.5 pN). To facilitate comparison of the unbinding-force distributions and the derived force-dependent unbinding rates for both loading directions, we plot the data as a function of the absolute force values. Normalized histograms, approximating the probability density functions for unbinding at a given force, were then calculated by dividing the value of each bin by *N*, the total number of unbinding force measurements. Because the unbinding force distributions were not normally distributed, we estimated the sampling error by bootstrapping rather than calculating the standard error of the mean. For each histogram, 95% confidence intervals (CIs) for the mean statistic were calculated using the MATLAB bootci() function as described before^32^. To compare the measured histograms of primary unbinding forces, we calculate the empirical (Kaplan-Meier) cumulative probability distribution functions using the MATLAB function ecdf() and perform a two-sample Kolmogorov–Smirnov (KS) test (yielding a *p-*value *p*_ks_). To estimate *p*-values when comparing means of different distributions, we first created a dataset representing the sampling distribution of the mean for each original dataset, by bootstrapping 10^5^ means with the MATLAB function bootstrp(). We then subtracted these means pairwise to create a dataset representing the sampling distribution of the difference of the means. From each measurement in this dataset, we subtracted the mean difference of means, so as to shift the distribution to a mean of zero, consistent with the null hypothesis of no difference between the means of the original unbinding force distributions. The *p-*value (*p*_m_) was then calculated as the proportion of the bootstrapped mean differences that were at least as great as difference observed between the means of the original datasets (two tailed test). If no bootstrapped mean differences met this criterion, *p*_m_ is reported as <10^−5^.

Unlike our previous work, which assumed a constant loading rate in our unbinding-force assay^32^, here we considered the compliance of the dynein motor and bead linkage, resulting in a force-dependent loading rate (Fig. 2g and Supplementary Fig. 3). Uncertainty arises in the calculated unbinding rates as a function of force due to limited statistics for larger forces. To reduce this uncertainty, we used a kernel density estimator^69^ to describe the probability density functions of the measured unbinding forces before transforming them into force-dependent unbinding rates^70^ (see Supplementary Note 6 and Supplementary Figs. 28–31). While our new analysis confirms our previous conclusions, the absolute values of the calculated unbinding rates are slightly reduced compared to our previous results due to the motor’s force-dependent compliance.

### Reporting Summary

Further information on research design is available in the Nature Research Reporting Summary linked to this article.

## Supplementary information

Supplementary Information

Reporting Summary

Source Data

## Data Availability

Data supporting the findings of this manuscript are available from the corresponding author upon reasonable request. A reporting summary for this Article is available as a Supplementary Information file. The datasets underlying Figs. 2e, 3b–g, 4a–e, 5c, 6b, c and 7a, b and Supplementary Figs. 2, 3, 4a, b and 6, 9b and 10 are provided as a Source Data file.
