# Supplementary material for: Molecular mechanism of cytoplasmic dynein tension sensing
Source: Nat Commun. 2019 Jul 26;10:3332. doi: 10.1038/s41467-019-11231-8 (PMC6659695; doi:10.1038/s41467-019-11231-8)
Supplement: Supplementary file 1 — Supplementary Information [file 41467_2019_11231_MOESM1_ESM.pdf]

“Molecular mechanism of cytoplasmic dynein tension sensing”

Rao et al.

Supplementary Information

Supplementary Notes

Supplementary Note 1

Structural rendering. All images showing dynein structures were rendered using Chimera (UCSF). To construct the dynein motor domain bound to α/β-tubulin in its strong binding state (Fig. 1b), the structure of the *D. discoideum* dynein motor domain (PDB entry 3VKG) was combined with the mouse MTBD with the short stalk bound to bovine α/β-tubulin in the strong binding state (PDB entry 3J1T) using a sequence alignment tool. To construct the dynein motor domain bound to α/β-tubulin in its weak binding state, the structure of the mouse MTBD with stalk in the weak binding state (PDB entry 3WUQ) and the structure of the human dynein-2 motor domain (PDB entry 4RH7) were aligned and combined. The created dynein structures were then used to generate the morphing conformations shown in Fig. 3a and Supplemental Fig. 1b.

Supplementary Note 2

Cross-linking efficiency of the stalk helices. The cross-linking efficiency of the stalk helices of the dynein and SRS α-, β- and γ-mutants was determined by a combination of optical trapping measurements and zero-load motility experiments using the antibody-dimerized cross-linked motors. The cross-linking efficiency of the β- and γ-mutants can be directly determined from the performed unbinding-force experiments: under non-reducing conditions to promote cross-linking of the stalk helices, only 2 out of 40 beads coated with the β-mutant and 1 out of 26 beads coated with the γ-mutant exhibited the anisotropic unbinding behavior as seen for the WT motor (Figs. 2e-f and 3c,d) (at a bead fraction of less than 50% to ensure measurements at the single-molecule level[1]), suggesting that the cross-linking efficiencies for the β- and γ-mutants are close to 95%.

To estimate the cross-linking efficiency of the α-mutant, which shows large unbinding forces in both directions but with an overall remaining asymmetry (Fig. 3b), we performed zero-load motility experiments in the absence and presence of 2 mM TCEP (which cleaves the disulfide bonds of the cross-linked stalk helices). In the absence of TECP under non-reducing conditions following the cross-linking of the cysteine pair, the antibody-dimerized $Dyn1_{331kDa}$-α CL motor moved processively at a reduced average speed of ~11 nm/s (Fig. 7b, top right). In the presence of TCEP, the movement of the antibody-dimerized $Dyn1_{331kDa}$-α motor (109 ± 3 nm/s [± SEM; *N* = 281]; Fig. 7b, bottom right) was identical to that of the antibody-dimerized WT motor, $Dyn1_{331kDa}$, which moved at an average speed of ~110 nm/s under non-reducing (Fig. 7a, top right) and reducing (Supplemental Fig. 10b, top) conditions. To determine whether some of the moving $Dyn1_{331kDa}$-α motors were heterodimers consisting of a motor domain with non-cross-linked stalk helices ($Dyn1_{331kDa}$-α) and a motor domain with cross-linked stalk helices ($Dyn1_{331kDa}$-α CL) or homodimers of non-cross-linked $Dyn1_{331kDa}$-α motor domains, we first determined the velocity of the heterodimers by mixing the $Dyn1_{331kDa}$-α CL mutant with a two-fold higher concentration of WT $Dyn1_{331kDa}$ (a condition in which 44% of the motors are expected to be heterodimers, see legend to Supplemental Fig. 10). Analyzing the obtained velocity histogram with three velocity peaks yields a velocity of 43.4 ± 1.2 nm/s (mean ± SEM) for the $Dyn1_{331kDa}$-$Dyn1_{331kDa}$-α CL heterodimers when the measured velocities of $Dyn1_{331kDa}$-α CL (Fig. 7b, top right) and WT $Dyn1_{331kDa}$ (Fig. 7a, top right) are used as fixed parameters (see also legend to Supplemental Fig. 10). The velocity of the heterodimer is therefore ~40% of the velocity of the WT motor. Consistent with this measurement, Cleary et al. reported that a heterodimer of a WT motor domain fused to an SRS-stalk-MTBD construct in the presumed "α-

registration” (see discussion in the main text) moves at a threefold lower speed than the WT homodimeric motor[2]. That the WT $Dyn1_{331kDa}$-$Dyn1_{331kDa}$-α CL heterodimers move faster (relative to the WT motor) than the WT $Dyn1_{331kDa}$-SRS-stalk-MTBD heterodimers makes sense: while both motor domains of the $Dyn1_{331kDa}$-$Dyn1_{331kDa}$-α CL heterodimers can contribute to forward-directed motion by generating power strokes, only the WT motor domain of the $Dyn1_{331kDa}$-SRS-stalk-MTBD heterodimers is an active motor that can generate a power stroke (the SRS-stalk-MTBD construct acts just as a MT tether). With two motors generating power strokes instead of one, one might expect that the average velocity of the $Dyn1_{331kDa}$-$Dyn1_{331kDa}$-α CL heterodimers compared to the $Dyn1_{331kDa}$-SRS-stalk-MTBD heterodimers would have been more markedly increased, however, the relative velocity changes of the two constructs compared to the WT homodimers are still close. This may be explained by the fact that the $Dyn1_{331kDa}$-α CL motor domains with the cross-linked stalk helices—because they are cross-linked into the true α-registration—have a significantly higher MT-binding strength than the SRS-stalk-MTBD construct used by Cleary et al. (Fig. 4a-c).

With the knowledge of the velocity of the $Dyn1_{331kDa}$-$Dyn1_{331kDa}$-α CL heterodimers, we can now estimate the stalk cross-linking efficiency of the $Dyn1_{331kDa}$-α CL mutant by fitting two Gaussian functions to the histogram in Fig. 7b (top right): one centered at 10.6 nm/s (free parameter), and another one at 43.4 nm/s (the mean velocity and its standard deviation, SD = 20.2 nm/s [obtained from the analysis of the histogram in Supplemental Fig. 10c] are used as fixed parameters). The number of events in each bin under the Gaussian curve centered at 43.4 nm/s (see inset in Supplemental Fig. 10d) correspond to the number of $Dyn1_{331kDa}$-$Dyn1_{331kDa}$-α CL heterodimers (the number of the fast moving $Dyn1_{331kDa}$-$Dyn1_{331kDa}$ homodimers was too low to

fit a third Gaussian function). Calculation of the number of events under the two Gaussian functions as well as the scattered events of higher velocities generated by non-crosslinked homodimers yielded at total of 332 events: 290 events for the $Dyn1_{331kDa}$-α CL homodimers (comprised of 580 $Dyn1_{331kDa}$-α CL motor domains with cross-linked stalk helices), 35 events for the $Dyn1_{331kDa}$-$Dyn1_{331kDa}$-α CL heterodimers (comprised of 35 $Dyn1_{331kDa}$-α CL motor domains with cross-linked stalk helices and 35 $Dyn1_{331kDa}$-α motor domains without cross-linked stalk helices), and 7 events for the $Dyn1_{331kDa}$-α homodimeric motors (14 $Dyn1_{331kDa}$-α motor domains without cross-linked stalk helices). From a total number of 664 motor domains, 613 $Dyn1_{331kDa}$-α CL motor domains had cross-linked stalk helices, yielding a cross-linking efficiency of the stalk helices of 92.3%. However, as a result of the limited number of heterodimeric motors in this experiment, the Gaussian function centered at 43.4 nm/s slightly overestimates the true number of events under the curve that overlaps with the 10.6 nm/s peak (Supplemental Fig. 10d, inset). We therefore consider the ~92% cross-linking efficiency calculated here to be the lower bound. In addition, the ~95% cross-linking efficiency estimated for the β- and γ-mutants suggest that the increased time that passes under non-reducing conditions until the trapping experiments are performed (see protocol above) could also increase the cross-linking efficiency of the α-mutant to a value close to ~95% when performing the trapping experiments.

Supplementary Note 3

Oscillatory trap assay. The measurement of the force-dependent unbinding rates of the mouse SRS-α stalk-MTBD construct using the oscillatory trap assay (Supplementary Figs. 5-8) was done as previously described by Cleary and co-workers[2]. Briefly, a polystyrene bead coated with the SRS-α stalk-MTBD construct (at a concentration were less than 30% of the beads interacted

with the surface-attached MTs) is rapidly moved (v > 250 μm/s) between two positions (± 250 nm) every 2.5 seconds (Supplementary Fig. 5, left). When a bead-bound SRS construct binds to the MT before (most likely) or during the switching event, the bead eventually stops following the moving trap, during which time the SRS construct experiences a constant load given by the momentarily maintained bead-trap separation times the spring constant; however, before the constant-load phase of the assay is reached, the MTBD-MT bond experiences a rapidly changing loading rate (Supplementary Fig. 8). Once the SRS construct detaches, the bead quickly follows the trap to its new position (Supplementary Fig. 5). The waiting time between switching events was adjusted so that the detachments of the SRS constructs from the MTs occurred before the trap moved on to a new position. To prevent the measurement of multiple SRS constructs contributing to the linkage between the bead and MT, multi-step release events were discarded from the analysis[2]. The MT-bound times of the SRS-α stalk-MTBD construct under the various constant loads were then automatically detected and measured using a custom-written MATLAB program. The measured MT-bound times were then sorted by force and binned every ~100 data points and converted into cumulative distributions to allow a bin-size independent estimation of the force-dependent unbinding rates (Supplementary Fig. 6). The average detachment rate for each bin was then determined by fitting the experimental cumulative distributions to a theoretical cumulative distribution function (CDF) derived from a single exponential decay function:

$$CDF(t) = 1 - \exp{(-kt)},$$

were k is the characteristic unbinding rate (in s^{-1}). The nonlinear least squares fitting was performed using MATLAB. To estimate the error of the unbinding rate k, we bootstrapped 200 samples and fit them as described above. The standard deviation of the resulting set of unbinding rates was taken as the error estimate. The unbinding rate was then plotted with the average force of the data points in each bin (Supplementary Fig. 8a).

Supplementary Note 4

Calculation of force and loading rate for the oscillatory and constant-pulling assays, and discussion of the possible impacts of the calculated values on the performed bond-lifetime measurements. The loading rate is defined as the rate of change of the force on the molecular bond between the MTBD and the MT. The force on the molecular bond is given by the force-extension relation $F_m(x_B)$ for the bead position x_B. By differentiation, we obtain the time-dependent loading rate

$$\frac{dF_m}{dt} = \frac{dF_m}{dx_B}\frac{dx_B}{dt}.$$

We therefore have to calculate the derivative dF_m/dx_B of the force-extension relation and the velocity dx_B/dt of the bead to obtain the loading rate. Assuming an overdamped motion for the bead, its position is described by the differential equation

$$\gamma\frac{dx_B}{dt} = k\,(x_T - x_B) - F_m(x_B), \qquad \text{Eq. 1}$$

in which k is the spring constant of the optical trap, x_T is the position of the trap and γ is the hydrodynamic drag coefficient of the bead. As γ is influenced by the proximity of the bead to the cover slip surface, we calculate γ from the drag in bulk solution, $\gamma_0 = 3\pi d\eta$, according to Faxén's law[3]:

$$\gamma = \frac{\gamma_0}{1 - \frac{9R}{16} + \frac{1R^3}{8} - \frac{45R^4}{256} - \frac{1R^5}{16}}.$$

Here, $R = d/(d + 2Z_B)$, d the bead diameter, and Z_B the distance from the surface of the cover slip to the surface of the bead[4]. With a bead diameter of 870 nm and Z_B=50 nm, we obtain $\gamma = 2.3\gamma_0$. As the viscosity of water at 25 °C is 0.89 cP, γ_0 assumes a value of 7.3 nNs/m and we obtain

$\gamma = 16.8$ nNs/m. With a trap stiffness of 0.05 pN/nm used by Cleary et al.[2] and a trap displacement of $x_0 = 250$ nm, an initial speed (at $t = 0$) of 0.74 mm/s can be reached.

In the following, we demonstrate that the force-extension is related to the force-dependent loading rate as measured in the constant-pulling assay (Supplementary Fig. S3). In this assay, the strength of the molecular bond between the MTBD and MT is probed with the optical trap and the loading rate follows from a simple calculation of two springs in series, as given by[5]

$$r = \frac{k\,v}{1 + k\,\frac{dL}{dF_m}}, \qquad \text{Eq. 2}$$

in which k is the trap stiffness, v the pulling speed of the trap and dL/dF_m is the change of the molecule length L with respect to a change of force F_m. Consistency with our measured loading rates in Supplementary Figure 3 implies

$$F_m(L) = \hat{a}(e^{L/\hat{b}} - 1)$$

as an Ansatz for the force-extension relation. Using this Ansatz in Equation 2, and comparing it to the fit to the force-dependent loading rate measured for the SRS α-stalk-MTBD construct (Supplementary Fig. 3b), we obtain $\hat{a} = a = 0.23$ pN and $\hat{b} = (b - a)/k = 40$ nm.

In the oscillatory trap used by Cleary and co-workers[2], the trap is displaced by 250 nm. However, the motor can bind to different positions along the MT during the dwelling of the bead in-between the switching events so that the extension of the construct varies from switching event to switching event. If we assume that the SRS α-stalk-MTBD construct is soft and does not bind to the MT under tension, the maximum distance that the SRS α-stalk-MTBD construct can be theoretically stretched is 250 nm. In contrast, if the construct binds to a MT-binding site located toward the

switching direction and/or if the bead has to rotate before the construct is stretched, the total extension of the construct is less than the maximum 250 nm. To account for this variability of the total extension, we include the parameter L_0 in the force-extension relation in such a way that the construct is not stretched if $L < L_0$:

$$F_m(L) = \begin{cases} 0, & L < L_0 \\ \hat{a}(e^{L/\hat{b}} - 1), & L \geq L_0 \end{cases}. \qquad \text{Eq. 3}$$

For the oscillatory trap, we assume that the trap is displaced by 250 nm instantaneously. To calculate the position of the bead, its velocity, the force, and the loading rate, we combine Equations 2 and 3 and solve Equation 1 numerically for the initial conditions $x_B(0) = 0$ nm and $x_T(0) = 250\,\text{nm}$, and examine three different cases of molecular tension. By choosing $L_0 = 0$ nm, $L_0 = 90$ nm, and $L_0 = 165$ nm, the tension on the molecular bond of the SRS α-stalk-MTBD construct is 6.4 pN, 3 pN, and 1 pN, respectively (Supplementary Fig. 8a-c). The maximum force that can be measured when the trap is displaced by $\Delta x = 250$ nm is given by $F = k\Delta x = 12.5$ pN for a spring constant of 0.05 pN/nm. In this limit, the construct is infinitely stiff and the bead remains at the same position ($x_B = 0$ nm) although the trap is displaced. However, when we consider the compliance of the construct (see above), we obtain a theoretical maximal measurable force of ~6.5 pN. This suggests that the detachment forces measured with the oscillatory assay that are significantly larger than this value may be the result of two (or more) SRS α-stalk-MTBD constructs contributing to the linkage between the bead and MT, which could increase the overall stiffness. In overall agreement with this theoretical upper force limit, out of 473 single release events we measured in backward direction, less than 1.5% (7 events) were larger than 7 pN with a maximum measured value of 8.6 pN.

Our results show that loading rates between a few thousand up to almost 25,000 pN/s can be reached when using the oscillatory assay (Supplementary Fig. 8a-c). These loading rates are usually reached after a few hundreds of microseconds (~180 µs when establishing a final force of 6.4 pN, ~340 µs for 3 pN and ~470 µs for 1 pN). The time it takes to reach 63% (1/e value) of the final maximal force is similar: ~250 µs for 6.4 pN, ~320 µs for 3 pN and ~360 µs for 1 pN. In comparison to the constant-pulling experiments, which can be analyzed by combining Equation 1 with the pulling protocol $x_T(t) = v$ t, these are rather extreme numbers. In the constant-pulling assay used herein, loading rates are typically smaller than 5 pN/s and reached after a couple of seconds (Supplementary Fig. 8d and Fig. 2g).

The time during which the extreme loading rates act on the MTBD-MT bond of the SRS α-stalk-MTBD constructs when using the oscillatory assay are close to the timescales within which topological reorganizations of proteins occur[6]. It may therefore possible that the conformation of the MT-bound MTBD is affected by quick changes in tension even when the absolute force is in the lower pN range. That we only observe a significant discrepancy in the bond lifetimes obtained from both unbinding-force assays under forward load (Supplementary Fig. 7) suggests that the MTBD-MT bond of the SRS α-stalk-MTBD construct with the non-crosslinked stalk helices is more sensitive to external perturbations when the force is applied in forward direction.

Supplementary Note 5

Discussion of the effects of the cross linking the stalk helices of the SRS constructs. Our analysis revealed that the non-cross-linked SRS-α stalk-MTBD construct and the SRS-α stalk-MTBD construct with the stalk helices cross-linked in the α-registration (85:82 SRS-α stalk-MTBD CL)

show statistically different unbinding-force behaviors ($p_{ks} < 10^{-21}$ (forward) and $p_{ks} < 10^{-19}$ (backward) [SRS-α stalk-MTBD vs. SRS-α stalk-MTBD CL], Fig. 4b,c) and statistically different mean unbinding forces (SRS-α stalk-MTBD apo 1.1 [1.0, 1.2] pN vs. SRS-α stalk-MTBD CL apo 2.2 [2.0, 2.4], $p_m < 10^{-10}$ (forward) and SRS-α stalk-MTBD apo 1.2 [1.1, 1.2] pN vs. SRS-α stalk-MTBD CL apo 2.9 [2.6, 3.3], $p_m < 10^{-10}$ (backward)). As the SRS-α stalk-MTBD CL construct shows statistically indistinguishable unbinding-force behavior from the dynein motor domain with the cross-linked α-registration, $Dyn1_{331kDa}$-α CL ($p_{ks} = 0.63$ (forward) and $p_{ks} = 0.38$ (backward) [SRS-α stalk-MTBD CL vs. $Dyn1_{331kDa}$-α CL]), Figs. 3b and 4c), these data suggest that the SRS-α stalk-MTBD construct with the non-cross-linked stalk helices does not assume the α-registration. In contrast, the SRS-β stalk-MTBD construct shows the same unbinding behaviors whether the stalk helices are cross-linked in the β-registration or not ($p_{ks} = 0.76$ [SRS-β stalk-MTBD forward vs. SRS-β stalk-MTBD CL forward] and $p_{ks} = 0.42$ [SRS-β stalk-MTBD backward vs. SRS-β stalk-MTBD CL backward], Fig. 4d,e), suggesting that the SRS-β stalk-MTBD constructs assumes the β-registration with or without stalk cross-linking.

Interestingly, when paired cysteines were placed at the same positions as in the dynein motor domain with the cross-linked β-registration ($Dyn1_{331kDa}$-β CL mutant), the SRS-α stalk-MTBD construct could be cross-linked in the β-registration despite the apparent fusion of the dynein stalk to the coiled-coil base of SRS in the α-registration (Supplemental Fig. 4b). This result, together with the statistically different unbinding-force behaviors of the cross-linked and non-cross-linked SRS-α stalk-MTBD constructs, suggest that a thermodynamically-driven sliding of the helices between the α- and β-registrations (half a heptad) is possible in the absence of load (while the constructs are in solution) despite the inability of applied tension to cause registration changes in the non-crosslinked MT-bound SRS-α stalk-MTBD construct. This suggests that

registration changes in the MT-bound SRS-α stalk-MTBD construct are somehow prevented in the SRS construct when bound to MTs under applied directional tension.

Supplementary Note 6

Calculation of the unbinding rates from the unbinding-force histograms. To determine the force-dependent unbinding rates from the measured unbinding forces, Dudko and coworkers[7] introduced the relation for the unbinding rate,

$$\epsilon(F) = \frac{\dot{F}P(F)}{1-\int_0^F P(F')dF'},$$

in which $P(F)$ is the probability density of the unbinding forces and $\dot{F}$ is the force-dependent loading rate[1]. The denominator is also known as the survival function, $S(F) = 1 - CDF(F)$. The loading rate $\dot{F}$ is the change of the force applied to the bond per unit of time and depends on the stiffness of the trap, the compliance of the bond, and the pulling speed. As a first approximation and for proteins with negligible compliance, the loading rate can be estimated by the product of the pulling speed and the trap stiffness as we have done before[8]. However, to further improve the accuracy of the unbinding rates obtained from our unbinding-force experiments of single-headed dynein, we now take the motor's compliance indirectly into account by determining the force-dependent loading rates from the unbinding-force traces. To do so, we fit a line to the 200-ms trace segment preceding the detachment of the motor of each measured unbinding-force event. This

[1] We have previously shown that the Dudko method can be applied to more complex bonds (such as slip-ideal and catch-slip bonds) if the time scale of the experiment (the time of how fast one pulls on the bond) is long compared to the time scale of how fast the bond reaches steady state. At a loading rate of smaller than 5 pN/s in our constant-pulling assay (Fig. 2g), the force increases by less than 0.5 pN in 100 ms. In addition, the fastest unbinding rate we have measured herein is smaller than 10/s, which corresponds to a bond lifetime of 100 ms. As the typical equilibration time in proteins is less than a millisecond[17], the condition that we pull slowly enough to assume equilibrium is therefore fulfilled.

way, we obtained a loading rate (in pN/s) for each unbinding event. By binning all measured slopes according to their associated unbinding forces and averaging them in each bin (1-pN bins), we calculated an average force-dependent loading rate (Supplemental Fig. 3a). To further improve the statistics of our measurements, we measured the force-dependent loading rates for the WT motor, $Dyn1_{331kDa}$, and the α-mutant with the cross-linked stalk helices, both in forward and in backward direction. The fact that the force-dependent loading rates for all four data sets (two data sets for forward and backward directions for each construct) coincide, suggests that the compliance is similar for both constructs and neither depends on the direction of pulling nor on whether the stalk helices are cross linked. Therefore, we combined all measured slopes into single data set and calculated the average loading rate (Supplemental Fig. 3a, red dots). To obtain a theoretical approximation, we fit a rational function to this average loading rate using two free parameters and the maximum loading rate of 5.6 pN/s as a fixed parameter. This approach provides us with an excellent approximation for the force-dependent loading rate of the tail-truncated, single-headed *S. cerevisiae* dynein, which we use to convert the measured unbinding-force histograms into unbinding rates. We applied the same procedure to the data of the SRS constructs to obtain the force-dependent loading rate (see Supplemental Fig. 3b).

To determine $\epsilon(F)$, we have to evaluate $P(F)$ and $S(F)$. An estimator for these quantities has been suggested by Dudko and coworkers[7], based on bin size-dependent histograms. To improve statistics and to reduce the effects of bin size on the transformation of unbinding forces into unbinding rates, we are estimating $P(F)$ and $S(F)$ by applying a kernel density estimator to the measured unbinding forces using the statistical toolbox of MATLAB. Because the Dudko method is very sensitive to extreme large outliers, we only consider the unbinding events that lie within

the 99% area of the probability distribution of the measured data. In addition, the detection limit of our unbinding-force measurements is on the order of 0.3 pN. We therefore set the lower limit for the force range we evaluate to 0.5 pN.

A comparison between the histogram-based method, which takes all measured unbinding forces including extreme outliers into account, and the method using kernel density estimators (KDE), shows that for smaller bin sizes the probability density functions (PDFs) of both methods agree better and that both methods result in very similar force-dependent average unbinding rates. However, while the unbinding rates derived from the histogram-based method start to oscillate for increasing forces, the kernel density-based method results in a smooth progression of the unbinding rate with force.

To determine the force-dependent unbinding rates and the associated confident intervals (CIs), we wrote a MATLAB routine that uses the measured unbinding forces (to calculate unbinding rates) and the measured slopes (in pN/s) and the corresponding unbinding forces (to determine the force-dependent loading rates) as inputs. The 95% CIs were estimated by bootstrapping 4000 data sets containing two input data subsets, one resampled from the measured unbinding forces and one resampled from the measured slopes. The resampled data sets always had the same number of elements as the measured data sets. For each of the 4000 resampled data sets, we applied our MATLAB routine to determine the force-dependent loading rates and the force-dependent unbinding rates. The 95% CIs of the 4000 unbinding rates were obtained by estimating the area under the probability distribution with the MATLAB kernel density estimator.

Supplementary Figures:

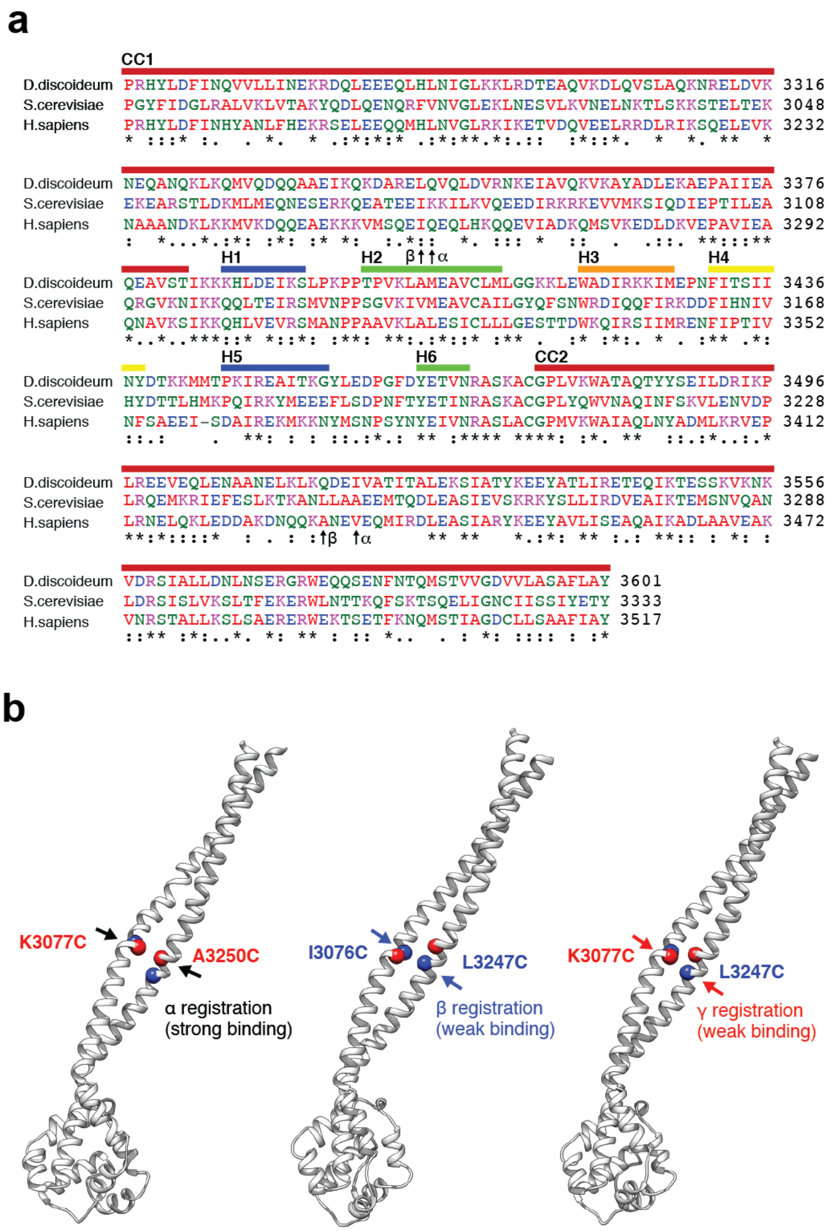

Supplementary Figure 1 Alignment of stalk and MTBD sequences and structures of the distal part of the stalk and MTBD in the strong, weak and intermediate MT-binding states. (**a**) Alignment of stalk and MTBD sequences from *Dictyostelium discoideum* (D. discoideum), *Saccharomyces*

cerevisiae (S. cerevisiae), and *Homo sapiens* (H. sapiens) cytoplasmic dynein. The positions of the cysteine mutations are indicated. The alignment was generated using Clustal Omega. (**b**) Structures of the distal one-half portion of the stalk helices and MTBD in the strong (α), weak (β), and intermediate (γ) MT-binding states created using the morphed structures described in the Supplementary Note 1.

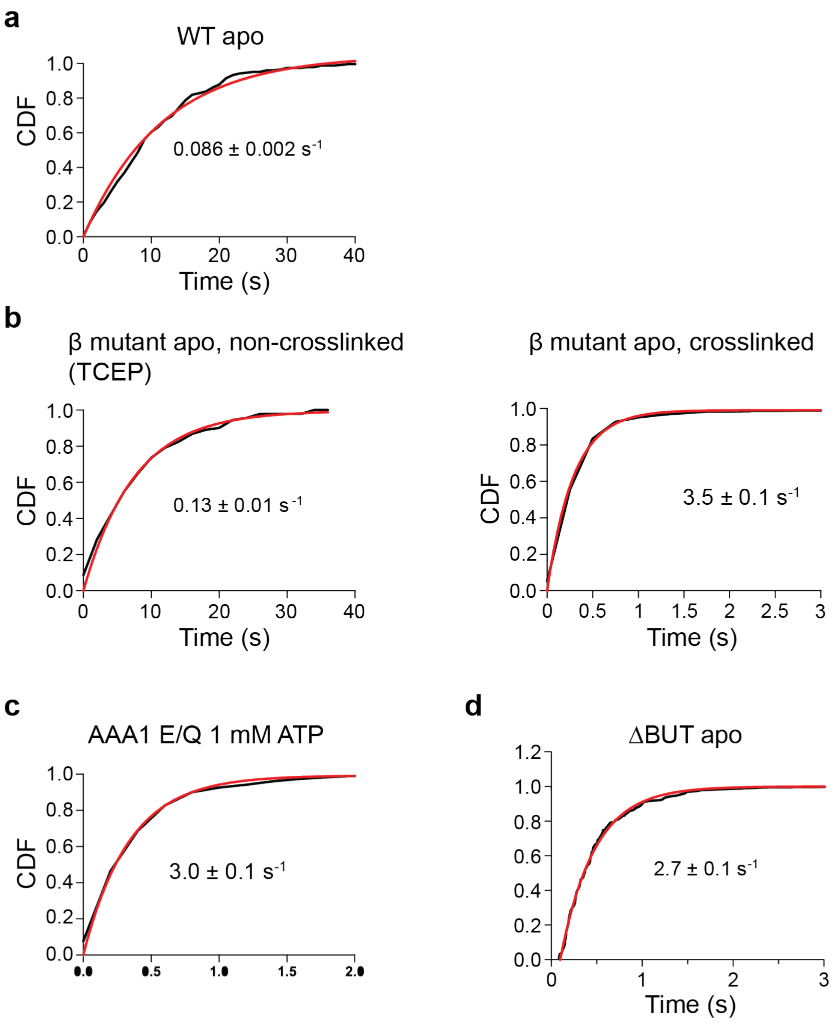

Supplementary Figure 2 Zero-load unbinding rates of Cy3-tagged WT and mutant dynein motor domains obtained from the analysis of the experimental CDFs of MT-bound lifetimes measured via TIRF microscopy. Fitting theoretical CDFs (red curves) derived from an exponential decay function to the experimental cumulative probabilities (black curves) yields the unbinding rate *k* for each motor construct (mean ± SEM). (**a**) WT dynein, $Dyn1_{331kDa}$, in the absence of nucleotides (apo) (N = 220). (**b**) Non-crosslinked β-mutant, $Dyn1_{331kDa}$-β + TCEP (left) (N = 91), and cross-linked β-mutant, $Dyn1_{331kDa}$-β (N = 351), both in apo state. (**c**) AAA1 ATP-hydrolysis mutant, AAA1 E/Q $Dyn1_{331kDa}$, in the presence of 1 mM ATP (N = 312). (**d**) Buttress-truncation mutant, ΔBUT-$Dyn1_{331kDa}$, in the absence of nucleotides (apo) (N = 190). Source data are provided as a Source Data file.

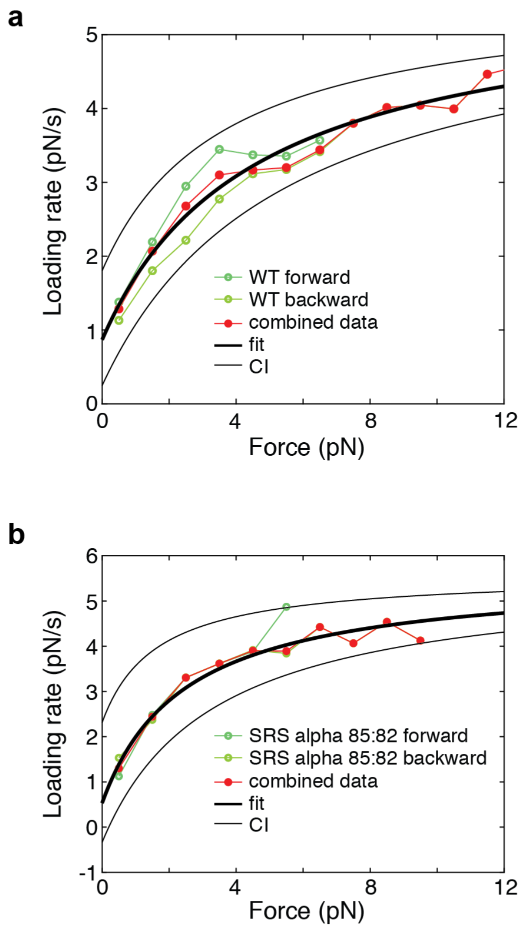

Supplementary Figure 3 Force-dependent loading rates for the WT motor, Dyn1$_{331kDa}$ (**a**) and the SRS-α stalk-MTBD construct with the cross-linked stalk helices (**b**) under MT plus- and minus-end-directed loading, determined as described in Supplementary Note 6. The data sets for MT plus- and minus-end-directed loading for each construct coincide and can be combined to calculate an average loading rate (red dots). The force-dependent average loading rate of the WT motor is fit to the rational function, $r(F) = 5.6\,pN\,s^{-1}(F + 0.70\,pN)/(4.5\,pN + F)$ (black line in part *a*). The average loading rate of the SRS construct (red dots in *b*) is fit to $r(F) = 5.6\,pN\,s^{-1}(F + 0.23\,pN)/(2.47\,pN + F)$ (black line in *b*). The 95% confidence intervals are given by MATLAB's fit function. Source data are provided as a Source Data file.

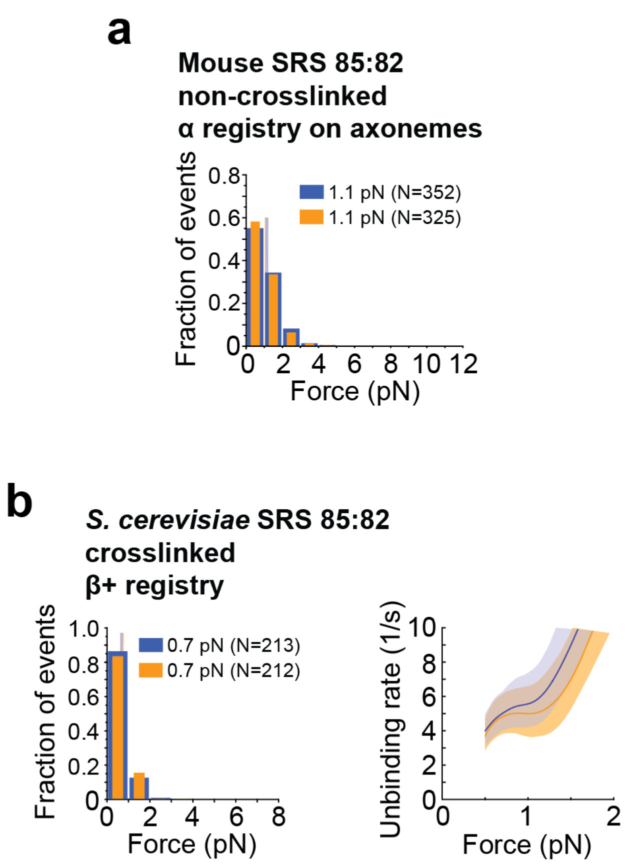

Supplementary Figure 4 Unbinding-force behaviors of SRS-stalk-MTBD constructs. (**a**) Normalized histograms of primary forward and backward unbinding forces for the SRS 85:82 construct with the mouse stalk helices (a.a. 3019-3309) fused in the non-crosslinked α-registry measured on axonemes, with mean values noted (95% CIs [1.1, 1.2] and [1.0, 1.2] pN), estimated by bootstrapping 4,000 samples). (**b**) As in *a* but for the SRS 85:82 construct with the *S. cerevisiae* stalk helices (a.a. 3019-3309) fused in the α-registry and cross-linked (I3076C, L3247C) in the β-registration (95% CIs [0.6, 0.7] and [0.7, 0.8] pN). Source data are provided as a Source Data file.

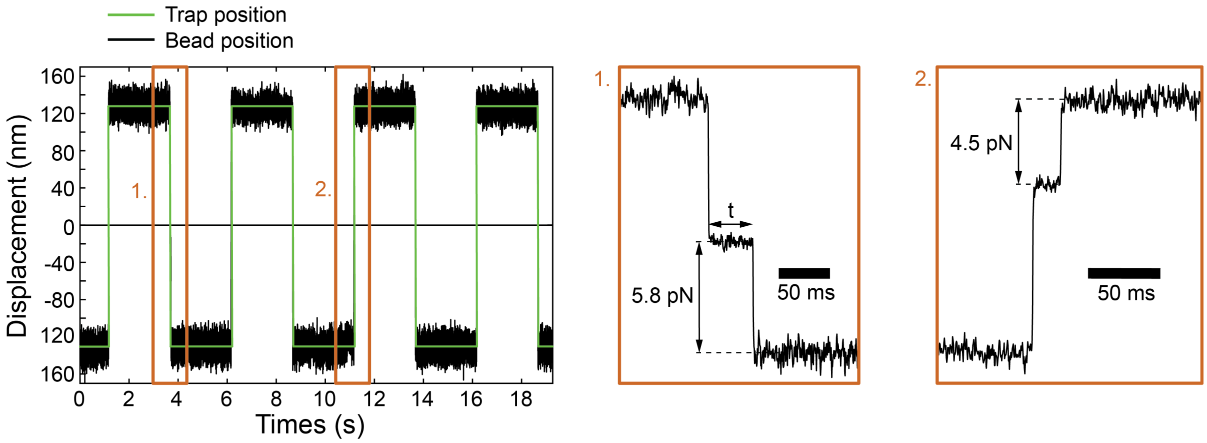

Supplementary Figure 5 Oscillatory trapping assay used by Cleary and co-workers[2]. A representative trace of trap (green) and bead position (black) is shown on the left. The two trace segments on the right correspond to the data marked by the rectangular boxes on the left. The first segment shows a binding event of lifetime *t* that occurs while the bead moves along the long MT axis toward the MT plus-end, while the second segment shows a binding event while the bead moves to the MT minus-end. As a result of the binding of the SRS construct to the MT surface, the bead stops following the moving trap, during which time the SRS construct experiences a constant load given by the momentarily maintained bead-trap separation times the spring constant. Once the SRS construct detaches, the bead quickly follows the trap to its new position (see Supplementary Note 3 for a detailed description of the oscillatory assay and Supplementary Note 4 for a theoretical analysis of the generated loading rates).

a Forward direction

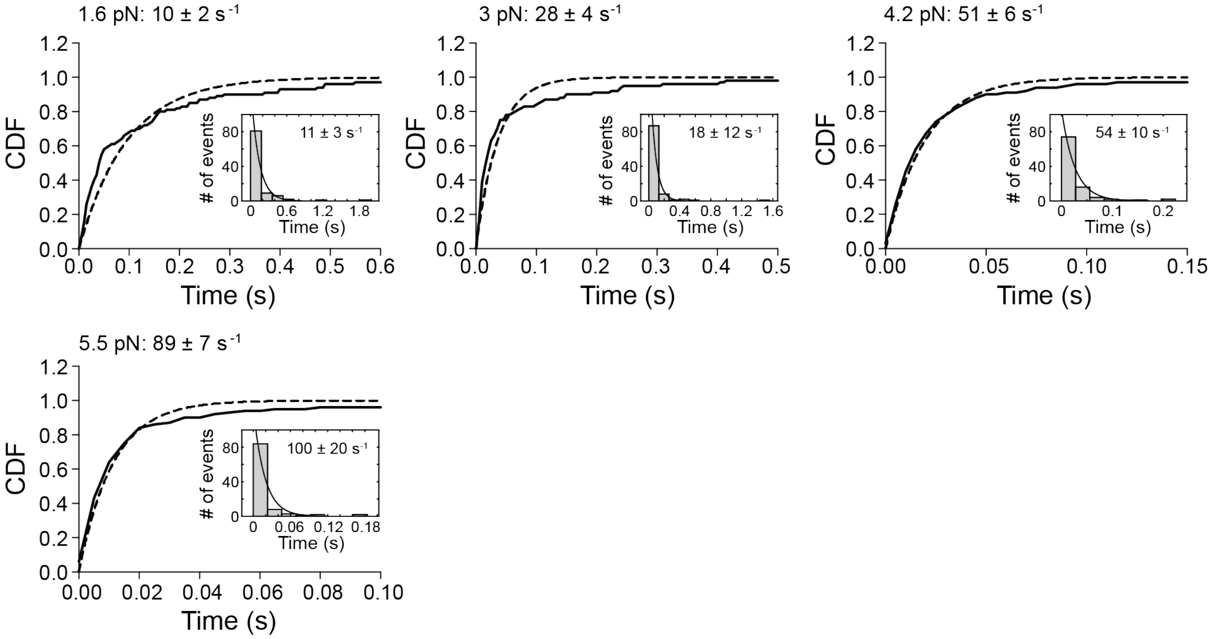

b

Backward direction

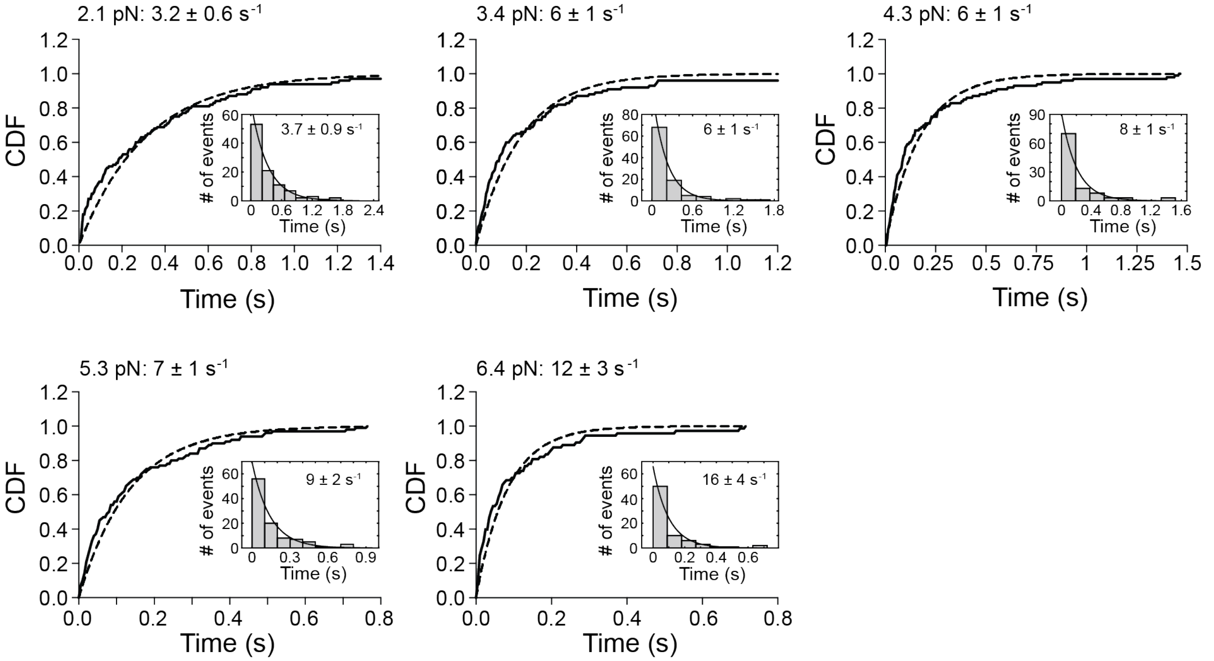

Supplementary Figure 6 Force-dependent unbinding rates of the SRS 85:82 construct with the non-crosslinked mouse stalk helices (a.a. 3019-3309) fused in the α-registry (SRS-α stalk-MTBD) measured with the oscillatory trap assay used by Cleary and co-workers[2]. (**a** and **b**) Cumulative probabilities of the measured bond lifetimes obtained under forward (panel *a*) and backward loading (panel *b*) following a sorting of the lifetimes by force and a binning of the data every 100 data points (the cumulative probabilities of the lifetimes in each force bin are shown). Fitting a theoretical cumulative distribution function (CDF) derived from an exponential decay function to the experimental cumulative probabilities (dashed curves) yields the unbinding rate k for each force value (depicted in Supplementary Fig. 7a). The given error (SE) of each unbinding rate was calculated by bootstrapping 200 samples (see Supplementary Note 3 for a detailed description of the processing and analysis of the oscillatory trap data). For comparison, we also show the unbinding rates that result from fitting the histograms of the measured lifetimes of each force bin to an exponential function as done by Cleary and co-workers[2]. Here, we calculated the optical bin size using the MATLAB calcnbins() function (setting 'middle'). The resulting rates agree with the rates obtained via CDF analysis within the estimated errors. As the CDF analysis provides a bin size-independent analysis, we plot the unbinding rates obtained from the CDF analysis in Supplementary Fig. 7a. Source data are provided as a Source Data file.

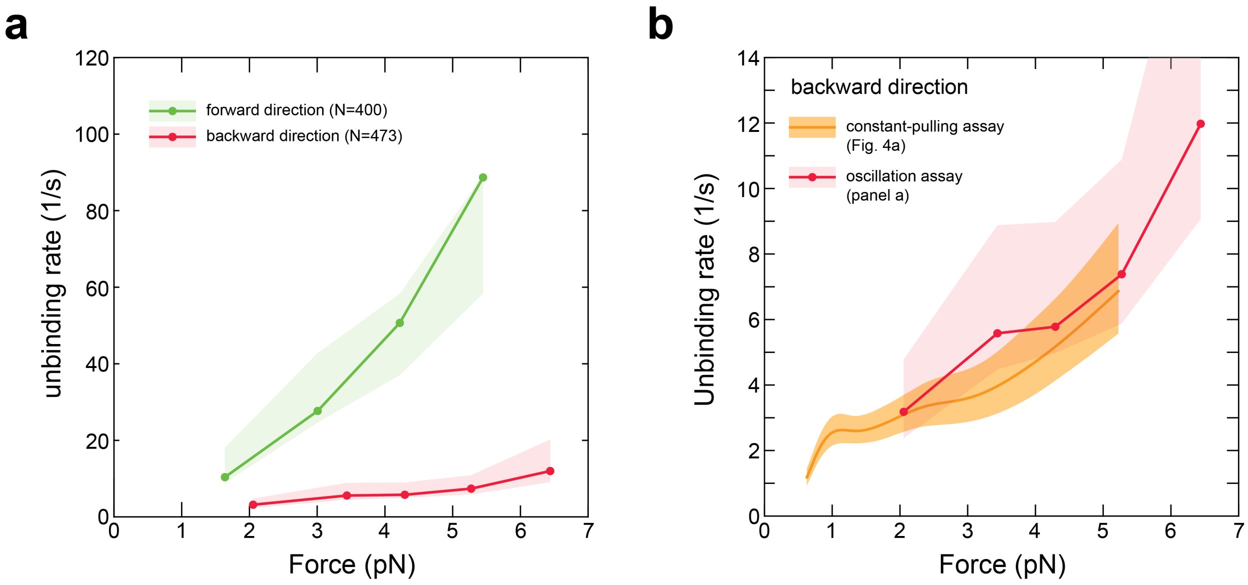

Supplementary Figure 7 Unbinding rate vs. force for the SRS-α stalk-MTBD construct. (a) Unbinding rate vs. force measured with the oscillatory assay used by Cleary and co-workers[2] (see Supplementary Fig. 6 for the data underlying the depicted values and Supplementary Note 3 for a detailed description of the generation and analysis of the oscillatory trap data). The shaded areas are 95% CIs for the mean rates, estimated by bootstrapping. Our analysis shows that the oscillatory trap assay results in an anisotropic detachment rate as previously reported by Cleary and co-workers[2]. (**b**) Unbinding rate as a function of backward load (applied along the long MT axis) measured with the oscillatory trap assay (panel a) and the constant-pulling assay used herein (Fig. 4a).

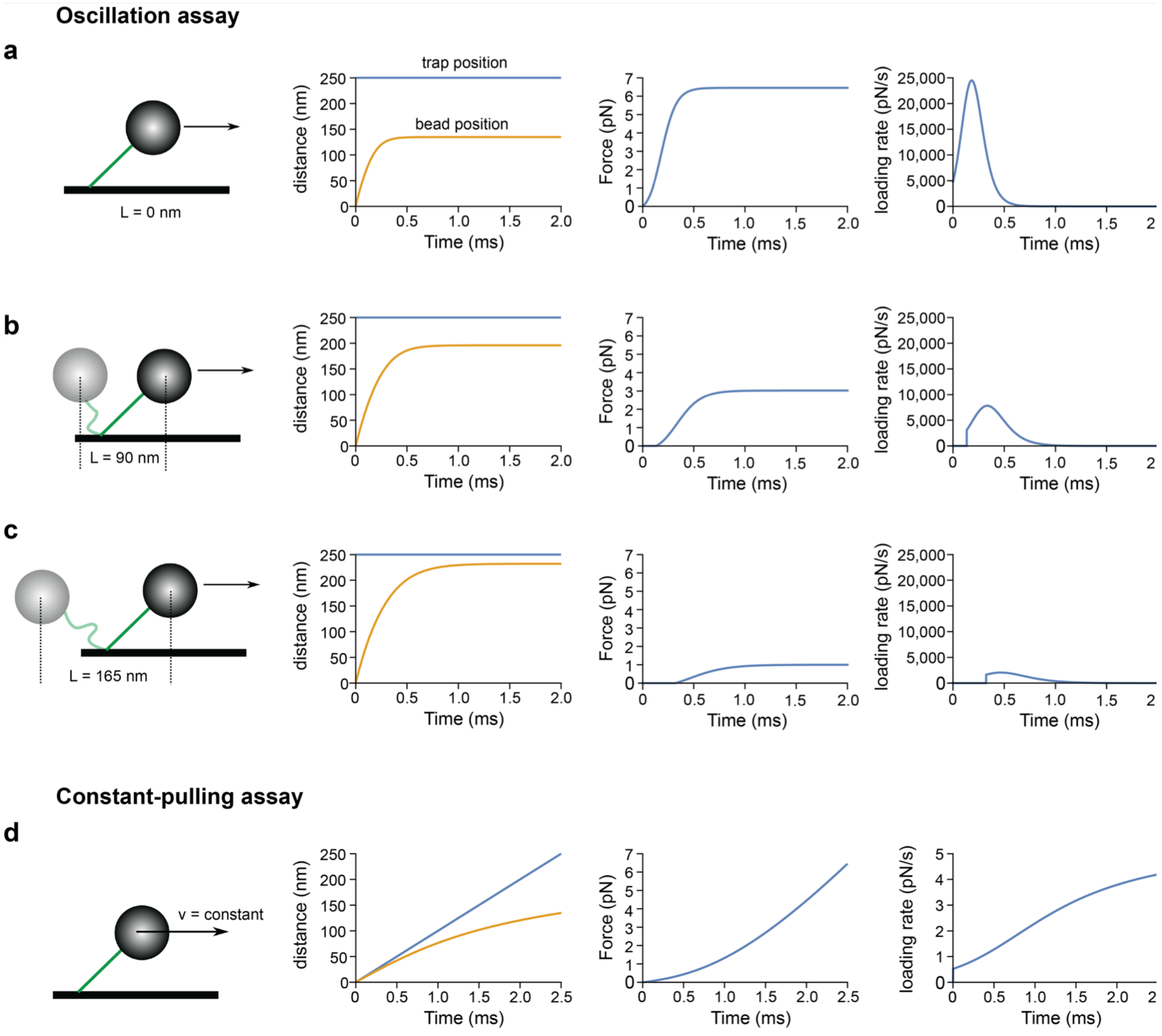

Supplementary Figure 8 Theoretical analysis of bead position, force and loading rate generated by the oscillatory and constant-pulling assays as discussed in Supplementary Note 4.

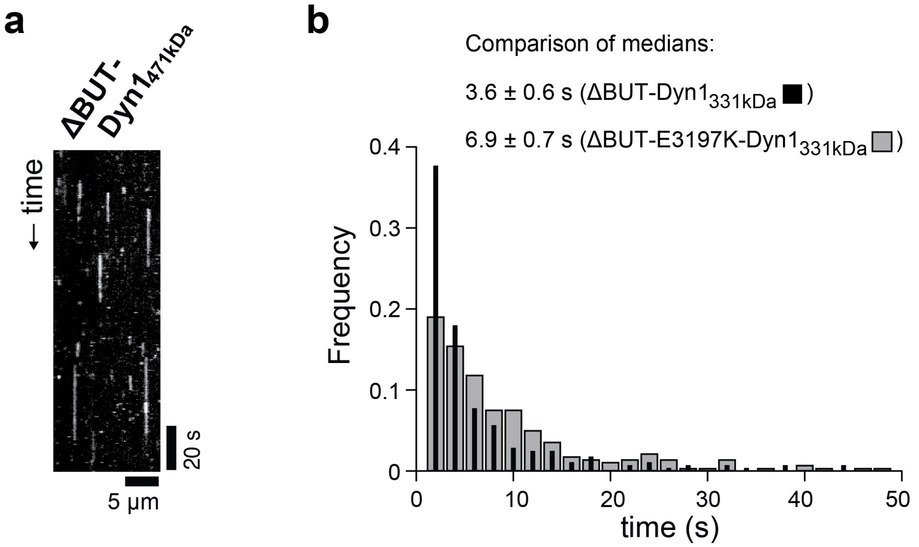

Supplementary Figure 9 Motility analysis of the homodimeric motor, ΔBUT-Dyn1$_{471kDa}$, and comparison of the MT-bound lifetimes of the antibody-dimerized motors, ΔBUT-Dyn1$_{331kDa}$ and ΔBUT-E3197K-Dyn1$_{331kDa}$. (**a**) Kymograph analysis reveals that ΔBUT-Dyn1$_{471kDa}$ only shows non-motile interactions. (**b**) Plotting the histograms of the MT-bound lifetimes of the ΔBUT-Dyn1$_{331kDa}$ and ΔBUT-E3197K-Dyn1$_{331kDa}$ motors acquired in the presence of 1 mM ATP reveals that the increased ionic strength between MTs and the MTBD via the E3197K mutation increases the MT-bound lifetime of the non-motile binding events: 3.6 ± 0.6 s (median ± SEM; N = 262) [ΔBUT-Dyn1$_{331kDa}$] vs. 6.9 ± 0.7 s^{-1} (median ± SEM; N = 257) [ΔBUT-E3197K-Dyn1$_{331kDa}$] (source data are provided as a Source Data file).

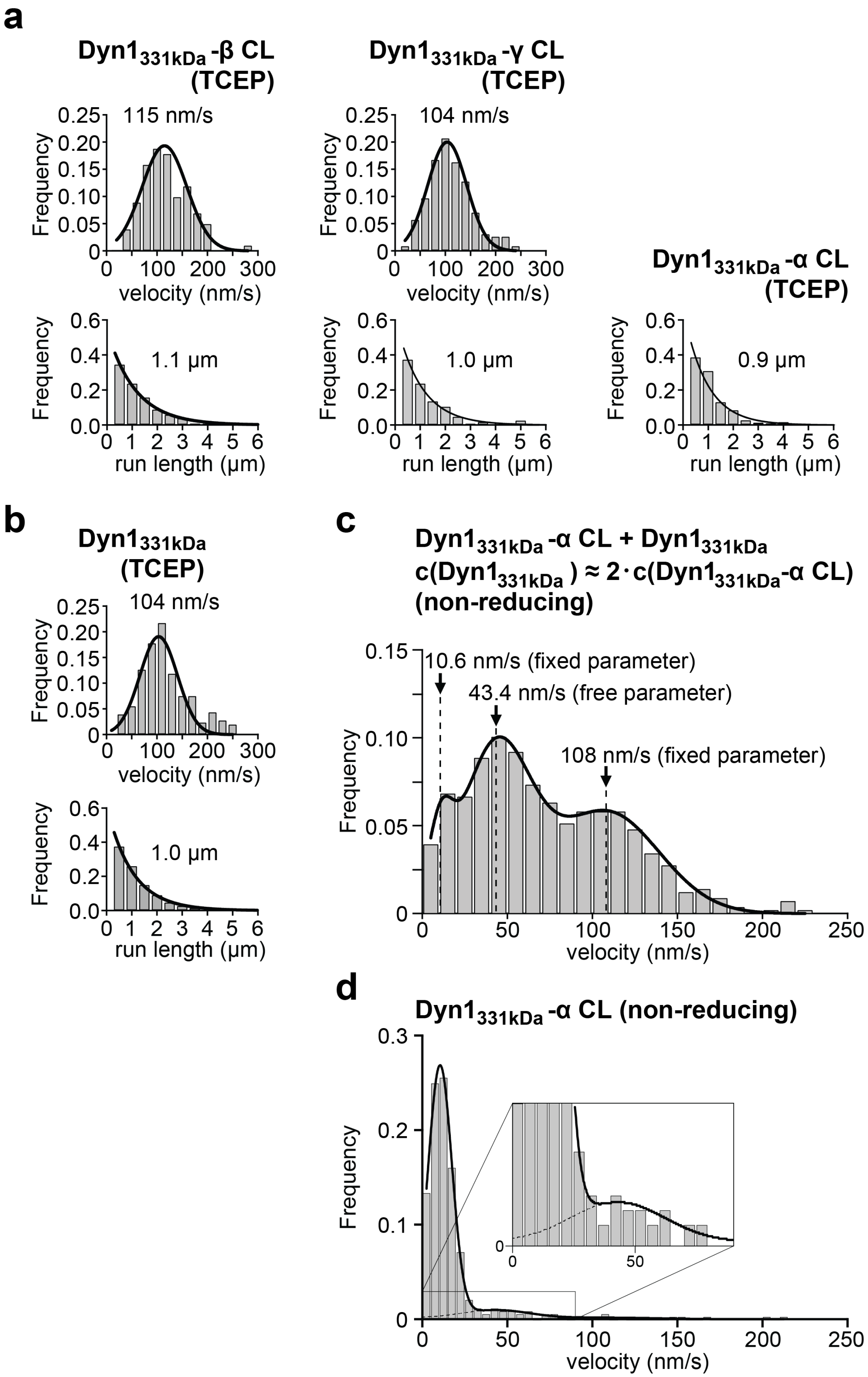

a
Dyn1331kDa -β CL (TCEP)
115 nm/s
Frequency
velocity (nm/s)
1.1 µm
run length (µm)
Dyn1331kDa -γ CL (TCEP)
104 nm/s
1.0 µm
Dyn1331kDa -α CL (TCEP)
0.9 µm
b
Dyn1331kDa (TCEP)
104 nm/s
1.0 µm
c
Dyn1331kDa -α CL + Dyn1331kDa
c(Dyn1331kDa) ≈ 2·c(Dyn1331kDa-α CL)
(non-reducing)
10.6 nm/s (fixed parameter)
43.4 nm/s (free parameter)
108 nm/s (fixed parameter)
d
Dyn1331kDa -α CL (non-reducing)

Supplementary Figure 10 Velocity and processivity analyses of antibody-dimerized mutant and WT motors under reducing (non-cross-linking) and non-reducing (cross-linking) conditions. (**a**) TIRF-based motility experiments conducted in the presence of 2 mM TCEP demonstrate that the cross-linking of the stalk helices is fully reversible and that the mutant motors, $Dyn1_{331kDa}$-β CL, $Dyn1_{331kDa}$-γ CL, $Dyn1_{331kDa}$-α CL, are as enzymatically active under reducing conditions as WT $Dyn1_{331kDa}$. Fitting the acquired velocity and run length histograms with Gaussian and first-order decay functions yields mean velocities of 115 ± 10 nm/s (mean ± SEM; *N* = 101) for $Dyn1_{331kDa}$-β CL and 104 ± 3 nm/s (mean ± SEM; *N* = 227) for $Dyn1_{331kDa}$-γ CL (see Fig. 7b in the main text for the mean velocity of the $Dyn1_{331kDa}$-α CL motor in the presence of TCEP). Characteristic run lengths were 1.1 ± 0.1 μm (± SEM; *N* = 101) for $Dyn1_{331kDa}$-β CL, 1.0 ± 0.1 μm (± SEM; *N* = 227) for $Dyn1_{331kDa}$-γ CL, and 0.9 ± 0.1 μm (± SEM; *N* = 281) for $Dyn1_{331kDa}$-α CL. These values are similar to the values of the WT motor both under non-reducing (Fig. 7a, top right) and reducing (panel *b*) conditions. (**b**) Kymograph-based analysis of the antibody-dimerized WT $Dyn1_{331kD}$ motor under reducing conditions (2 mM TCEP) results in a velocity (104 ± 2 nm/s [± SEM; *N* = 253]) and run length (1.0 μm ± 0.1 [± SEM; *N* = 253]), which are similar to the velocity and run length of the antibody-dimerized WT motor measured under non-reducing conditions (Fig. 6a, top right). This demonstrates that the cross-linking conditions do not affect the function of the WT motor. (**c**) Velocity histogram (*N* = 594) of populations of antibody-dimerized homodimeric ($Dyn1_{331kDa}$-α CL or WT $Dyn1_{331kDa}$) and heterodimeric motors ($Dyn1_{331kDa}$-α CL motor domain coupled to a WT $Dyn1_{331kDa}$ motor domain) created under non-reducing (cross-linking) conditions by combining the $Dyn1_{331kDa}$-α CL mutant with a two-fold higher concentration of WT $Dyn1_{331kDa}$. The velocity of the heterodimers (43.4 ± 1.2 nm/s [± SEM; $N_{heterodimer}$ = 285]) was determined by

fitting the histogram to three Gaussian functions. The velocity peaks of the homodimeric $Dyn1_{331kDa}$-α CL motor (10.6 ± 1.2 nm/s [± SEM; $N_{mutant\_homodimer}$ = 36]; Fig. 7b, top right) and the homodimeric WT $Dyn1_{331kDa}$ motor (108 ± 2 nm/s [± SEM; $N_{WT\_homodimer}$ = 273]; Fig. 7a, top right) were used as fixed parameters, while the mean velocity of the heterodimer was a free parameter. The standard deviation of the mean velocity of the $Dyn1_{331kDa}$-α CL homodimer (7 nm/s), taken from the fit of the Gaussian function to the velocity peak of $Dyn1_{331kDa}$-α CL under cross-linking conditions (Fig. 7b, top right), was also taken as a fixed parameter. The number of events for each dimeric motor species was determined from the relative surface areas of each Gaussian function; the calculated 6, 48 and 46% contributions of the $Dyn1_{331kDa}$-α CL homodimer, the $Dyn1_{331kDa}$-α CL-$Dyn1_{331kDa}$ heterodimer and the $Dyn1_{331kDa}$ homodimer, are reasonably close to the theoretically expected contributions of 10, 44, and 46% assuming the WT $Dyn1_{331kDa}$ concentration is twice as high (2.1x) as the concentration of the $Dyn1_{331kDa}$-α CL mutant. (**d**) Fitting the velocity histogram of the $Dyn1_{331kDa}$-α CL mutant motors acquired under cross-linking conditions (Fig. 7b, top right) with two Gaussian functions, one centered at 10.6 nm/s (free parameter) and another at 43.4 nm/s (the mean velocity and its standard deviation, SD = 20.2 nm/s, obtained from the analysis in panel *c*, were taken as fixed parameters), allows the estimation of the fractions of $Dyn1_{331kDa}$-α CL homodimers, $Dyn1_{331kDa}$-α CL-$Dyn1_{331kDa}$-α heterodimers, and $Dyn1_{331kDa}$-α homodimers (see Supplementary Note 2 for more information). Source data are provided as a Source Data file.

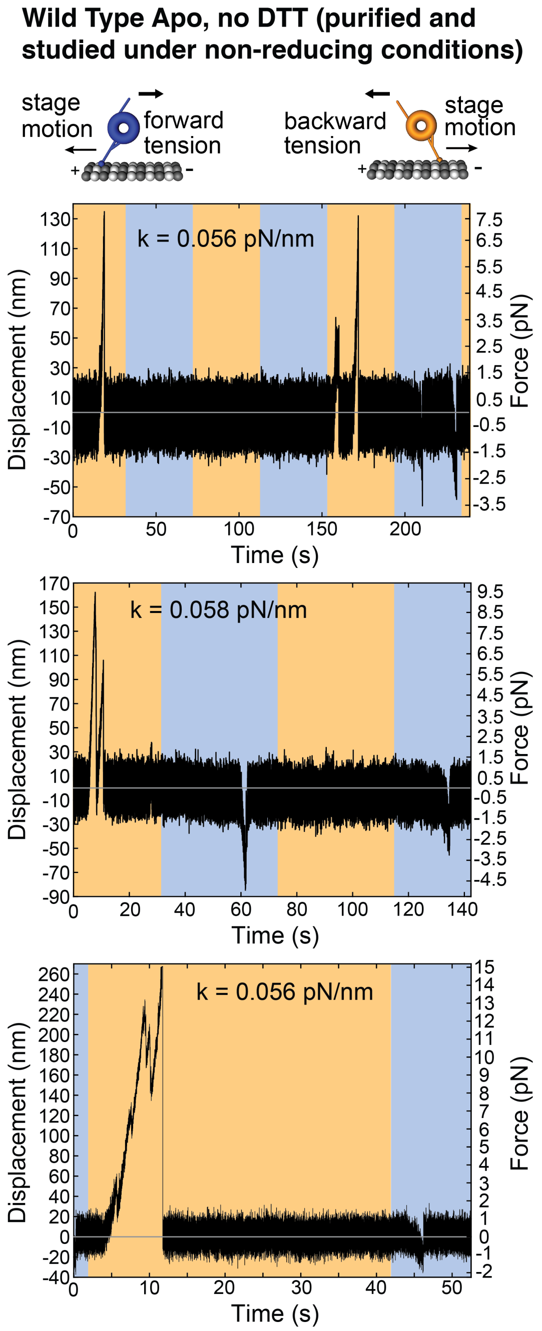

Supplementary Figure 11 Unbinding-force experiments (force/position vs. time) in the absence of nucleotide for the WT motor domain, $Dyn1_{331kDa}$ (VY137). The illustration represents the configuration for backward vs. forward tension. Orange (blue) shaded areas show periods of applied rearward (forward) tension.

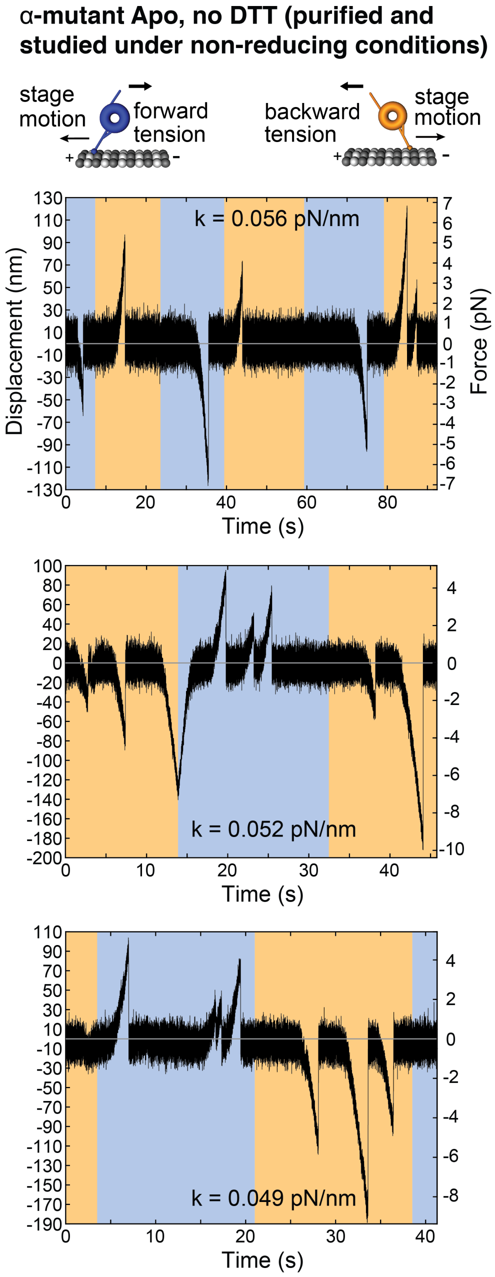

Supplementary Figure 12 Unbinding-force experiments (force/position vs. time) in the absence of nucleotide for the α-mutant motor domain with the cross-linked stalk helices, Dyn1$_{331kDa}$-α CL. The illustration represents the configuration for backward vs. forward tension. Orange (blue) shaded areas show periods of applied rearward (forward) tension.

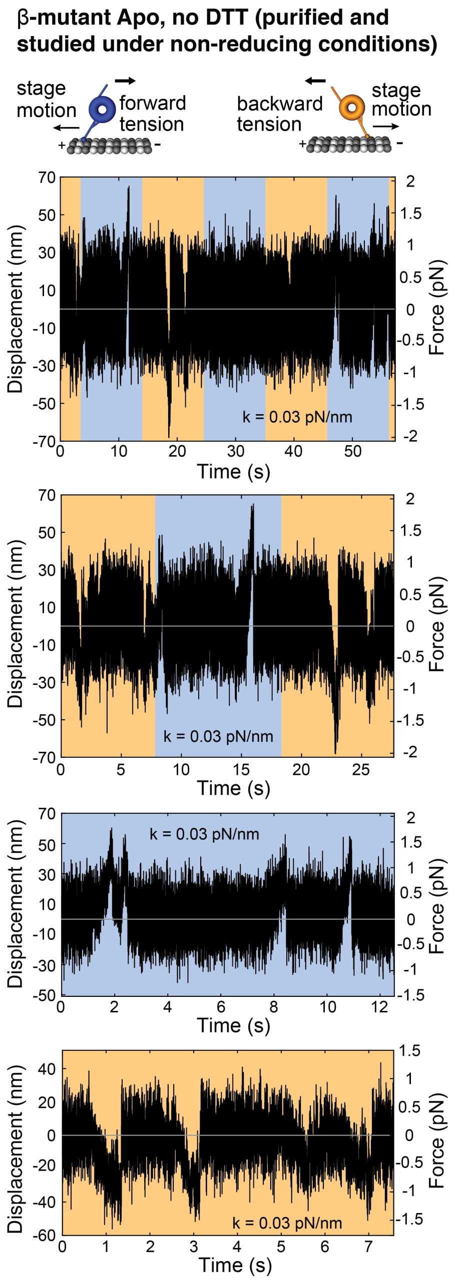

Supplementary Figure 13 Unbinding-force experiments (force/position vs. time) in the absence of nucleotide for the β-mutant motor domain with the cross-linked stalk helices, $Dyn1_{331kDa}$-β CL. The illustration represents the configuration for backward vs. forward tension. Orange (blue) shaded areas show periods of applied rearward (forward) tension.

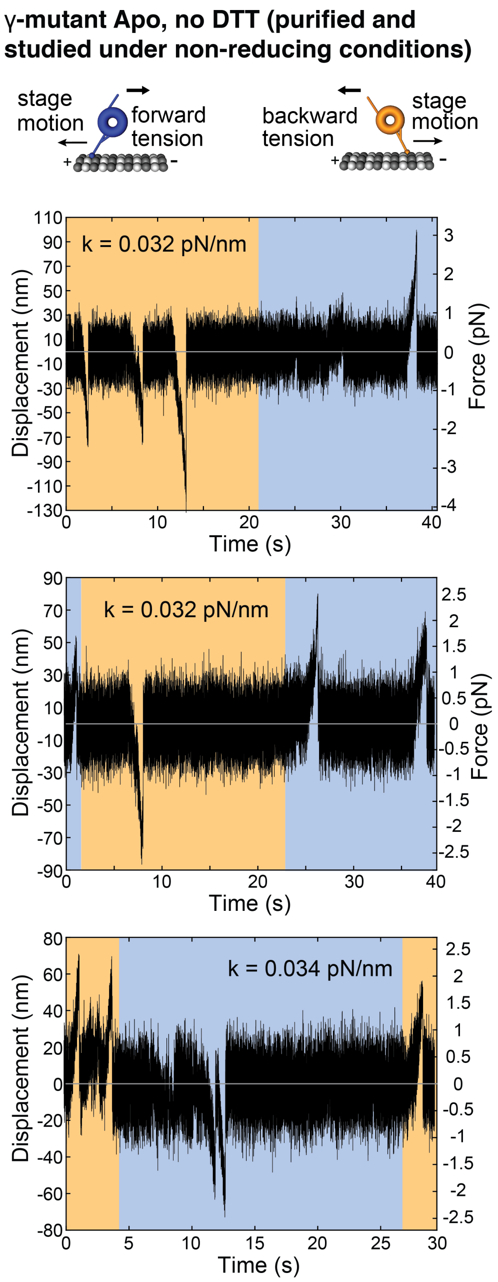

Supplementary Figure 14 Unbinding-force experiments (force/position vs. time) in the absence of nucleotide for the γ-mutant motor domain with the cross-linked stalk helices, Dyn1$_{331kDa}$-γ CL. The illustration represents the configuration for backward vs. forward tension. Orange (blue) shaded areas show periods of applied rearward (forward) tension.

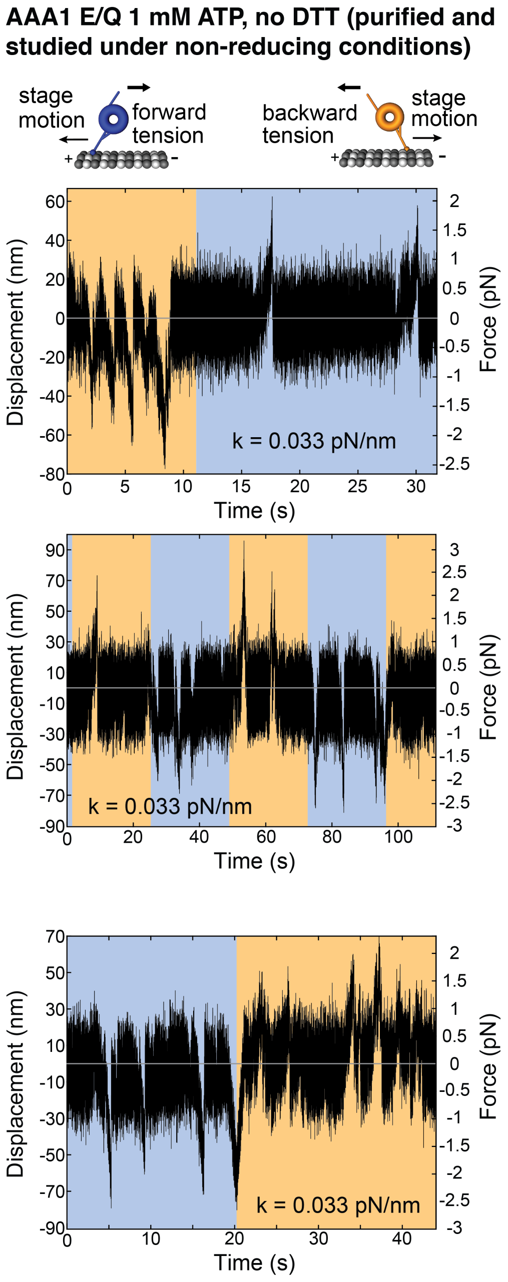

Supplementary Figure 15 Unbinding-force experiments (force/position vs. time) in the presence of 1 mM ATP for the AAA1 E/Q mutant motor domain. The illustration represents the configuration for backward vs. forward tension. Orange (blue) shaded areas show periods of applied rearward (forward) tension.

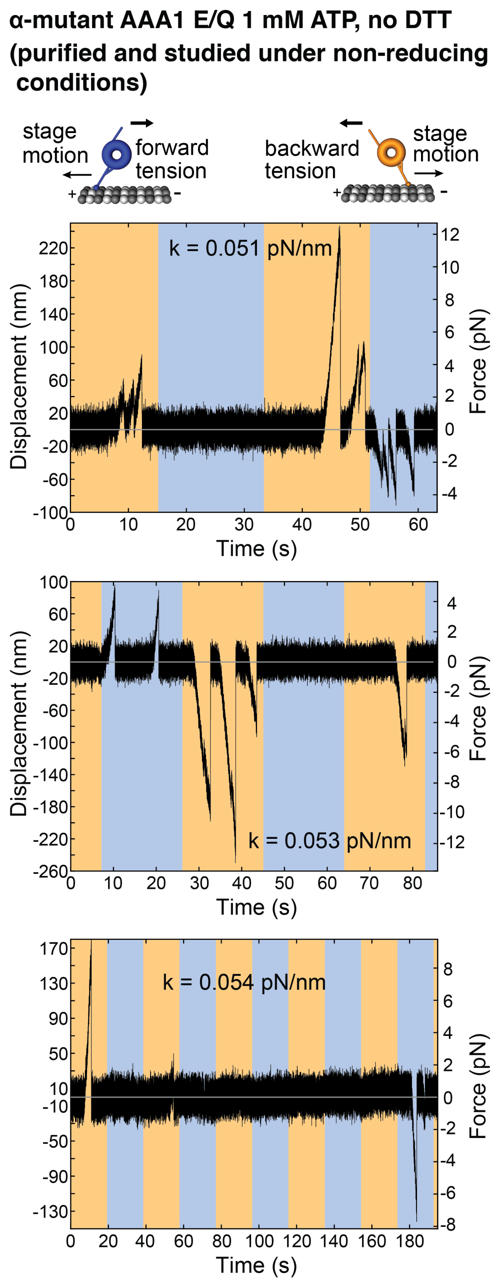

Supplementary Figure 16 Unbinding-force experiments (force/position vs. time) in the presence of 1 mM ATP for the AAA1 E/Q mutant motor domain with the stalk helices cross-linked in the α-registration (AAA1 E/Q Dyn1$_{331kDa}$-α CL). The illustration represents the configuration for backward vs. forward tension. Orange (blue) shaded areas show periods of applied rearward (forward) tension.

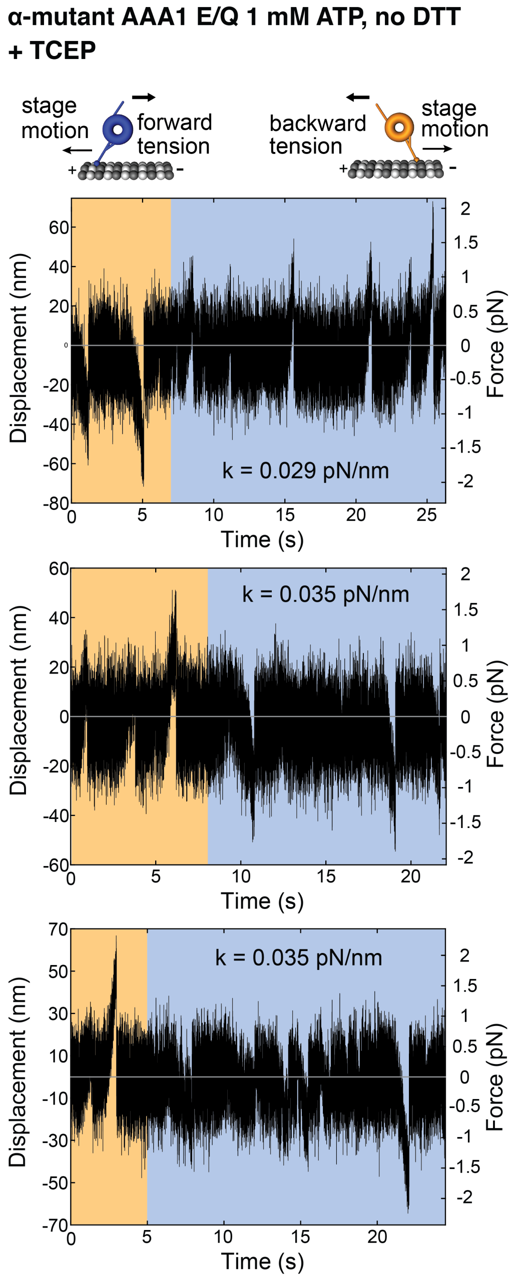

Supplementary Figure 17 Unbinding-force experiments (force/position vs. time) in the presence of 1 mM ATP and 2 mM TCEP for AAA1 E/Q $Dyn1_{331kDa}$-α CL. The illustration represents the configuration for backward vs. forward tension. Orange (blue) shaded areas show periods of applied rearward (forward) tension.

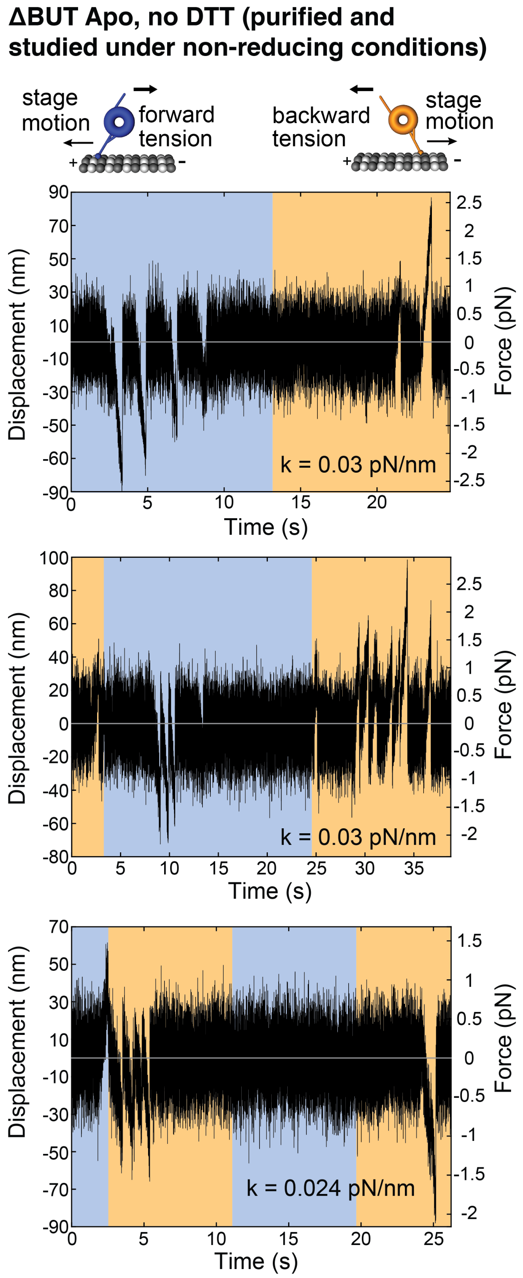

Supplementary Figure 18 Unbinding-force experiments (force/position vs. time) in the absence of nucleotide for the buttress-truncation mutant ΔBUT-Dyn1$_{331kD}$. The illustration represents the configuration for backward vs. forward tension. Orange (blue) shaded areas show periods of applied rearward (forward) tension.

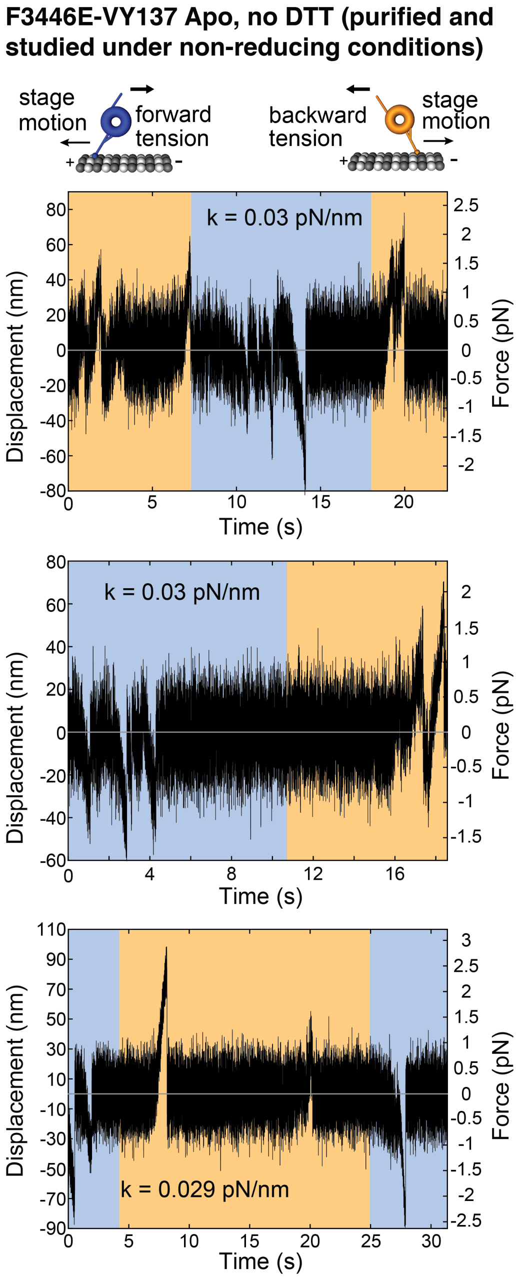

Supplementary Figure 19 Unbinding-force experiments (force/position vs. time) in the absence of nucleotide for the F3446E-Dyn1$_{331kDa}$ mutant motor domain. The illustration represents the configuration for backward vs. forward tension. Orange (blue) shaded areas show periods of applied rearward (forward) tension.

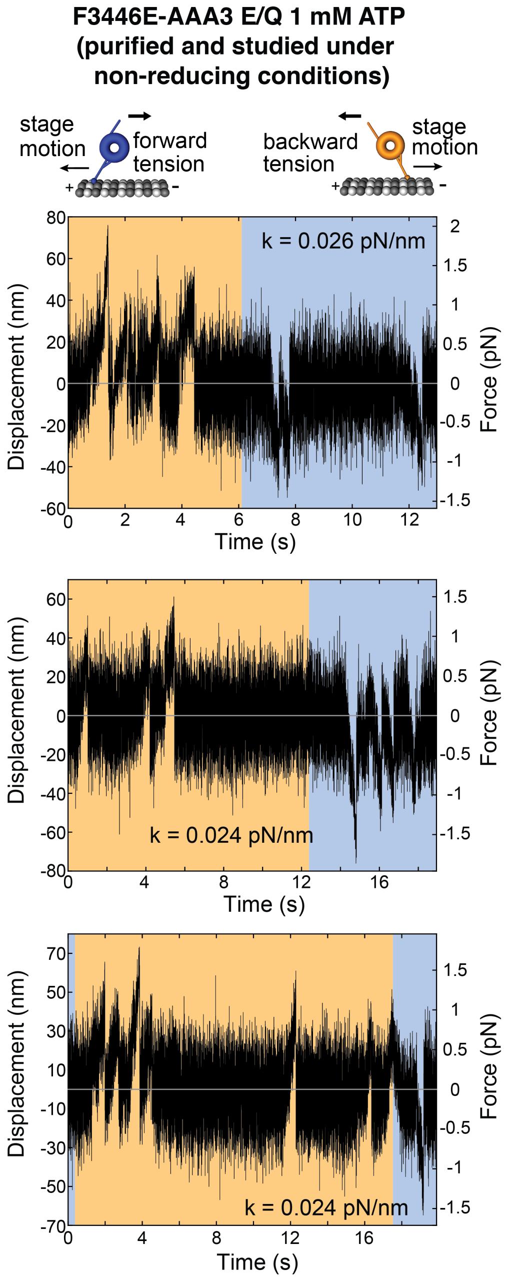

Supplementary Figure 20 Unbinding-force experiments (force/position vs. time) in the presence of 1 mM ATP for the F3446E-AAA3 E/Q CT-GFP mutant motor domain. The illustration represents the configuration for backward vs. forward tension. Orange (blue) shaded areas show periods of applied rearward (forward) tension.

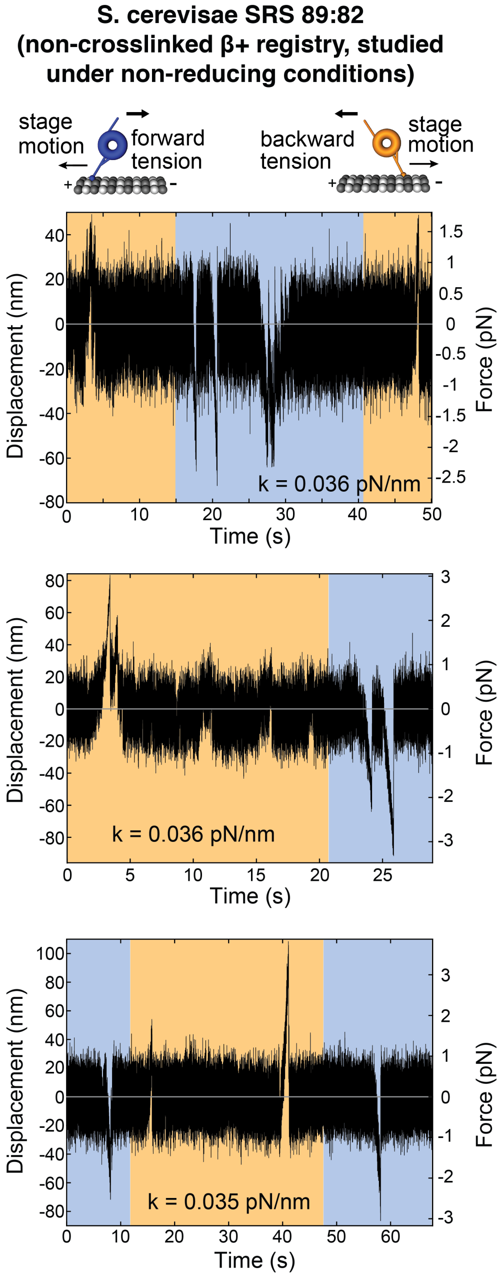

Supplementary Figure 21 Unbinding-force experiments (force/position vs. time) for the SRS-β stalk-MTBD construct with the *S. cerevisiae* stalk-MTBD sequence and non-cross-linked stalk helices. The illustration represents the configuration for backward vs. forward tension. Orange (blue) shaded areas show periods of applied rearward (forward) tension.

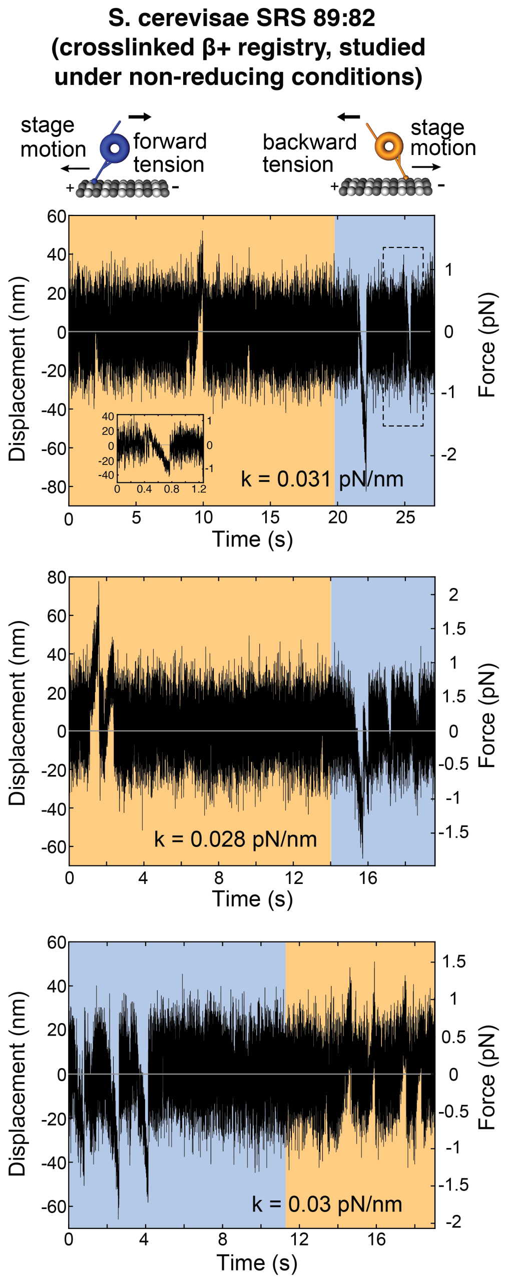

Supplementary Figure 22 Unbinding-force experiments (force/position vs. time) for the SRS-β stalk-MTBD CL construct with the *S. cerevisiae* stalk-MTBD sequence and cross-linked β-registry. The illustration represents the configuration for backward vs. forward tension. Orange (blue) shaded areas show periods of applied rearward (forward) tension.

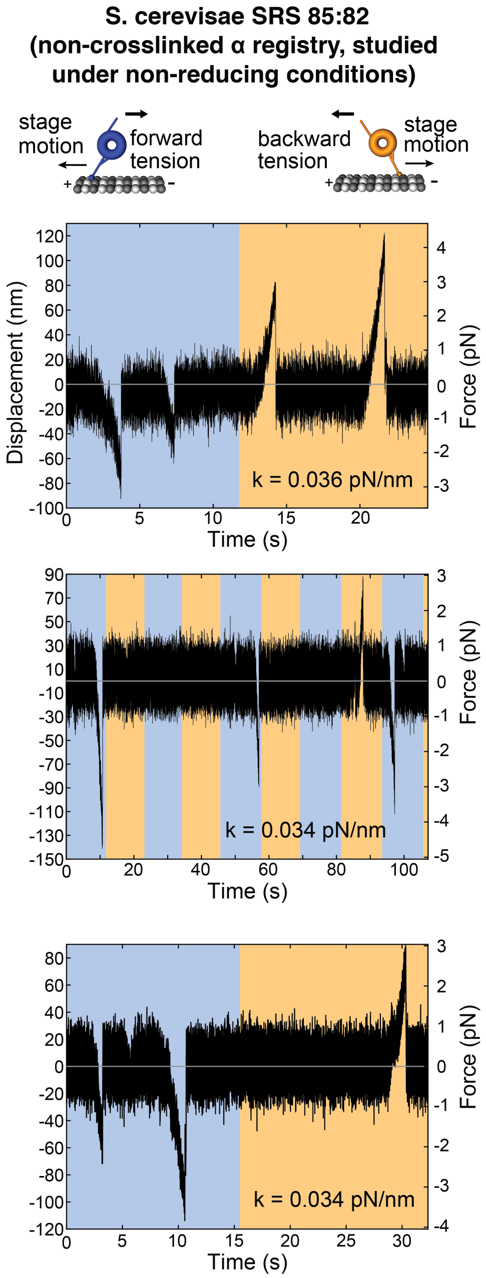

Supplementary Figure 23 Unbinding-force experiments (force/position vs. time) for the SRS-α stalk-MTBD construct with the *S. cerevisiae* stalk-MTBD sequence and non-cross-linked stalk helices. The illustration represents the configuration for backward vs. forward tension. Orange (blue) shaded areas show periods of applied rearward (forward) tension.

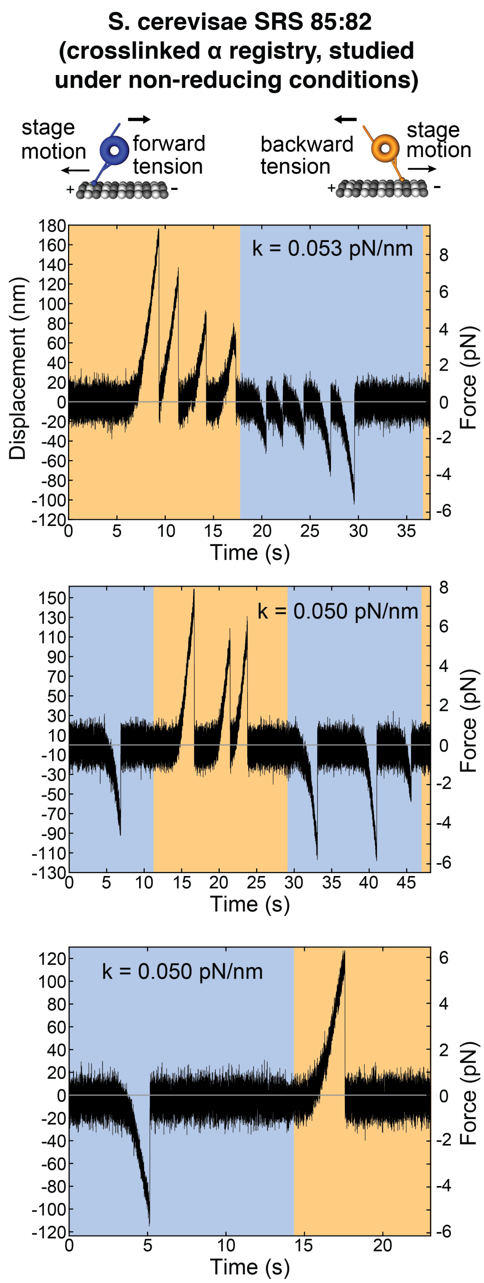

Supplementary Figure 24 Unbinding-force experiments (force/position vs. time) for the SRS-α stalk-MTBD CL construct with the *S. cerevisiae* stalk-MTBD sequence and cross-linked α-registry. The illustration represents the configuration for backward vs. forward tension. Orange (blue) shaded areas show periods of applied rearward (forward) tension.

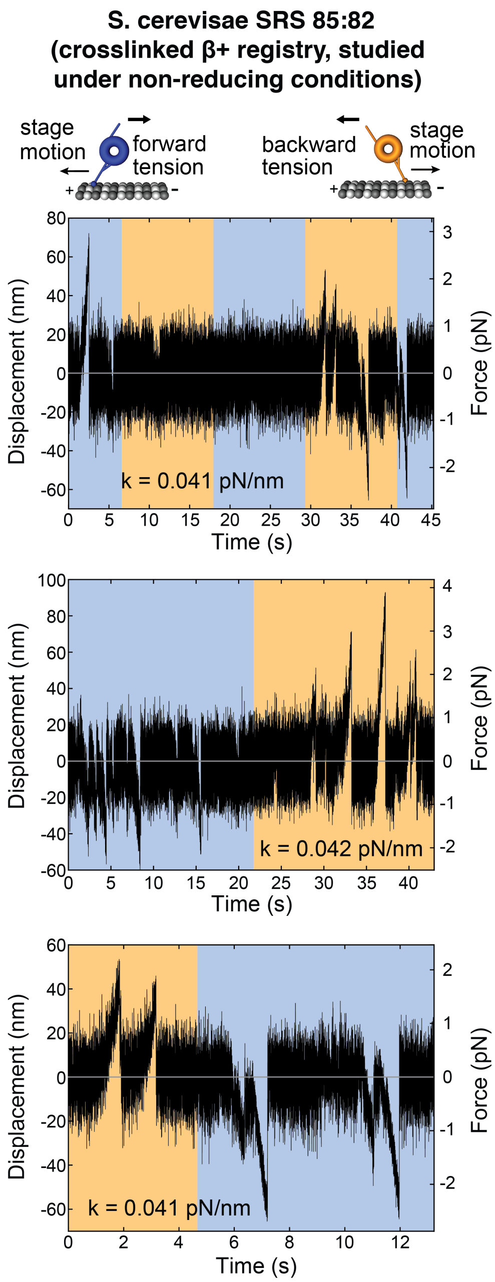

Supplementary Figure 25 Unbinding-force experiments (force/position vs. time) for the SRS-α stalk-MTBD CL construct with the *S. cerevisiae* stalk-MTBD sequence and cross-linked β-registry. The illustration represents the configuration for backward vs. forward tension. Orange (blue) shaded areas show periods of applied rearward (forward) tension.

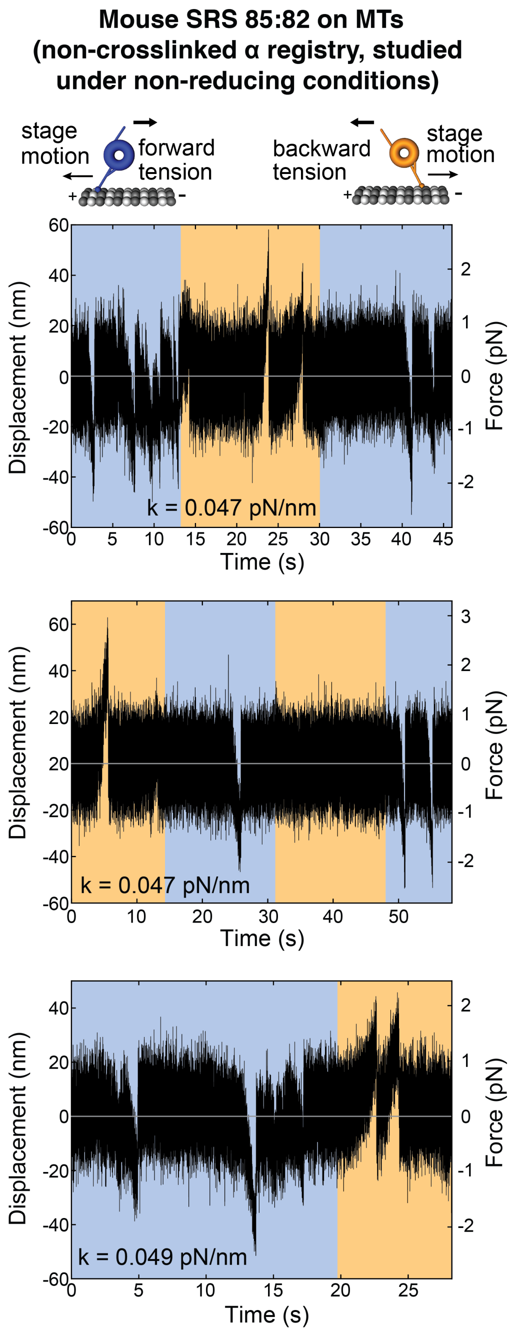

Supplementary Figure 26 Unbinding-force experiments (force/position vs. time) for the mouse SRS-α stalk-MTBD construct with non-cross-linked stalk helices. The illustration represents the configuration for backward vs. forward tension. Orange (blue) shaded areas show periods of applied rearward (forward) tension.

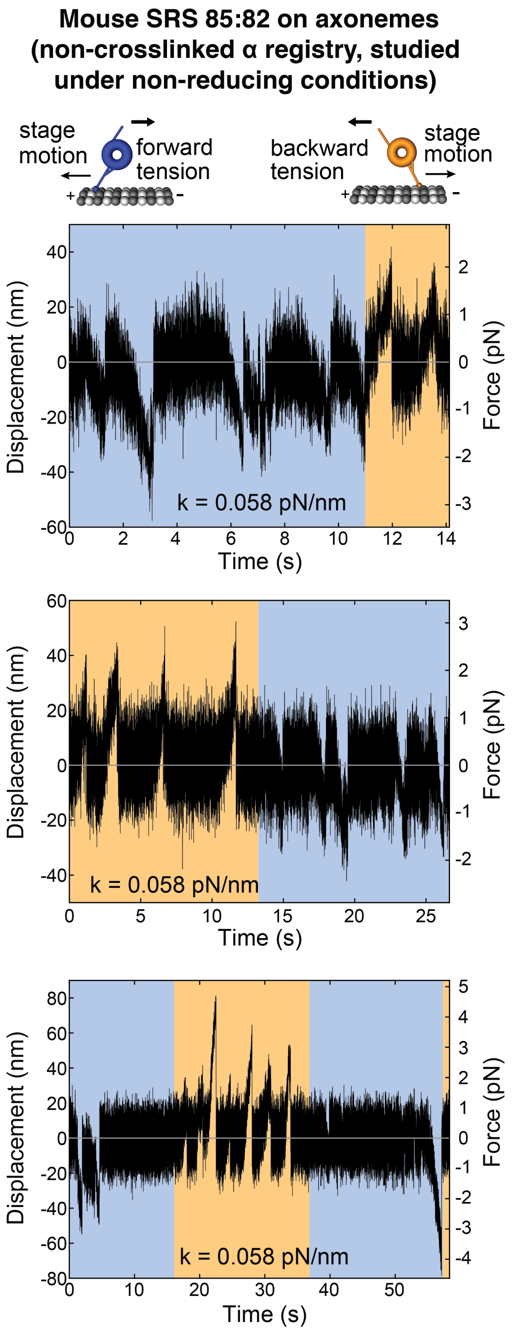

Supplementary Figure 27 Unbinding-force experiments (force/position vs. time) for the mouse SRS-α stalk-MTBD construct with non-cross-linked stalk helices using axonemes. The illustration represents the configuration for backward vs. forward tension. Orange (blue) shaded areas show periods of applied rearward (forward) tension.

Conversion of WT Apo unbinding-force histograms

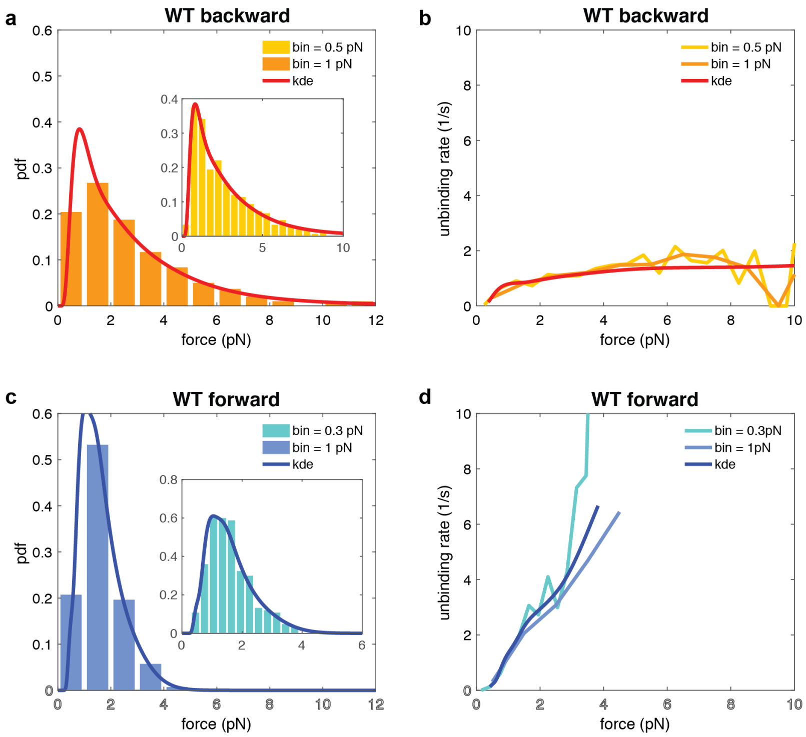

Supplementary Figure 28 Comparison of the histogram-based method derived by Dudko and co-workers[7] with our improved method using kernel-density estimators shown for the WT motor domain, Dyn1$_{331kDa}$. Both methods determine a probability density function (PDF) from the unbinding force data (*a* and *c*) that is used to calculate the corresponding force-dependent unbinding rates (*b* and *d*). The kernel density estimator (KDE) (solid dark red and blue lines in *a* and *c*) result in a smooth unbinding rates (solid red and dark blue lines in *b* and *d*). The histogram-based method depends on the bin size: increasing the bin size results in an oscillating, non-smooth unbinding rate, because the contour of the histograms no longer reflects accurately the underlying distribution (histograms with larger numbers of bins are shown in the insets of *a* and *c* and the

corresponding unbinding rates are displayed in *b* and *d* using the color code depicted in *a* and *b*, respectively).

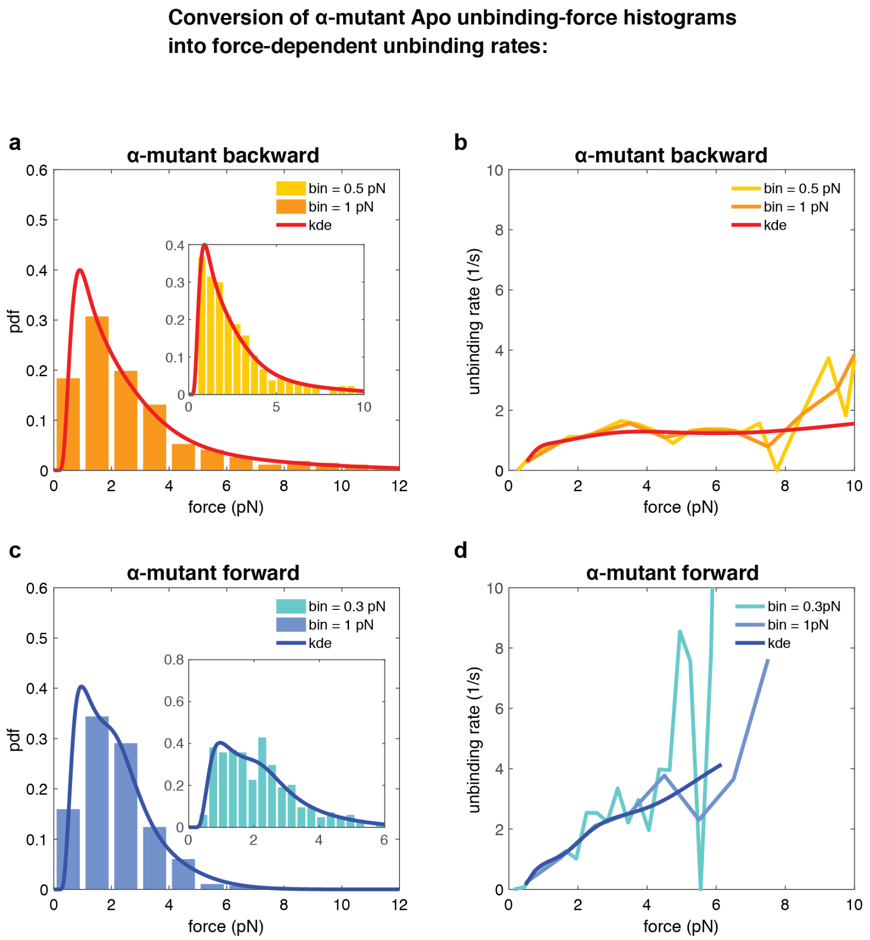

Supplementary Figure 29 As in Supplementary Fig. 28 but for the α-mutant with the cross-linked stalk helices.

Conversion of β-mutant Apo unbinding-force histograms into force-dependent unbinding rates:

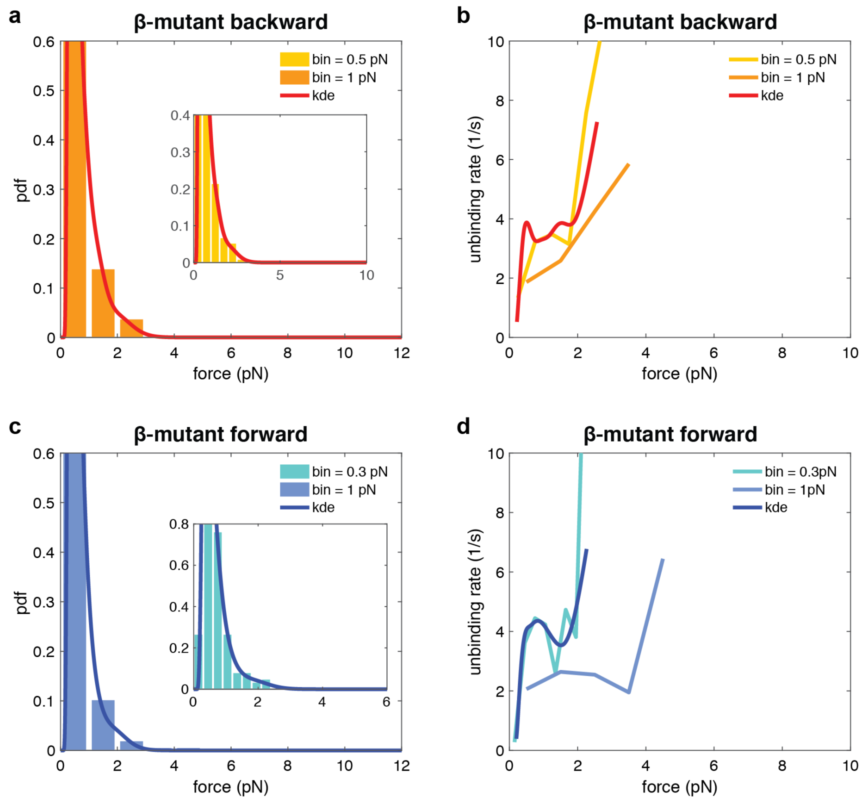

Supplementary Figure 30 As in Supplementary Fig. 28 but for the β-mutant with the cross-linked stalk helices.

Conversion of γ-mutant Apo unbinding-force histograms into force-dependent unbinding rates:

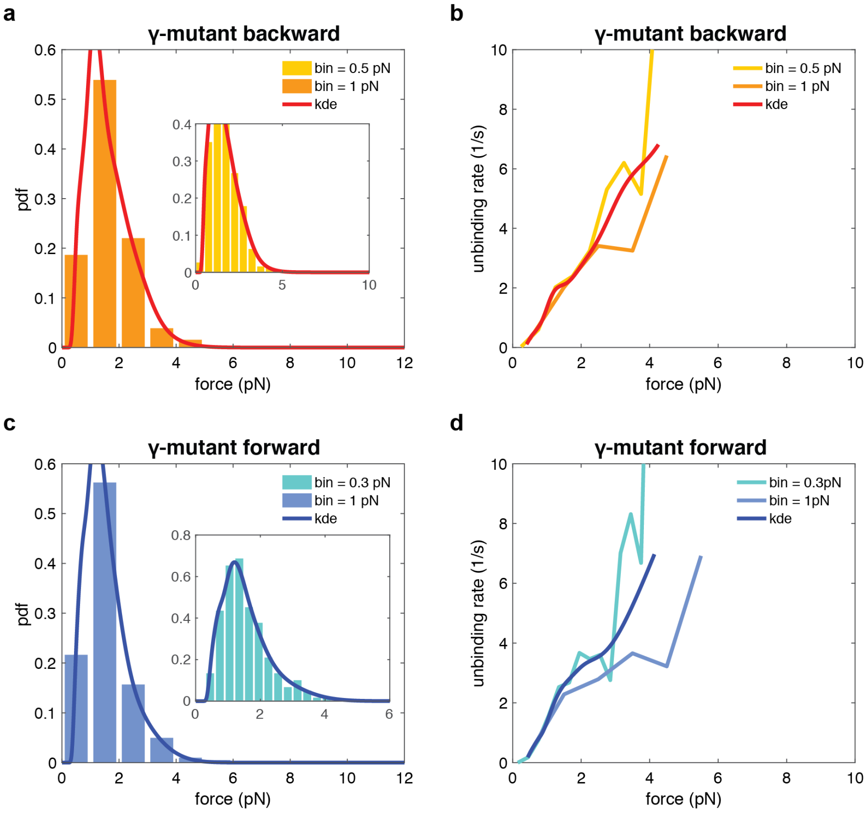

Supplementary Figure 31 As in Supplementary Fig. 28 but for the γ-mutant with the cross-linked stalk helices.

Construct	Description	Genotype	Source
VY137	WT $Dyn1_{331kDa}$	*pGAL-ZZ-TEV-GFP-3xHA-331DYN1*	Vale lab
VY863	AAA1 E/Q - $Dyn1_{331kDa}$	*pGAL-ZZ-TEV-GFP-3xHA-331DYN1 (E1849Q)*	Vale lab
GY1	$Dyn1_{331kDa}$-α	*pGAL-ZZ-TEV-GFP-3xHA-331DYN1 (K3077C, A3250C)*	This study
GY2	$Dyn1_{331kDa}$-β	*pGAL-ZZ-TEV-GFP-3xHA-331DYN1 (I3076C, L3247C)*	This study
GY60	AAA1 E/Q-$Dyn1_{331kDa}$-α	*pGAL-ZZ-TEV-GFP-3xHA-331DYN1 (E1849Q, K3077C, A3250C)*	This study
GY67	ΔBUT-$Dyn1_{331kDa}$	*pGAL-ZZ-TEV-GFP-3xHA-331DYN1 (aa3556-3596 replaced with GSGS)*	This study
GY68	ΔBUT-$Dyn1_{471kDa}$	*PAC11-13xMyc::TRP, nip100Δ::NAT, ZZ-TEV-GFP-GS-HaloTag-GS-3xHA-DYN1(aa3556-3596 replaced with GSGS)*	This study
GY71	ΔBUT-E3197K-$Dyn1_{331kDa}$	*pGAL-ZZ-TEV-GFP-3xHA-331DYN1 (E3197K, aa3556-3596 replaced with GSGS)*	This study
GY201	F3446E-$Dyn1_{331kDa}$	*pGAL-ZZ-TEV-GFP-3xHA-331DYN1 (F3446E)*	This study
GY205	F3446E-AAA3 E/Q + C-term GFP	*pGAL-ZZ-TEV-3xHA-331DYN1 (E2488Q, F3446E)-GFP*	This study
GY207	$Dyn1_{331kDa}$-γ mutant	*pGAL-ZZ-TEV-GFP-3xHA-331DYN1 (K3077C, L3247C)*	This study
GY231	ΔBUT-$Dyn1_{331kDa}$-α	*pGAL-ZZ-TEV-GFP-3xHA-331DYN1 (K3077C, A3250C, aa3556-3596 replaced with GSGS)*	This study
GY243	$Dyn1_{331kDa}$ with N-term SNAPf-tag	*pGAL-ZZ-TEV-GFP-SNAPf-3xHA-331DYN1*	This study

GY242	Dyn1$_{331kDa}$-β with N-term SNAPf-tag	*pGAL-ZZ-TEV-GFP-SNAPf-3xHA-331DYN1 (I3076C, L3247C)*	This study
GY247	AAA1 E/Q - Dyn1$_{331kDa}$ with N-term SNAPf-tag	*pGAL-ZZ-TEV-GFP-SNPAf-3xHA-331DYN1 (E1849Q)*	This study
GY246	ΔBUT-Dyn1$_{331kDa}$ with N-term SNAPf-tag	*pGAL-ZZ-TEV-GFP-SNAPf-3xHA-331DYN1 (aa3556-3596 replaced with GSGS)*	This study
pFC15	SRS mouse dynein stalk MTBD 85:82	SRS-mouse dynein stalk MTBD 85:82-EGFP-6xHis	Cleary et al. 2014, Nat. Commun.
pSYS01	SRS yeast dynein stalk MTBD 85:82	SRS-yeast dynein stalk MTBD 85:82-EGFP-6xHis	This study
pSYS02	SRS yeast dynein stalk MTBD 89:82	SRS-yeast dynein stalk MTBD 89:82-EGFP-6xHis	This study
pSYS03	SRS yeast dynein stalk MTBD 85:82-α	SRS-yeast dynein stalk MTBD 85:82 (K3077C, A3250C) -EGFP-6xHis	This study
pSYS04	SRS yeast dynein stalk MTBD 85:82-β	SRS-yeast dynein stalk MTBD 85:82 (I3076C, L3247C) -EGFP-6xHis	This study
pSYS05	SRS yeast dynein stalk MTBD 89:82-β	SRS-yeast dynein stalk MTBD 89:82 (I3076C, L3247C) -EGFP-6xHis	This study

Table S1: Yeast strains and SRS constructs used in this work. "331DYN1" encodes amino acids 1219-4092 of Dyn1, with predicted molecular weight of 331 kDa (see Reck-Peterson et al. Cell 2016, and remark in footnote on page 8, section "Yeast culture and dynein purification" in Nicholas et al. PNAS 2015), and WT ("wild type") represents the tail-truncated, single-headed dynein construct without AAA mutation. All yeast strains are based on W303 (*MATa/MATα {leu2-3,112 trp1-1 can1-100 ura3-1 ade2-1 his3-11,15} [phi+]*) plus *pep4Δ::HIS3, prb1Δ*. Except for GY68, all yeast strains express single-headed dyneins.

**URA primers**	
u1F	gtgattctgggtagaagatcgg
u2R	gagcaatgaacccaataacgaaatc
u3F	cttgacgttcgttcgactgatgagc
u4R	cgatgatgtagtttctggtttttaa
**URA insertion in stalk for GY1, GY2, GY60, GY207 & GY231**	
Stalk-F-up	TGGAGAAATTGCCAAAAACCTTCA
Stalk-R-URA	ccgatcttctacccagaatcacAGCTTCACTAAGGCACGTAAACC
Stalk-F-URA	ttaaaaaccagaaactacatcatcgCTGATTGGAAATTGCATAATTTCAT
Stalk-R-down	ACCAATCTCCACCGTGACTCTG
**GY1 & GY60**	
Stalk-a1-R	AATATCTTTTTgcaTTCCTCTGTAGCCTC
Stalk-a1-F	GAGGCTACAGAGGAAtgcAAAAAGATATT
Stalk-a2-R	TCATTTCTTCAGCGGCAAGacaATTGG
Stalk-a2-F	CCAATtgtCTTGCCGCTGAAGAAATGA
**GY2**	
Stalk-b1-R	AATATCTTacaGATTTCCTCTGTAGCCTC
Stalk-b1-F	GAGGCTACAGAGGAAATCtgtAAGATATT
Stalk-b2-R	TCATTTCTTCacaGGCAAGTAAATTGG
Stalk-b2-F	CCAATTTACTTGCCtgtGAAGAAATGA
**GY207**	
Stalk-a1-R	AATATCTTTTTgcaTTCCTCTGTAGCCTC
Stalk-a1-F	GAGGCTACAGAGGAAtgcAAAAAGATATT
Stalk-b2-R	TCATTTCTTCacaGGCAAGTAAATTGG
Stalk-b2-F	CCAATTTACTTGCCtgtGAAGAAATGA
**URA insertion in buttress for GY67**	
But-F-up	GGATGGAGAATTCTTTGATCCAATC
But-R-URA	ccgatcttctacccagaatcacATTCTCTTCTGTCAAAGTGATGTC
But-F-URA	ttaaaaaccagaaactacatcatcgTATTCTATTATCGGTAAGCATAGTG
But-R-down	GTCAAGAGCGGTGGAGAAT
**GY67 & GY68**	
But-del-R	TTTTTCAATagaaccagaaccGTATTCGGTATTCAATTTTATCAA

But-del-F	ACCGAATACggttctggttctATTGAAAAAAAGCTAAGTGAATCT
**URA insertion in MTBD for GY71**	
MTBD-F-up	CCAACTGGAGAGATATCCAACAA
MTBD-R-URA	ccgatcttctacccagaatcacATAAGTAAAATTAGGATCTGACAA
MTBD-F-URA	ttaaaaaccagaaactacatcatcgACAATTAACAGGGCCAGCAA
MTBD-R-down	TAGCCTGCACGTTACTCATTT
**GY71**	
MTBD-EK-R	GCCCTGTTAATTGTtttATAAGTAAAA
MTBD-EK-F	TTTTACTTATaaaACAATTAACAGGGC
**URA insertion in AAA5 for GY201 & GY205**	
AAA5-F	CTTGAGTGTGGCTTGGATAAGA
AAA5-R-URA	ccgatcttctacccagaatcacTCTAACTGCATTTTCTAATCTTTT
AAA5-F-URA	ttaaaaaccagaaactacatcatcgGAAGGAAGTGTAGTTATAATTCAGGAT
AAA5-R	TGGGATCGCAAGAGTGAATG
**GY201 & GY205**	
AAA5-FE-R	TACACTTCCttcTCTAACTGC
AAA5-FE-F	GCAGTTAGAgaaGGAAGTGTA
**Amplification of SNAPf-tag (S. cerevisiae codon optimized)**	
SNAPf-F	ATGGATAAGGACTGCGAAATGAA
SNAPf-R	ACCCAAACCTGGTTTACCTAAT
**GY242, GY243, GY246 & GY247**	
DYN314-F-up	GTTTACATCATGGCTGACAAACA
DYN314-R-down	TGTTTGTCAGCCATGATGTAAAC
GFP-SG-R	cggcggcttctaatccgtATTACCCTGTTATCCCTATCTAGATTTGTACAATTCATCCAT

Gal-F	acggattagaagccgccga
R-Sce-URA	ttatgtgagagtttaaaaaccagaaactacatcatcgTTATTTCAGGAAAGTTTC GGAGG
u1F-RV	cgatgatgtagtttctggttttta
u2R-RV	CTTGACGTTCGTTCGACTGATGAGC
u3F-RV	GAGCAATGAACCCAATAACGAAATC
u4R-RV	gtgattctgggtagaagatcgg
GFP-URA-F	ccgatcttctacccagaatcacGTTTACATCATGGCTGACAAACA
GFP-SNAPf-R	TTCATTTCGCAGTCCTTATCCATACTACCTCTAGATTTGTACAAT TCATCCATAC
SNAPf-HA-F	ATTAGGTAAACCAGGTTTGGGTGGTTCATACCCATACGATGTT CCTGAC
**Replacement of mouse dynein stalk-MTBD with S. cerevisiae dyneins talk-MTBD in pFC15**	
SRS-F	CTTGAGGCACTTCTCCTGC
SRS-R	GAGCCATGAAGTctgcagCGAAACGTAACAGTGCTTCTTC
Sc85-F	GCACTGTTAGCTgtcgacGTAGGTCTCGAAAAACTAAACG
Sc89-F	GCACTGTTAGCTgtcgacAGATTTGTTAATGTAGGTCTCGAAA
Sc82-R	GCAGGAGAAGTGCCTCAAGAGTGTTCAGCCACCTTTCTTTTTC
**CC1 – α mutation**	
CC1a-F	AGAGGAAATCtgcAAGATATTGAAAGTACAAGAAG
CC1a-R	GTAGCCTCCTGCTTTCTTTC
**CC2 – α mutation**	
CC2a-F	TTTACTTGCCtgtGAAGAAATGACACAGG
CC2a-R	TTGGCCTTCGTCTTCAATG
**CC1 – β mutation**	
CC1b-F	TACAGAGGAAtgcAAAAAGATATTGAAAGTACAAG
CC1b-R	GCCTCCTGCTTTCTTTCC
**CC2 – β mutation**	
CC2b-F	GAAGGCCAATtgtCTTGCCGCTG

CC2b-R	GTCTTCAATGATTCGAACTCTATTC

Table S2: List of primers used for the generation of yeast strains and SRS constructs.

Supplementary References

1. Gutiérrez-Medina, B., Fehr, A.N. & Block, S.M. Direct measurements of kinesin torsional properties reveal flexible domains and occasional stalk reversals during stepping. *Proc Natl Acad Sci USA* **106**, 17007-17012 (2009).
2. Cleary, F.B. et al. Tension on the linker gates the ATP-dependent release of dynein from microtubules. *Nat Commun* **5**, 4587 (2014).
3. Svoboda, K. & Block, S.M. Biological applications of optical forces. *Annu Rev Biophys Biomol Struct* **23**, 247-285 (1994).
4. Nicholas, M.P., Rao, L. & Gennerich, A. An improved optical tweezers assay for measuring the force generation of single Kinesin molecules. *Methods Mol Biol* **1136**, 171-246 (2014).
5. Ray, C., Brown, J.R. & Akhremitchev, B.B. Correction of systematic errors in single-molecule force spectroscopy with polymeric tethers by atomic force microscopy. *J Phys Chem B* **111**, 1963-1974 (2007).
6. Munoz, V. & Cerminara, M. When fast is better: protein folding fundamentals and mechanisms from ultrafast approaches. *Biochem J* **473**, 2545-2559 (2016).
7. Dudko, O.K., Hummer, G. & Szabo, A. Theory, analysis, and interpretation of single-molecule force spectroscopy experiments. *Proc Natl Acad Sci USA* **105**, 15755-15760 (2008).
8. Nicholas, M.P. et al. Cytoplasmic dynein regulates its attachment to microtubules via nucleotide state-switched mechanosensing at multiple AAA domains. *Proc Natl Acad Sci USA* **112**, 6371-6376 (2015).
