# Supplementary material for: Molecular mechanism of cytoplasmic dynein tension sensing
Source: Nat Commun. 2019 Jul 26;10:3332. doi: 10.1038/s41467-019-11231-8 (PMC6659695; doi:10.1038/s41467-019-11231-8)
Supplement: Supplementary file 2 — Reporting Summary [file 41467_2019_11231_MOESM2_ESM.pdf]

nature research

Corresponding author(s): Arne Gennerich

Last updated by author(s): May 20, 2019

Reporting Summary

Nature Research wishes to improve the reproducibility of the work that we publish. This form provides structure for consistency and transparency in reporting. For further information on Nature Research policies, see Authors & Referees and the Editorial Policy Checklist.

Statistics

For all statistical analyses, confirm that the following items are present in the figure legend, table legend, main text, or Methods section.

n/a	Confirmed	
☐	☒	The exact sample size (*n*) for each experimental group/condition, given as a discrete number and unit of measurement
☐	☒	A statement on whether measurements were taken from distinct samples or whether the same sample was measured repeatedly
☐	☒	The statistical test(s) used AND whether they are one- or two-sided<br>*Only common tests should be described solely by name; describe more complex techniques in the Methods section.*
☐	☒	A description of all covariates tested
☐	☒	A description of any assumptions or corrections, such as tests of normality and adjustment for multiple comparisons
☐	☒	A full description of the statistical parameters including central tendency (e.g. means) or other basic estimates (e.g. regression coefficient) AND variation (e.g. standard deviation) or associated estimates of uncertainty (e.g. confidence intervals)
☐	☒	For null hypothesis testing, the test statistic (e.g. *F*, *t*, *r*) with confidence intervals, effect sizes, degrees of freedom and *P* value noted<br>*Give P values as exact values whenever suitable.*
☒	☐	For Bayesian analysis, information on the choice of priors and Markov chain Monte Carlo settings
☒	☐	For hierarchical and complex designs, identification of the appropriate level for tests and full reporting of outcomes
☒	☐	Estimates of effect sizes (e.g. Cohen's *d*, Pearson's *r*), indicating how they were calculated

Our web collection on statistics for biologists contains articles on many of the points above.

Software and code

Policy information about availability of computer code

Data collection	A custom-built optical tweezers setup was used and data collection was controlled using a custom-written LabVIEW program as described previously in Nicholas et al. 2014 (Methods Mol. Biol.).
Data analysis	Data were analyzed using a custom-written MATLAB program as previously described in Nicholas et al. 2015 (PNAS).

For manuscripts utilizing custom algorithms or software that are central to the research but not yet described in published literature, software must be made available to editors/reviewers. We strongly encourage code deposition in a community repository (e.g. GitHub). See the Nature Research guidelines for submitting code & software for further information.

Data

Policy information about availability of data

All manuscripts must include a data availability statement. This statement should provide the following information, where applicable:

- Accession codes, unique identifiers, or web links for publicly available datasets
- A list of figures that have associated raw data
- A description of any restrictions on data availability

The datasets generated during and/or analyzed during the current study are available from the corresponding author on reasonable request.

Field-specific reporting

Please select the one below that is the best fit for your research. If you are not sure, read the appropriate sections before making your selection.

☒ Life sciences ☐ Behavioural & social sciences ☐ Ecological, evolutionary & environmental sciences

October 2018

For a reference copy of the document with all sections, see nature.com/documents/nr-reporting-summary-flat.pdf

Life sciences study design

All studies must disclose on these points even when the disclosure is negative.

Sample size	Sample sizes were determined to be able to draw statistically meaningful conclusion as previously described in Nicholas et al. 2015 (PNAS).
Data exclusions	As stated in the Supplemental Material of the manuscript, under non-reducing conditions to promote cross-linking of the stalk helices, 2 out of 40 beads coated with the β mutant and 1 out of 26 beads coated with the γ mutant exhibited the anisotropic unbinding behavior as seen for the WT motor. These 3 beads were excluded from the analysis presented in the main text.
Replication	For reproducibility, every genome was sequenced and all constructs were assayed from at least two independent preparations.
Randomization	N.A.
Blinding	Data acquisition was automated in the absence of possible user bias.

Reporting for specific materials, systems and methods

We require information from authors about some types of materials, experimental systems and methods used in many studies. Here, indicate whether each material, system or method listed is relevant to your study. If you are not sure if a list item applies to your research, read the appropriate section before selecting a response.

Materials & experimental systems

n/a	Involved in the study	
☐	☒	Antibodies
☒	☐	Eukaryotic cell lines
☒	☐	Palaeontology
☒	☐	Animals and other organisms
☒	☐	Human research participants
☒	☐	Clinical data

Methods

n/a	Involved in the study	
☒	☐	ChIP-seq
☒	☐	Flow cytometry
☒	☐	MRI-based neuroimaging

Antibodies

Antibodies used	Anti-GFP antibodies were purified from rabbit serum as previously described by Nicholas et al. 2015 (PNAS).
Validation	Antibodies were purified using an affinity column bearing purified GFP with a GST (glutathione S-transferase) tag (GSTGFP) and the GFP-tagged constructs used in this study only bound to trapping beads when coated with the purified anti-GFP antibody.
